# Supplementary material for: Positional distribution of transcription factor binding sites in the human genome
Source: PLoS One. 2025 Jul 30;20(7):e0329226. doi: 10.1371/journal.pone.0329226 (PMC12310040; doi:10.1371/journal.pone.0329226)

S1 Fig: analysis flowchart

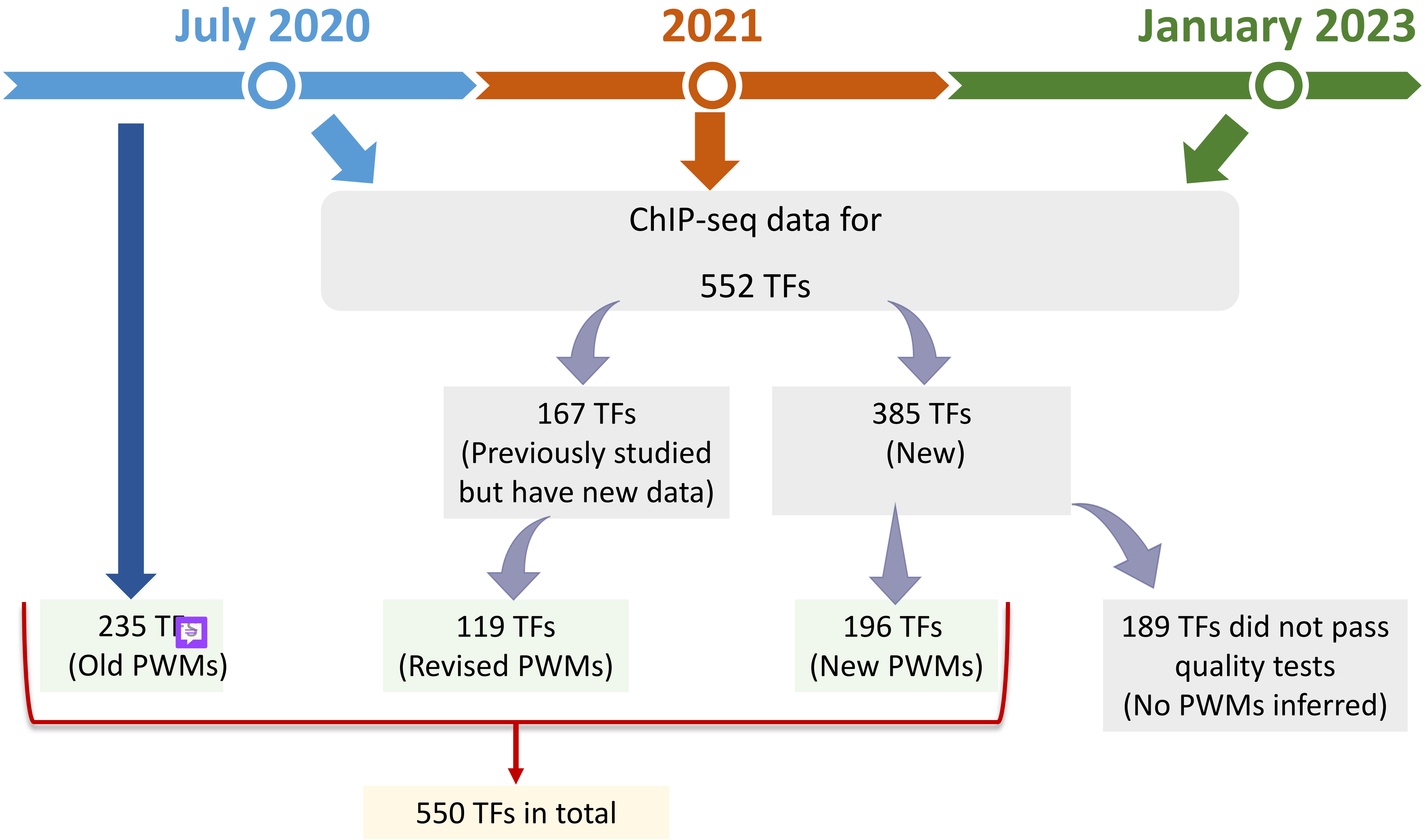

S2 Fig: Core motifs

C2H2-ZF

- Total of canonical PWMs: 244

Core1: 33 PWMs

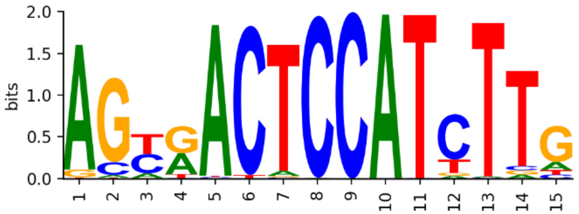

Core2: 11 PWMs

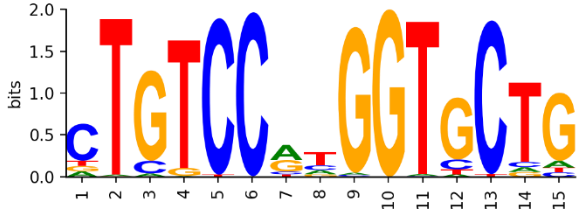

Core3: 9 PWMs

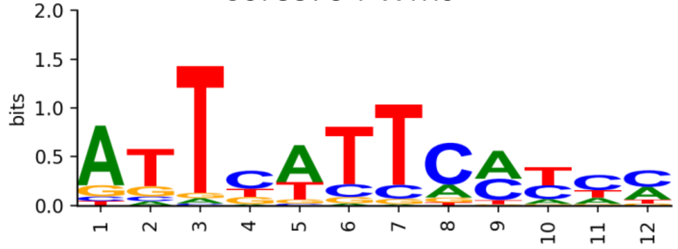

Core4: 7 PWMs

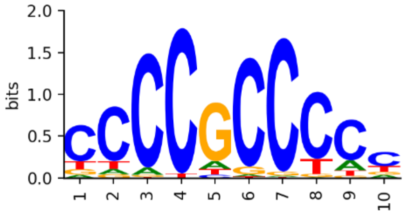

Core5: 7 PWMs

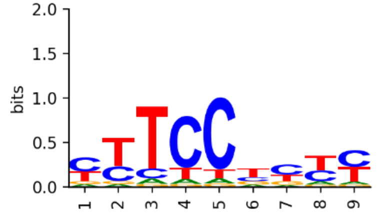

Core6: 7 PWMs

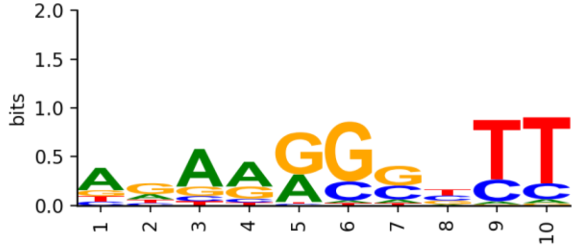

Core7: 6 PWMs

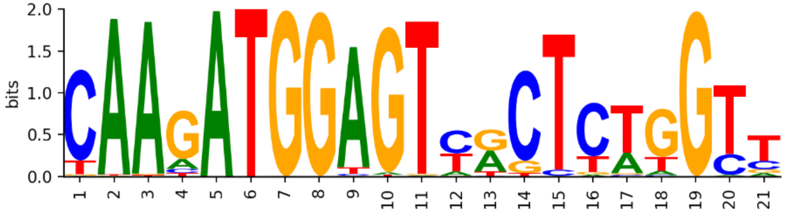

Core8: 5 PWMs

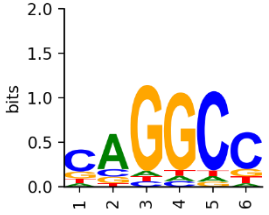

Core9: 5 PWMs

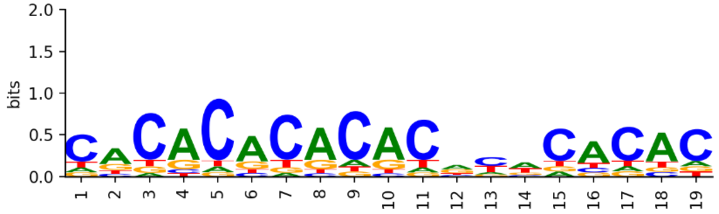

Homeodomain

- Total of canonical PWMs: 27

Core1: 8 PWMs

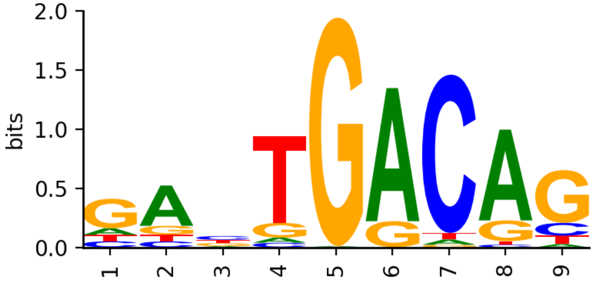

Core2: 7 PWMs

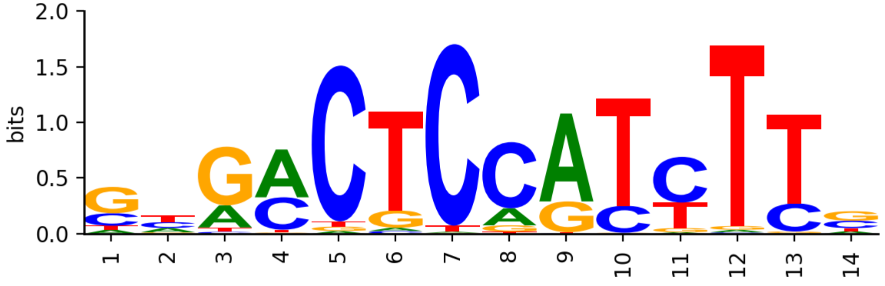

Core3: 6 PWMs

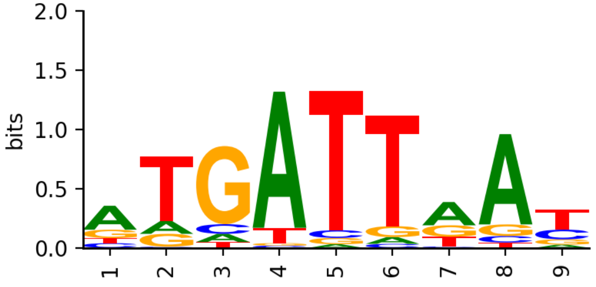

Core4: 5 PWMs

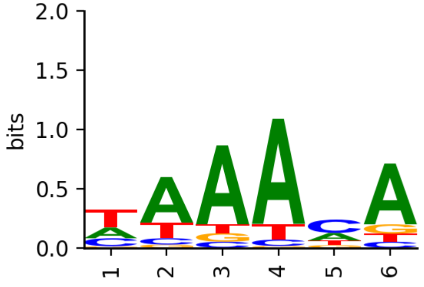

bHLH Total of canonical PWMs: 26

Core1: 15 PWMs

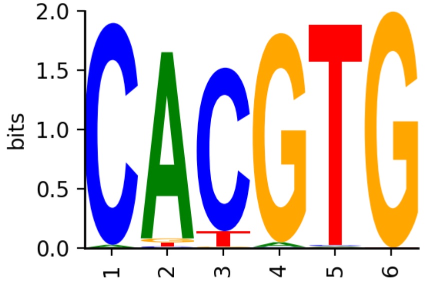

Core2: 5 PWMs

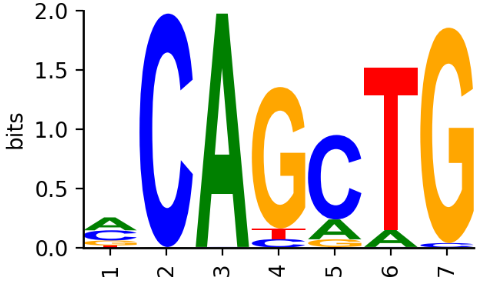

Core3: 4 PWMs

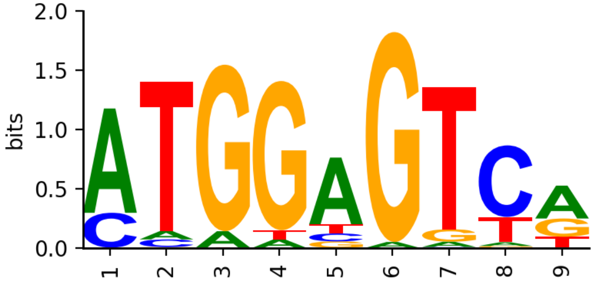

bZIP

- Total of canonical PWMs: 33

Core1: 15 PWMs

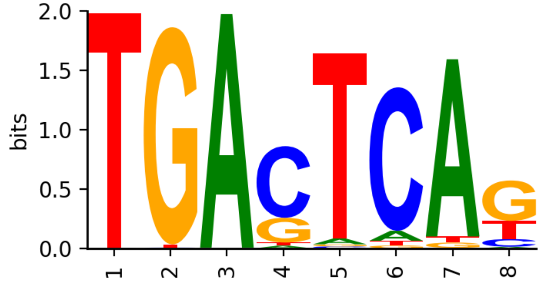

Core2: 7 PWMs

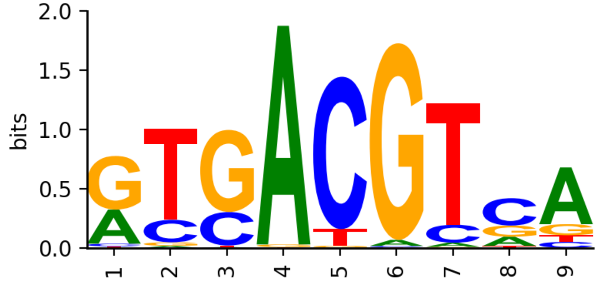

Core3: 9 PWMs

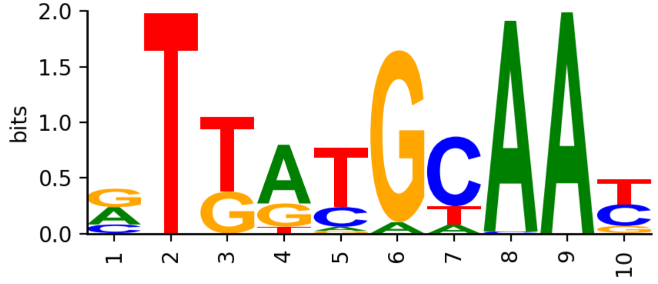

Forkhead

- Total of canonical PWMs: 14

Core 1: 13 PWMs

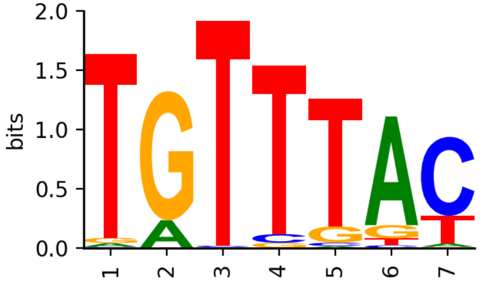

Ets

- Total of canonical PWMs: 14

Core 1: 13 PWMs

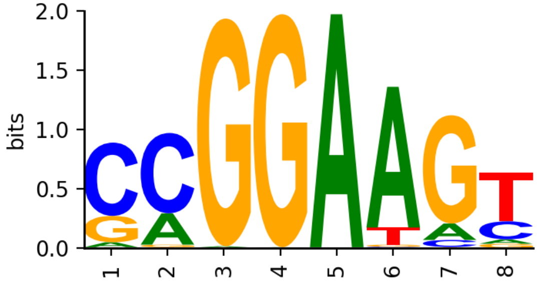

GATA

- Total of canonical PWMs: 5

Core 1: 5 PWMs

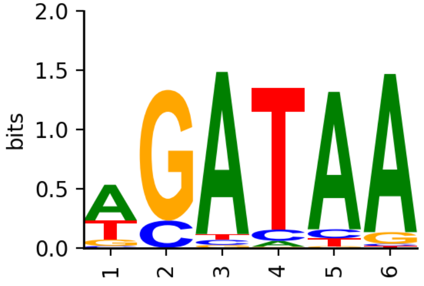

E2F

- Total of canonical PWMs: 8

Core 1: 7 PWMs

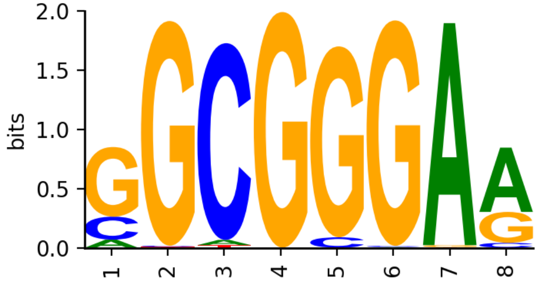

CUT; Homeodomain

- Total of canonical PWMs: 3

Core 1: 3 PWMs

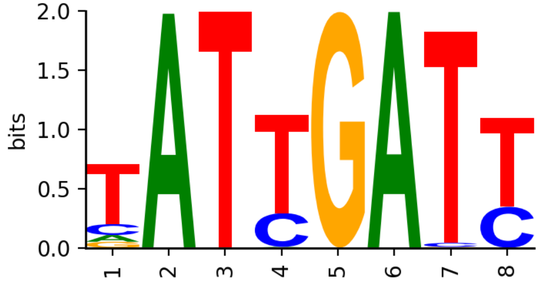

STAT

- Total of canonical PWMs: 3

Core 1: 3 PWMs

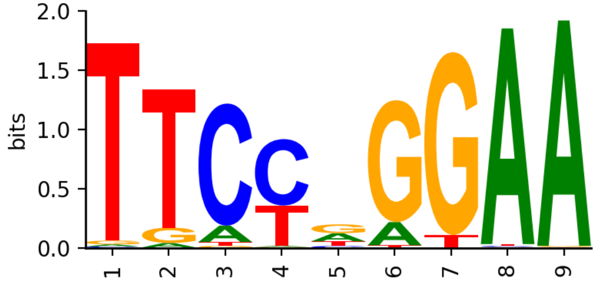

RFX

- Total of canonical PWMs: 3

Core 1: 3 PWMs

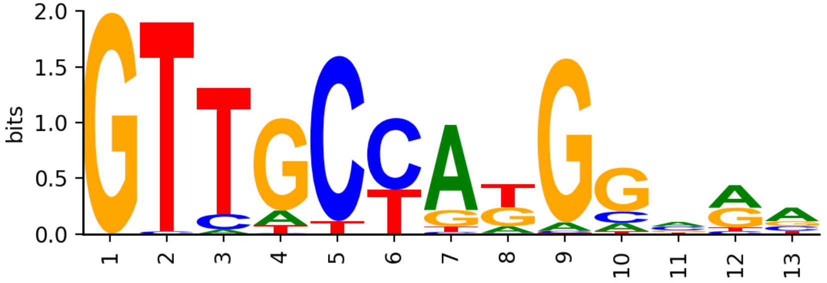

Nuclear receptor

- Total of canonical PWMs: 21

Core 1: 14 PWMs

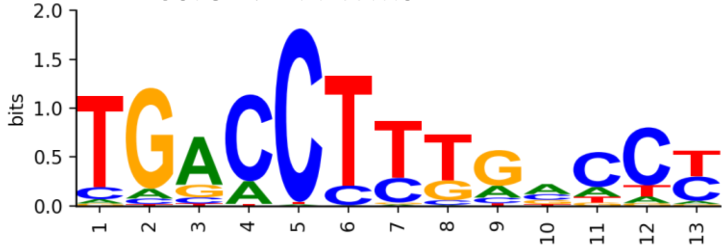

Core 2: 3 PWMs

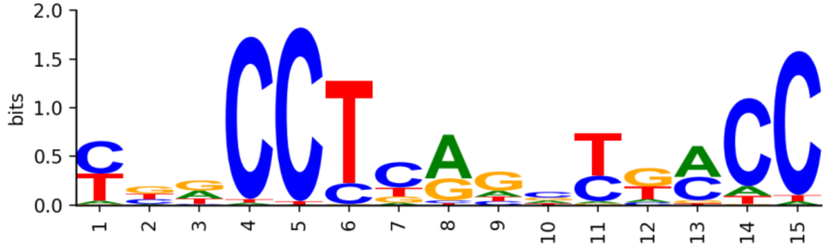

Rel

- Total of canonical PWMs: 4

Core 1: 4 PWMs

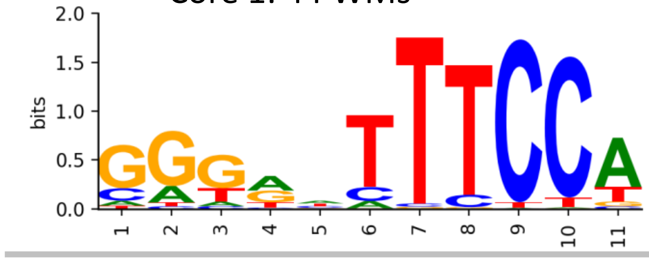

IRF

- Total of canonical PWMs: 4

Core: 4 PWMs

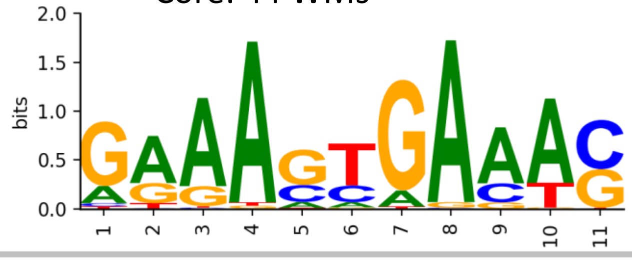

TEA

- Total of canonical PWMs: ?

Core 1: ? PWMs

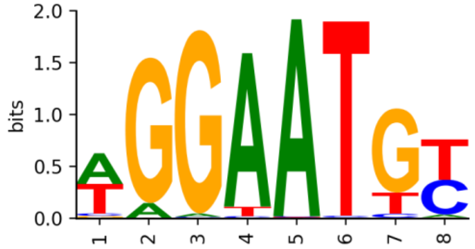

MADS box

- Total of canonical PWMs: 4

Core: 4 PWMs

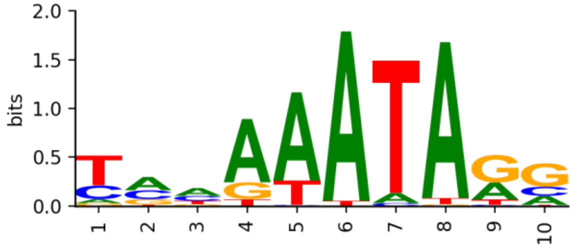

SMAD

- Total of canonical PWMs: 7

Core: 5 PWMs

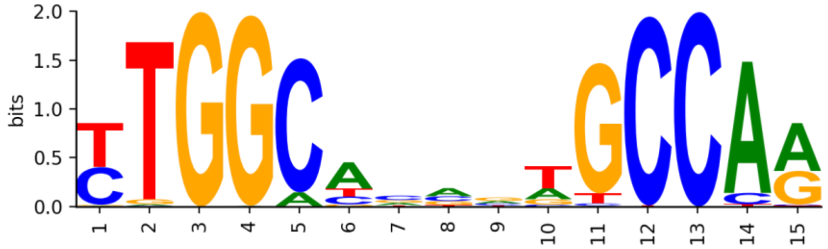

Unknown

- Total of canonical PWMs: 6

Core: 3 PWMs

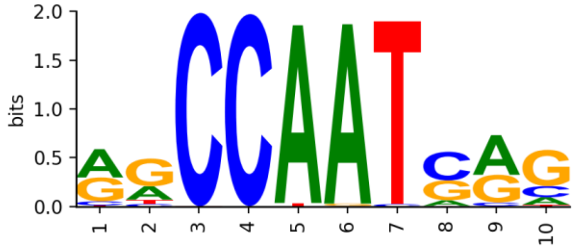

HMG/Sox

- Total of canonical PWMs: 7

Core: 7 PWMs

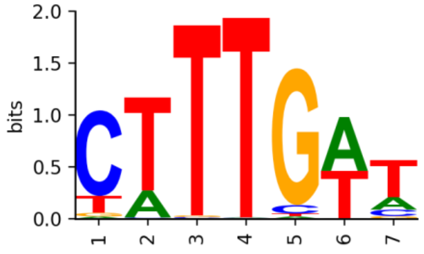

S3 Fig. Co-binding analysis. Computing the counts of canonical binding, tethered binding and co-binding in a set of ChIP-seq data

Method

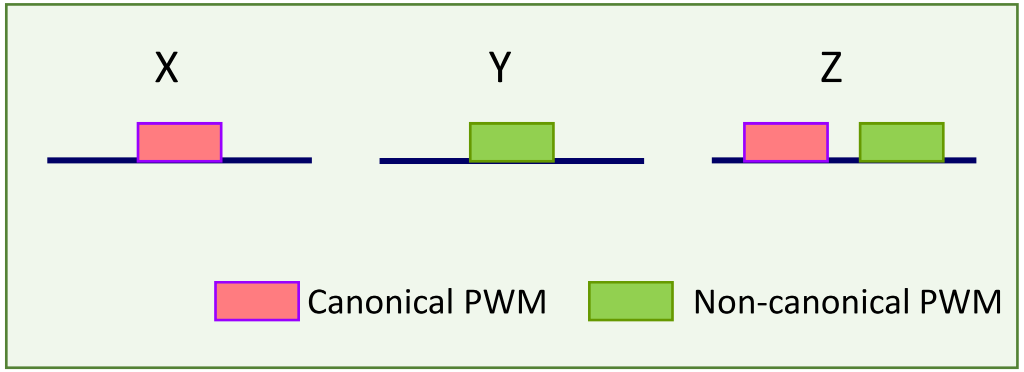

- In the above figure, there are three types of the ChIP-seq peaks: (1) The peak contains only a canonical motif (a red rectangle), (2) the peak contains only a non-canonical motif (a green rectangle), and (3) the peak contains a canonical motif and a non-canonical motif.
- From a set of ChIP-seq data for a TF, one can compute the following counts:
  - X: Number of peaks containing only the canonical motif.
  - Y: Number of peaks each containing at least one non-canonical (co-occurring) motif but no canonical motif.
  - Z: Number of peaks each containing both the canonical PWM and a non-canonical PWM.
- Note:
  - A non-canonical PWM is a PWM that is similar to the canonical PWM of a TF that belong to another TF family.
  - The presence of a motif is determined using the PWM and FIMO with a p-value < 0.0001 to screen the genome; a FIMO hit is retained only if it overlaps with a ChIP-seq peak.

Classification of TFs into five groups

1) $X>0$, $Y = Z = 0$: canonical binding only
2) $X > Y > Z$: canonical binding frequency > tethered binding frequency > co-binding frequency
3) $X > Z > Y$: canonical binding frequency > co-binding frequency > tethered binding frequency
4) $Y > X$ and $Y > Z$: tethered binding most frequent
5) $Z > X$ and $Z > Y$: co-binding most frequent

Pie chart of the five groups of TFs

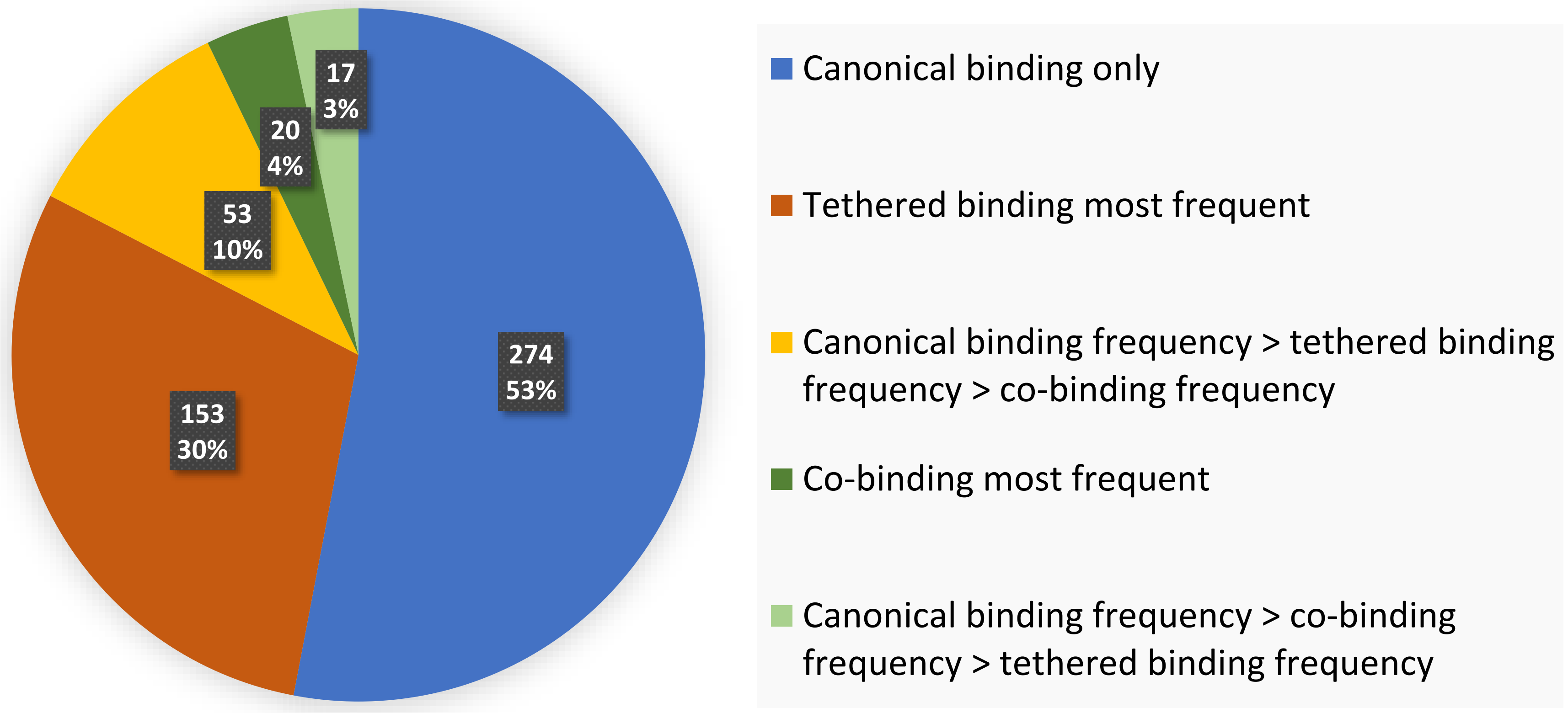

S4 Fig. Identification of canonical PWN for a TF by the ranking method

Inferred PWMs from ChIP-seq data for **GATA3**

Experiment	Top		Second	
T47D (ENCSR000BMX)	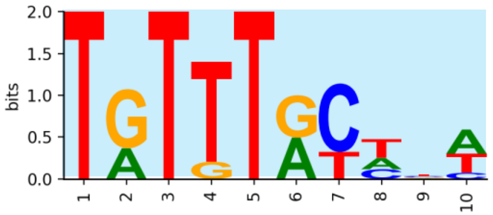	184 peaks FOXC2 (PCC=0.896)	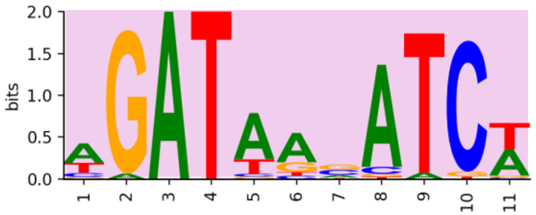	113 peaks GATA3 (PCC=0.779)
SK-N-SH (ENCSR000BTH)	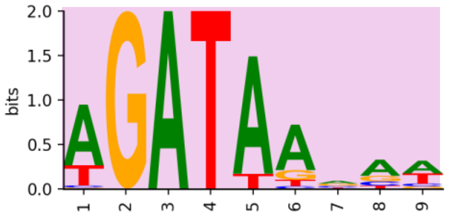	451 peaks GATA3 (PCC=0.952)	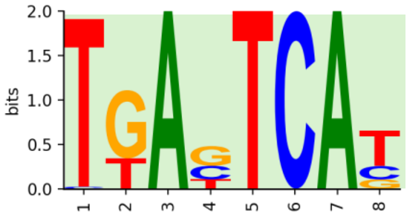	169 peaks NFE2 (PCC=0.931)
MCF-7 (ENCSR423RTK)	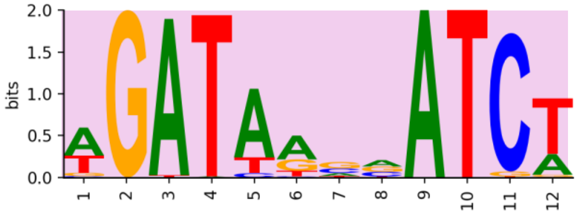	146 peaks GATA3 (PCC=0.732)	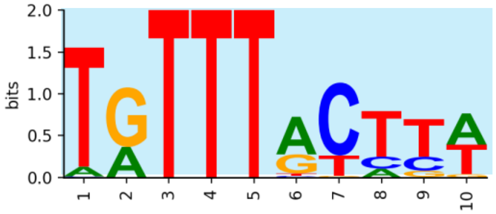	121 peaks FOXC2 (PCC=0.932)
MCF-7 (ENCSR000EWS)	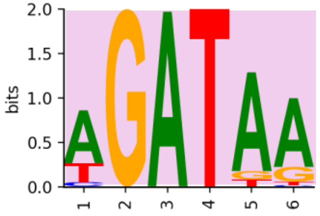	344 peaks GATA3 **(PCC=0.963)**		
**Multiple experiments (ranking)**	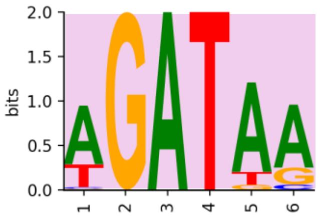	669 peaks GATA3 **(PCC=0.963)**	No secondary	

Reference

(A) GATA3 (SELEX: M03063_2.00)

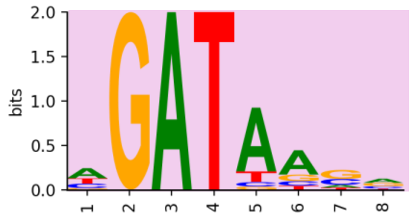

(B) FOXC2 (SELEX: M03038_2.00)

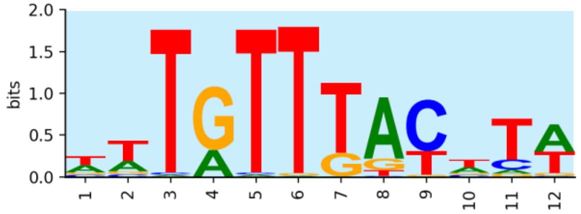

(C) NFE2 (SELEX : M04266_2.00)

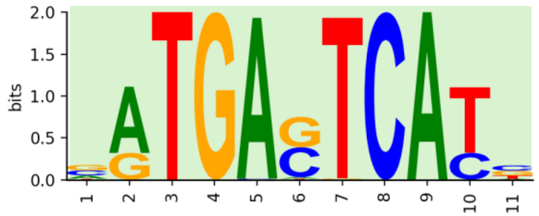

Inferred PWMs from ChIP-seq data for **PBX3**

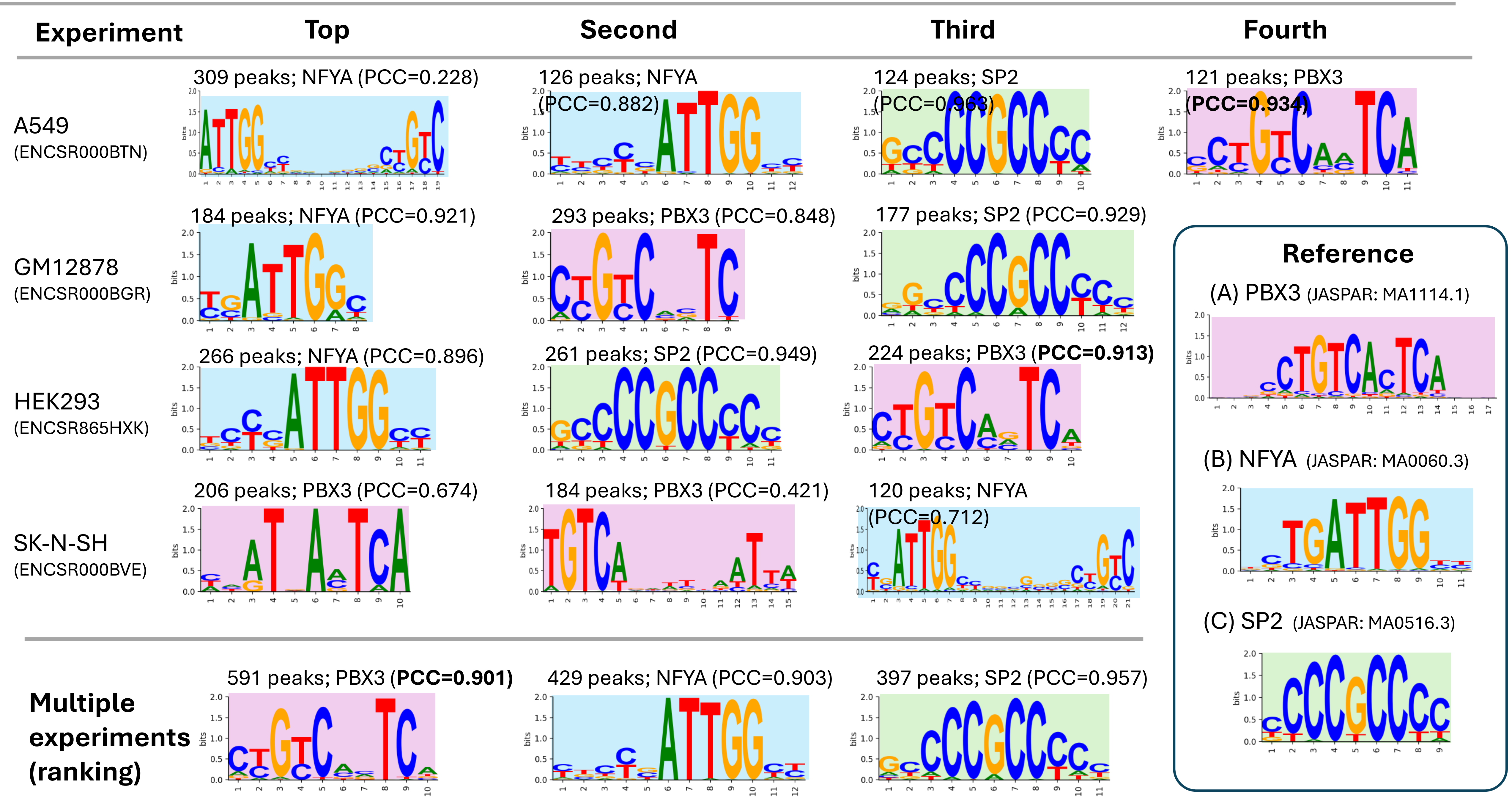

Supplement: S1 File — S1 Fig. Co-binding analysis. Computing the counts of canonical binding, tethered binding and co-binding in a set of ChIP-seq data.S2 Fig. Analysis flowchart. Flow chart detailing the number of TFs with binding motif (PWM) inferred from ENCODE ChIP-seq data (July 2020 – January 2023). S3 Fig. Core motifs. Expanded identification across major TF families. S4 Fig. Ranking method. Identification of canonical PWM for a TF using the ranking approach. (PDF) [file pone.0329226.s001.pdf]
