# Supplementary material for: Positional distribution of transcription factor binding sites in the human genome
Source: PLoS One. 2025 Jul 30;20(7):e0329226. doi: 10.1371/journal.pone.0329226 (PMC12310040; doi:10.1371/journal.pone.0329226)
Supplement: S2 File — S1 Table. Canonical PWMs. Summary of canonical PWMs for TFs and inferred PWMs from individual experiments. S2 Table. Co-binding frequencies. Frequencies of canonical binding, tethered binding and co-binding sites. S3 Table. Co-occurring motifs. Overview of identified motifs that frequently co-occur in binding regions. S4 Table. TFBSs near CTCF-bound sites. Summary of transcription factor binding sites within 200 bp of CTCF-bound regions. (PDF) [file pone.0329226.s002.pdf]

S1 Table. Canonical PWMs of TFs and all inferred PWMs from individual experiments

TF	Motif ID	TF family	Method	Type of Motif
ARNTL	ARNTL_HepG2_ENCSR794LVK_merged_N1	bHLH	experiment	canonical
ATF1	ATF1_representative_N1	bZIP	ranking	canonical
ATF2	ATF2_representative_N1	bZIP	ranking	canonical
ATF3	ATF3_representativeHSAv2_N1	bZIP	ranking	canonical
ATF4	ATF4_representativeHSAv2_N1	bZIP	ranking	canonical
ATF7	ATF7_representative_N1	bZIP	ranking	canonical
BACH1	BACH1_representative_N1	bZIP	ranking	canonical
BATF	BATF_GM12878_ENCSR000BGT_merged_N1	bZIP	experiment	canonical
BCL6	BCL6_representativeHSAv2_N1	C2H2 ZF	ranking	canonical
BCL6B	BCL6B_HEK293_ENCSR673SGK_merged_N1	C2H2 ZF	experiment	canonical
BHLHA15	BHLHA15_HepG2_ENCSR888QFJ_merged_N1	bHLH	experiment	canonical
BHLHE40	BHLHE40_representative_N1	bHLH	ranking	canonical
CEBPA	CEBPA_HepG2_ENCSR142IGM_merged_N1	bZIP	experiment	canonical
CEBPB	CEBPB_representative_N1	bZIP	ranking	canonical
CEBPD	CEBPD_HepG2_ENCSR520MCD_merged_N1	bZIP	experiment	canonical
CEBPG	CEBPG_representative_N1	bZIP	ranking	canonical
CLOCK	CLOCK_MCF-7_ENCSR699YFX_Rep1_N1	bHLH	recovered	canonical
CREB1	CREB1_representative_N1	bZIP	ranking	canonical
CREB3	CREB3_HepG2_ENCSR855XFL_merged_N1	bZIP	experiment	canonical
CREB3L1	CREB3L1_K562_ENCSR109YGM_merged_N1	bZIP	experiment	canonical
CREM	CREM_representative_N1	bZIP	ranking	canonical
CTCF	CTCF_representativeHSAv2_N1	C2H2 ZF	ranking	canonical
CTCFL	CTCFL_K562_ENCSR000BNK_merged_N1	C2H2 ZF	experiment	canonical
CUX1	CUX1_representative_N1	CUT; Homeodomain	ranking	canonical
DDIT3	DDIT3_representativeHSAv2_N1	bZIP	ranking	canonical
E2F5	E2F5_HepG2_ENCSR486JYI_merged_N1	E2F	experiment	canonical
E2F6	E2F6_representative_N1	E2F	ranking	canonical
E2F8	E2F8_representativeHSAv2_N1	E2F	ranking	canonical
EBF1	EBF1_representative_N1	EBF1	ranking	canonical
EGR1	EGR1_representative_N1	C2H2 ZF	ranking	canonical

ELF1	ELF1_representative_N1	Ets	ranking	canonical
ELF2	ELF2_K562_ENCSR594HXD_merged_N1	Ets	experiment	canonical
ELF3	ELF3_HepG2_ENCSR770AOR_merged_N1	Ets; AT hook	experiment	canonical
ELF4	ELF4_representative_N1	Ets	ranking	canonical
ELK1	ELK1_representative_N1	Ets	ranking	canonical
ELK4	ELK4_representativeHSAv2_N1	Ets	ranking	canonical
ESR1	ESR1_representativeHSAv2_N1	Nuclear receptor	ranking	canonical
ESRRA	ESRRA_representativeHSAv2_N1	Nuclear receptor	ranking	canonical
ETS1	ETS1_representative_N1	Ets	ranking	canonical
ETV1	ETV1_K562_ENCSR277DMR_merged_N1	Ets	experiment	canonical
ETV4	ETV4_representativeHSAv2_N1	Ets	ranking	canonical
ETV5	ETV5_representativeHSAv2_N1	Ets	ranking	canonical
ETV6	ETV6_representativeHSAv2_N2	Ets	ranking	canonical
FOS	FOS_representative_N1	bZIP	ranking	canonical
FOSL1	FOSL1_representative_N1	bZIP	ranking	canonical
FOSL2	FOSL2_representativeHSAv2_N1	bZIP	ranking	canonical
FOXA1	FOXA1_representativeHSAv2_N1	Forkhead	ranking	canonical
FOXA2	FOXA2_representative_N1	Forkhead	ranking	canonical
FOXA3	FOXA3_representativeHSAv2_N1	Forkhead	ranking	canonical
FOXF2	FOXF2_A549_ENCSR445FHB_merged_N1	Forkhead	experiment	canonical
FOXJ3	FOXJ3_representativeHSAv2_N1	Forkhead	ranking	canonical
FOXK1	FOXK1_representativeHSAv2_N1	Forkhead	ranking	canonical
FOXK2	FOXK2_representative_N1	Forkhead	ranking	canonical
FOXO1	FOXO1_HepG2_ENCSR321OAA_merged_N1	Forkhead	experiment	canonical
FOXP1	FOXP1_representative_N1	Forkhead	ranking	canonical
FOXP2	FOXP2_PFSK-1_ENCSR000BGA_merged_N1	Forkhead	experiment	canonical
FOXQ1	FOXQ1_HepG2_ENCSR199MFT_merged_N1	Forkhead	experiment	canonical
GABPA	GABPA_representative_N1	Ets	ranking	canonical
GATA1	GATA1_representative_N2	GATA	ranking	canonical
GATA2	GATA2_representative_N1	GATA	ranking	canonical
GATA3	GATA3_representative_N1	GATA	ranking	canonical
GATA4	GATA4_HepG2_ENCSR590CNM_merged_N1	GATA	experiment	canonical

GFI1	GFI1_HepG2_ENCSR849FVL_merged_N1	C2H2 ZF	experiment	canonical
GFI1B	GFI1B_HEK293_ENCSR445PDR_merged_N1	C2H2 ZF	experiment	canonical
GLIS1	GLIS1_HEK293_ENCSR482BBZ_merged_N1	C2H2 ZF	experiment	canonical
GMEB1	GMEB1_representativeHSAv2_N1	SAND	ranking	canonical
GTF2I	GTF2I_representativeHSAv2_N1	GTF2I-like	ranking	canonical
GZF1	GZF1_HepG2_ENCSR479WQX_merged_N2	C2H2 ZF	experiment	canonical
HIC1	HIC1_HEK293_ENCSR803GYT_merged_N1	C2H2 ZF	experiment	canonical
HIC2	HIC2_HepG2_ENCSR015LYB_merged_N1	C2H2 ZF	experiment	canonical
HINFP	HINFP_representativeHSAv2_N2	C2H2 ZF	ranking	canonical
HLF	HLF_HepG2_ENCSR528PSI_merged_N1	bZIP	experiment	canonical
HMBOX1	HMBOX1_K562_ENCSR757IIU_merged_N1	Homeodomain	experiment	canonical
HNF1A	HNF1A_representativeHSAv2_N1	Homeodomain	ranking	canonical
HNF1B	HNF1B_HepG2_ENCSR127XTZ_merged_N1	Homeodomain	experiment	canonical
HNF4A	HNF4A_representative_N1	Nuclear receptor	ranking	canonical
HNF4G	HNF4G_representative_N1	Nuclear receptor	ranking	canonical
HOXD13	HOXD13_HEK293_ENCSR418XXL_merged_N1	Homeodomain	experiment	canonical
HSF2	HSF2_HepG2_ENCSR764ZBK_merged_N1	HSF	experiment	canonical
IKZF1	IKZF1_representative_N1	C2H2 ZF	ranking	canonical
IKZF2	IKZF2_representative_N1	C2H2 ZF	ranking	canonical
IRF1	IRF1_representative_N1	IRF	ranking	canonical
IRF2	IRF2_representative_N1	IRF	ranking	canonical
JUN	JUN_representative_N1	bZIP	ranking	canonical
JUNB	JUNB_representative_N1	bZIP	ranking	canonical
JUND	JUND_representative_N1	bZIP	ranking	canonical
KLF1	KLF1_representative_N1	C2H2 ZF	ranking	canonical
KLF10	KLF10_HEK293_ENCSR006GAQ_merged_N1	C2H2 ZF	experiment	canonical
KLF12	KLF12_HepG2_ENCSR552YWL_merged_N1	C2H2 ZF	experiment	canonical
KLF13	KLF13_K562_ENCSR608HVP_Rep2_N1	C2H2 ZF	recovered	canonical
KLF4	KLF4_MCF-7_ENCSR265WJC_merged_N1	C2H2 ZF	experiment	canonical
KLF8	KLF8_HEK293_ENCSR635NOQ_merged_N1	C2H2 ZF	experiment	canonical
KLF9	KLF9_representative_N1	C2H2 ZF	ranking	canonical
LEF1	LEF1_K562_ENCSR832OGB_merged_N1	HMG/Sox	experiment	canonical

MAFF	MAFF_representative_N1	bZIP	ranking	canonical
MAFG	MAFG_representativeHSAv2_N1	bZIP	ranking	canonical
MAFK	MAFK_representative_N1	bZIP	ranking	canonical
MAX	MAX_representative_N1	bHLH	ranking	canonical
MAZ	MAZ_representative_N1	C2H2 ZF	ranking	canonical
MEF2A	MEF2A_representativeHSAv2_N1	MADS box	ranking	canonical
MEF2D	MEF2D_representativeHSAv2_N1	MADS box	ranking	canonical
MEIS1	MEIS1_HEK293_ENCSR186JMM_Rep2_N1	Homeodomain	recovered	canonical
MEIS2	MEIS2_K562_ENCSR851BNE_merged_N1	Homeodomain	experiment	canonical
MITF	MITF_representative_N1	bHLH	ranking	canonical
MLX	MLX_HepG2_ENCSR125DAD_merged_N1	bHLH	experiment	canonical
MNT	MNT_representative_N1	bHLH	ranking	canonical
MXI1	MXI1_representative_N1	bHLH	ranking	canonical
MYC	MYC_representativeHSAv2_N1	bHLH	ranking	canonical
MZF1	MZF1_HEK293_ENCSR298QUH_merged_N1	C2H2 ZF	experiment	canonical
NFATC1	NFATC1_GM12878_ENCSR000BQL_merged_N1	Rel	experiment	canonical
NFIA	NFIA_HepG2_ENCSR226QQM_merged_N1	SMAD	experiment	canonical
NFIB	NFIB_representative_N1	SMAD	ranking	canonical
NFIC	NFIC_representative_N1	SMAD	ranking	canonical
NFIL3	NFIL3_HepG2_ENCSR201GGK_merged_N1	bZIP	experiment	canonical
NFIX	NFIX_K562_ENCSR574VJG_merged_N1	SMAD	experiment	canonical
NFYA	NFYA_representativeHSAv2_N1	CBF/NF-Y	ranking	canonical
NFYB	NFYB_representativeHSAv2_N1	Unknown	ranking	canonical
NFYC	NFYC_HepG2_ENCSR569ARC_merged_N1	Unknown	experiment	canonical
NR2C1	NR2C1_representative_N2	Nuclear receptor	ranking	canonical
NR2C2	NR2C2_representative_N3	Nuclear receptor	ranking	canonical
NR2F1	NR2F1_representative_N1	Nuclear receptor	ranking	canonical
NR2F2	NR2F2_representative_N1	Nuclear receptor	ranking	canonical
NR2F6	NR2F6_representative_N1	Nuclear receptor	ranking	canonical
NR3C1	NR3C1_representative_N1	Nuclear receptor	ranking	canonical
NR5A1	NR5A1_HepG2_ENCSR310OZS_merged_N1	Nuclear receptor	experiment	canonical
NR5A2	NR5A2_A549_ENCSR190GIW_merged_N1	Nuclear receptor	experiment	canonical

NRF1	NRF1_representative_N1	Unknown	ranking	canonical
ONECUT1	ONECUT1_HepG2_ENCSR956OSX_merged_N1	CUT; Homeodomain	experiment	canonical
ONECUT2	ONECUT2_HepG2_ENCSR661PKJ_merged_N1	CUT; Homeodomain	experiment	canonical
OTX2	OTX2_WTC11_ENCSR004HEA_merged_N1	Homeodomain	experiment	canonical
OVOL1	OVOL1_MCF-7_ENCSR829WBA_merged_N1	C2H2 ZF	experiment	canonical
PATZ1	PATZ1_representativeHSAv2_N1	C2H2 ZF; AT hook	ranking	canonical
PAX5	PAX5_representative_N1	Paired box	ranking	canonical
PBX3	PBX3_representativeHSAv2_N1	Homeodomain	ranking	canonical
PKNOX1	PKNOX1_representative_N2	Homeodomain	ranking	canonical
POU2F2	POU2F2_representative_N1	Homeodomain; POU	ranking	canonical
POU5F1	POU5F1_GM23338_ENCSR264RJX_merged_N1	Homeodomain; POU	experiment	canonical
PRDM1	PRDM1_representativeHSAv2_N1	C2H2 ZF	ranking	canonical
PRDM15	PRDM15_representativeHSAv2_N1	C2H2 ZF	ranking	canonical
PRDM4	PRDM4_representativeHSAv2_N1	C2H2 ZF	ranking	canonical
RARA	RARA_HepG2_ENCSR500WXT_merged_N1	Nuclear receptor	experiment	canonical
RBAK	RBAK_HepG2_ENCSR144NTH_merged_N1	C2H2 ZF	experiment	canonical
RBPJ	RBPJ_HepG2_ENCSR596FEL_merged_N1	CSL	experiment	canonical
RELA	RELA_representativeHSAv2_N1	Rel	ranking	canonical
REST	REST_representativeHSAv2_N1	C2H2 ZF	ranking	canonical
RFX1	RFX1_MCF-7_ENCSR788XNX_merged_N1	RFX	experiment	canonical
RFX3	RFX3_HepG2_ENCSR633OVO_merged_N1	RFX	experiment	canonical
RFX5	RFX5_representative_N2	RFX	ranking	canonical
RUNX1	RUNX1_K562_ENCSR588AKU_merged_N2	Runt	experiment	canonical
RUNX3	RUNX3_GM12878_ENCSR000BRI_merged_N1	Runt	experiment	canonical
RXRA	RXRA_representativeHSAv2_N1	Nuclear receptor	ranking	canonical
RXRB	RXRB_HepG2_ENCSR560SEP_merged_N1	Nuclear receptor	experiment	canonical
SCRT1	SCRT1_HEK293_ENCSR605MGM_merged_N1	C2H2 ZF	experiment	canonical
SCRT2	SCRT2_HEK293_ENCSR338DGO_merged_N1	C2H2 ZF	experiment	canonical
SMAD1	SMAD1_GM12878_ENCSR813DCK_merged_N1	SMAD	experiment	canonical
SOX13	SOX13_representative_N1	HMG/Sox	ranking	canonical
SOX5	SOX5_HepG2_ENCSR961WLZ_merged_N1	HMG/Sox	experiment	canonical
SOX6	SOX6_representativeHSAv2_N1	HMG/Sox	ranking	canonical

SP1	SP1_representative_N3	C2H2 ZF	ranking	canonical
SP2	SP2_representative_N1	C2H2 ZF	ranking	canonical
SP3	SP3_HEK293_ENCSR141PZA_merged_N1	C2H2 ZF	experiment	canonical
SP4	SP4_H1-hESC_ENCSR000BQV_merged_N1	C2H2 ZF	experiment	canonical
SP5	SP5_HepG2_ENCSR019NPF_merged_N1	C2H2 ZF	experiment	canonical
SPDEF	SPDEF_MCF-7_ENCSR042GSX_merged_N1	Ets	experiment	canonical
SPI1	SPI1_representative_N1	Ets	ranking	canonical
STAT1	STAT1_HeLa-S3_ENCSR000EZK_merged_N1	STAT	experiment	canonical
STAT3	STAT3_representative_N1	STAT	ranking	canonical
TAL1	TAL1_K562_ENCSR106FRG_merged_N3	bHLH	experiment	canonical
TBP	TBP_representative_N1	TBP	ranking	canonical
TBX21	TBX21_GM12878_ENCSR739IHN_merged_N1	T-box	experiment	canonical
TBX3	TBX3_HepG2_ENCSR238QRG_merged_N2	T-box	experiment	canonical
TCF12	TCF12_H1_ENCSR000BIT_merged_N1	bHLH	experiment	canonical
TCF7	TCF7_representative_N1	HMG/Sox	ranking	canonical
TCF7L2	TCF7L2_representative_N1	HMG/Sox	ranking	canonical
TEAD1	TEAD1_K562_ENCSR228ELU_Rep2_N1	TEA	recovered	canonical
TEAD3	TEAD3_HepG2_ENCSR666QNP_merged_N1	TEA	experiment	canonical
TEAD4	TEAD4_representativeHSAv2_N1	TEA	ranking	canonical
TEF	TEF_HepG2_ENCSR583KLD_merged_N1	bZIP	experiment	canonical
TFAP4	TFAP4_representativeHSAv2_N1	bHLH	ranking	canonical
TFCP2	TFCP2_K562_ENCSR998OMC_merged_N1	Grainyhead	experiment	canonical
TFDP1	TFDP1_representative_N1	E2F	ranking	canonical
TFDP2	TFDP2_HepG2_ENCSR069JKP_merged_N1	E2F	experiment	canonical
TFE3	TFE3_representativeHSAv2_N1	bHLH	ranking	canonical
TGIF2	TGIF2_representativeHSAv2_N1	Homeodomain	ranking	canonical
THRA	THRA_representativeHSAv2_N1	Nuclear receptor	ranking	canonical
THRB	THRB_representativeHSAv2_N1	Nuclear receptor	ranking	canonical
TP53	TP53_HepG2_ENCSR980EGJ_merged_N1	p53	experiment	canonical
USF1	USF1_representativeHSAv2_N1	bHLH	ranking	canonical
USF2	USF2_representativeHSAv2_N1	bHLH	ranking	canonical
VEZF1	VEZF1_K562_ENCSR189YMA_merged_N1	C2H2 ZF	experiment	canonical

YY1	YY1_representative_N1	C2H2 ZF	ranking	canonical
YY2	YY2_HEK293_ENCSR692HSE_merged_N1	C2H2 ZF	experiment	canonical
ZBTB11	ZBTB11_representative_N1	C2H2 ZF	ranking	canonical
ZBTB26	ZBTB26_HepG2_ENCSR184SVO_merged_N1	C2H2 ZF	experiment	canonical
ZBTB33	ZBTB33_representativeHSAv2_N1	C2H2 ZF	ranking	canonical
ZBTB42	ZBTB42_HepG2_ENCSR232AAR_merged_N1	C2H2 ZF	experiment	canonical
ZBTB44	ZBTB44_HEK293_ENCSR076STQ_merged_N1	C2H2 ZF	experiment	canonical
ZBTB48	ZBTB48_HEK293_ENCSR781EQJ_merged_N1	C2H2 ZF	experiment	canonical
ZEB1	ZEB1_representative_N1	C2H2 ZF; Homeodomain	ranking	canonical
ZEB2	ZEB2_representative_N1	C2H2 ZF; Homeodomain	ranking	canonical
ZFP1	ZFP1_HepG2_ENCSR586DEH_merged_N1	C2H2 ZF	experiment	canonical
ZFP14	ZFP14_HepG2_ENCSR620DBD_merged_N1	C2H2 ZF	experiment	canonical
ZFP90	ZFP90_HepG2_ENCSR377GZS_merged_N3	C2H2 ZF	experiment	canonical
ZFX	ZFX_representative_N1	C2H2 ZF	ranking	canonical
ZIC2	ZIC2_HEK293_ENCSR728MWW_merged_N1	C2H2 ZF	experiment	canonical
ZKSCAN1	ZKSCAN1_representative_N1	C2H2 ZF	ranking	canonical
ZKSCAN3	ZKSCAN3_K562_ENCSR199HGP_merged_N1	C2H2 ZF	experiment	canonical
ZKSCAN5	ZKSCAN5_HepG2_ENCSR159BTO_merged_N2	C2H2 ZF	experiment	canonical
ZNF10	ZNF10_HEK293_ENCSR019WUS_merged_N1	C2H2 ZF	experiment	canonical
ZNF121	ZNF121_representativeHSAv2_N1	C2H2 ZF	ranking	canonical
ZNF124	ZNF124_HepG2_ENCSR357YPP_merged_N1	C2H2 ZF	experiment	canonical
ZNF134	ZNF134_K562_ENCSR553NTC_merged_N1	C2H2 ZF	experiment	canonical
ZNF140	ZNF140_HEK293_ENCSR464KFG_Rep2_N1	C2H2 ZF	recovered	canonical
ZNF146	ZNF146_K562_ENCSR713IFY_merged_N1	C2H2 ZF	experiment	canonical
ZNF148	ZNF148_K562_ENCSR018MSO_merged_N2	C2H2 ZF	experiment	canonical
ZNF157	ZNF157_HEK293_ENCSR564YYW_merged_N1	C2H2 ZF	experiment	canonical
ZNF18	ZNF18_HEK293_ENCSR977HTH_merged_N1	C2H2 ZF	experiment	canonical
ZNF189	ZNF189_HEK293_ENCSR163RYW_merged_N1	C2H2 ZF	experiment	canonical
ZNF197	ZNF197_K562_ENCSR580IAO_merged_N1	C2H2 ZF	experiment	canonical
ZNF232	ZNF232_HepG2_ENCSR837GLU_merged_N2	C2H2 ZF	experiment	canonical
ZNF24	ZNF24_representative_N1	C2H2 ZF	ranking	canonical
ZNF25	ZNF25_HepG2_ENCSR384SQB_merged_N2	C2H2 ZF	experiment	canonical

ZNF26	ZNF26_HEK293_ENCSR028EGI_merged_N1	C2H2 ZF	experiment	canonical
ZNF263	ZNF263_representativeHSAv2_N2	C2H2 ZF	ranking	canonical
ZNF264	ZNF264_HepG2_ENCSR248BVU_merged_N2	C2H2 ZF	experiment	canonical
ZNF274	ZNF274_H1-hESC_ENCSR000EUN_merged_N1	C2H2 ZF	experiment	canonical
ZNF281	ZNF281_K562_ENCSR214EKV_merged_N1	C2H2 ZF	experiment	canonical
ZNF329	ZNF329_HepG2_ENCSR681KXT_merged_N1	C2H2 ZF	experiment	canonical
ZNF337	ZNF337_HepG2_ENCSR759KXQ_merged_N1	C2H2 ZF	experiment	canonical
ZNF33B	ZNF33B_HepG2_ENCSR052FXA_merged_N1	C2H2 ZF	experiment	canonical
ZNF341	ZNF341_HEK293_ENCSR185FOY_merged_N2	C2H2 ZF	experiment	canonical
ZNF354B	ZNF354B_K562_ENCSR674SCQ_merged_N2	C2H2 ZF	experiment	canonical
ZNF384	ZNF384_representative_N1	C2H2 ZF	ranking	canonical
ZNF398	ZNF398_HEK293_ENCSR676ZEF_merged_N1	C2H2 ZF	experiment	canonical
ZNF430	ZNF430_HepG2_ENCSR618END_merged_N1	C2H2 ZF	experiment	canonical
ZNF431	ZNF431_representativeHSAv2_N2	C2H2 ZF	ranking	canonical
ZNF436	ZNF436_K562_ENCSR335SUD_merged_N1	C2H2 ZF	experiment	canonical
ZNF444	ZNF444_representativeHSAv2_N3	C2H2 ZF	ranking	canonical
ZNF445	ZNF445_K562_ENCSR883UGG_merged_N1	C2H2 ZF	experiment	canonical
ZNF449	ZNF449_representativeHSAv2_N1	C2H2 ZF	ranking	canonical
ZNF460	ZNF460_HepG2_ENCSR261UIH_merged_N2	C2H2 ZF	experiment	canonical
ZNF483	ZNF483_HepG2_ENCSR436PIH_merged_N1	C2H2 ZF	experiment	canonical
ZNF490	ZNF490_HepG2_ENCSR542PZA_merged_N1	C2H2 ZF	experiment	canonical
ZNF524	ZNF524_HEK293_ENCSR418NZA_merged_N1	C2H2 ZF; AT hook	experiment	canonical
ZNF549	ZNF549_HEK293_ENCSR185QFX_merged_N1	C2H2 ZF	experiment	canonical
ZNF561	ZNF561_HEK293_ENCSR125ULS_merged_N1	C2H2 ZF	experiment	canonical
ZNF562	ZNF562_HepG2_ENCSR727IJD_merged_N1	C2H2 ZF	experiment	canonical
ZNF574	ZNF574_representativeHSAv2_N3	C2H2 ZF	ranking	canonical
ZNF589	ZNF589_HepG2_ENCSR827ZGB_merged_N1	C2H2 ZF	experiment	canonical
ZNF652	ZNF652_HepG2_ENCSR502GAX_merged_N1	C2H2 ZF	experiment	canonical
ZNF660	ZNF660_HEK293_ENCSR283DOU_merged_N2	C2H2 ZF	experiment	canonical
ZNF680	ZNF680_HEK293_ENCSR307CKC_merged_N1	C2H2 ZF	experiment	canonical
ZNF692	ZNF692_HEK293_ENCSR418MKG_merged_N1	C2H2 ZF	experiment	canonical
ZNF707	ZNF707_representativeHSAv2_N1	C2H2 ZF	ranking	canonical

ZNF740	ZNF740_representativeHSAv2_N1	C2H2 ZF	ranking	canonical
ZNF75A	ZNF75A_K562_ENCSR099MNR_merged_N1	C2H2 ZF	experiment	canonical
ZNF761	ZNF761_HepG2_ENCSR622AMZ_merged_N1	C2H2 ZF	experiment	canonical
ZNF764	ZNF764_K562_ENCSR023OOE_merged_N1	C2H2 ZF	experiment	canonical
ZNF766	ZNF766_K562_ENCSR194IJN_merged_N1	C2H2 ZF	experiment	canonical
ZNF777	ZNF777_HepG2_ENCSR068ZQR_merged_N1	C2H2 ZF	experiment	canonical
ZNF790	ZNF790_HepG2_ENCSR117XJA_merged_N1	C2H2 ZF	experiment	canonical
ZNF792	ZNF792_HepG2_ENCSR396SOH_merged_N1	C2H2 ZF	experiment	canonical
ZSCAN22	ZSCAN22_HepG2_ENCSR050KWL_merged_N1	C2H2 ZF	experiment	canonical
ZSCAN29	ZSCAN29_representativeHSAv2_N1	C2H2 ZF	ranking	canonical
ZSCAN4	ZSCAN4_HEK293_ENCSR211GNP_merged_N1	C2H2 ZF	experiment	canonical
NFE2L1	NFE2L1_K562_ENCSR632SHZ_merged_N1	bZIP	experiment	canonical
NFE2L2	NFE2L2_representative_N1	bZIP	ranking	canonical
SIX1	SIX1_HepG2_ENCSR561BQM_merged_N1	Homeodomain	experiment	canonical
SIX4	SIX4_representativeHSAv2_N1	Homeodomain	ranking	canonical
SRF	SRF_representative_N1	MADS box	ranking	canonical
WT1	WT1_HEK293_ENCSR966PJJ_merged_N1	C2H2 ZF	experiment	canonical
ZBTB14	ZBTB14_HepG2_ENCSR532WFC_merged_N1	C2H2 ZF	experiment	canonical
ADNP	ADNP_K562_ENCSR440VKE_merged_N1	Homeodomain	experiment	candidate canonical
AHDC1	AHDC1_HepG2_ENCSR168AUX_merged_N1	AT hook	experiment	candidate canonical
AR	AR_WTC11_ENCSR762LIP_merged_N1	Nuclear receptor	experiment	candidate canonical
ARNT	ARNT_GM12878_ENCSR590KEQ_merged_N1	bHLH	experiment	candidate canonical
ATF5	ATF5_HepG2_ENCSR887TWV_merged_N1	bZIP	experiment	candidate canonical
ATF6	ATF6_HepG2_ENCSR769CWW_merged_N1	bZIP	experiment	candidate canonical
ATOH8	ATOH8_A549_ENCSR161CZA_merged_N1	bHLH	experiment	candidate canonical
BCL11A	BCL11A_H1-hESC_ENCSR000BMJ_Rep1_N1	C2H2 ZF	recovered	candidate canonical
BCL11B	BCL11B_HEK293_ENCSR770PQN_merged_N1	C2H2 ZF	experiment	candidate canonical
CDC5L	CDC5L_K562_ENCSR121PFY_merged_N1	Myb/SANT	experiment	candidate canonical
CHAMP1	CHAMP1_K562_ENCSR065XVO_merged_N1	C2H2 ZF	experiment	candidate canonical
CREB5	CREB5_K562_ENCSR935PEA_merged_N1	bZIP	experiment	candidate canonical
DACH1	DACH1_K562_ENCSR030TJP_merged_N1	Unknown	experiment	candidate canonical
DLX6	DLX6_HepG2_ENCSR272TOJ_merged_N1	Homeodomain	experiment	candidate canonical

DMBX1	DMBX1_K562_ENCSR091IQZ_merged_N1	Homeodomain	experiment	candidate canonical
DMTF1	DMTF1_representativeHSAv2_N1	Myb/SANT	ranking	candidate canonical
DRAP1	DRAP1_HepG2_ENCSR765MKZ_merged_N3	Unknown	experiment	candidate canonical
DZIP1	DZIP1_HepG2_ENCSR895KNN_merged_N1	C2H2 ZF	experiment	candidate canonical
E2F1	E2F1_representative_N1	E2F	ranking	candidate canonical
E2F3	E2F3_K562_ENCSR036QIR_merged_N1	E2F	experiment	candidate canonical
E2F4	E2F4_representativeHSAv2_N4	E2F	ranking	candidate canonical
E4F1	E4F1_representativeHSAv2_N1	C2H2 ZF	ranking	candidate canonical
EEA1	EEA1_HepG2_ENCSR387TUH_merged_N1	C2H2 ZF	experiment	candidate canonical
EGR2	EGR2_HEK293_ENCSR919CZU_merged_N1	C2H2 ZF	experiment	candidate canonical
EMX1	EMX1_WTC11_ENCSR440UPD_merged_N1	Homeodomain	experiment	candidate canonical
ERF	ERF_HepG2_ENCSR159DQO_merged_N1	Ets	experiment	candidate canonical
ERG	ERG_WTC11_ENCSR127NBZ_merged_N1	Ets	experiment	candidate canonical
FEZF1	FEZF1_HEK293_ENCSR827NWO_merged_N1	C2H2 ZF	experiment	candidate canonical
FOXM1	FOXM1_GM12878_ENCSR000BRU_merged_N1	Forkhead	experiment	candidate canonical
FOXP4	FOXP4_representativeHSAv2_N1	Forkhead	ranking	candidate canonical
FOXS1	FOXS1_A549_ENCSR326AQV_merged_N1	Forkhead	experiment	candidate canonical
GATAD2A	GATAD2A_HepG2_ENCSR925BFV_merged_N1	GATA	experiment	candidate canonical
GMEB2	GMEB2_HepG2_ENCSR745VSQ_merged_N1	SAND	experiment	candidate canonical
HEYL	HEYL_A549_ENCSR995CFS_merged_N1	bHLH	experiment	candidate canonical
HHEX	HHEX_HepG2_ENCSR656JZL_merged_N2	Homeodomain	experiment	candidate canonical
HIVEP1	HIVEP1_representativeHSAv2_N2	C2H2 ZF	ranking	candidate canonical
HMG20A	HMG20A_HepG2_ENCSR072GJV_merged_N3	HMG/Sox	experiment	candidate canonical
HMGA2	HMGA2_WTC11_ENCSR742DAU_merged_N1	AT hook	experiment	candidate canonical
HOMEZ	HOMEZ_HepG2_ENCSR117CHD_merged_N1	Homeodomain	experiment	candidate canonical
HOXA10	HOXA10_HepG2_ENCSR692RKS_merged_N1	Homeodomain	experiment	candidate canonical
HOXA5	HOXA5_HepG2_ENCSR869JZW_merged_N2	Homeodomain	experiment	candidate canonical
HOXB13	HOXB13_A549_ENCSR967ZMR_merged_N1	Homeodomain	experiment	candidate canonical
HOXB5	HOXB5_A549_ENCSR748HJZ_merged_N1	Homeodomain	experiment	candidate canonical
HOXD1	HOXD1_HepG2_ENCSR359TWG_merged_N1	Homeodomain	experiment	candidate canonical
IKZF3	IKZF3_HEK293_ENCSR304AMN_merged_N2	C2H2 ZF	experiment	candidate canonical
IRF4	IRF4_GM12878_ENCSR000BGY_merged_N2	IRF	experiment	candidate canonical

IRF9	IRF9_K562_ENCSR926KTP_merged_N2	IRF	experiment	candidate canonical
KAT7	KAT7_HepG2_ENCSR340BXT_merged_N1	C2H2 ZF	experiment	candidate canonical
KLF11	KLF11_HepG2_ENCSR616OSG_merged_N1	C2H2 ZF	experiment	candidate canonical
KLF16	KLF16_representative_N2	C2H2 ZF	ranking	candidate canonical
KLF17	KLF17_HEK293_ENCSR065WUF_merged_N1	C2H2 ZF	experiment	candidate canonical
KLF6	KLF6_representativeHSAv2_N1	C2H2 ZF	ranking	candidate canonical
LCOR	LCOR_HepG2_ENCSR469GZL_merged_N2	Pipsqueak	experiment	candidate canonical
LCORL	LCORL_HepG2_ENCSR950NAZ_merged_N1	Pipsqueak	experiment	candidate canonical
MEF2B	MEF2B_GM12878_ENCSR177VFS_merged_N2	MADS box	experiment	candidate canonical
MGA	MGA_K562_ENCSR710WLO_merged_N1	T-box	experiment	candidate canonical
MIXL1	MIXL1_HepG2_ENCSR966PJY_merged_N2	Homeodomain	experiment	candidate canonical
MXD1	MXD1_HepG2_ENCSR165YVX_merged_N1	bHLH	experiment	candidate canonical
MXD3	MXD3_HepG2_ENCSR909GJR_merged_N1	bHLH	experiment	candidate canonical
MXD4	MXD4_HepG2_ENCSR441KFW_merged_N1	bHLH	experiment	candidate canonical
MYBL2	MYBL2_representative_N1	Myb/SANT	ranking	candidate canonical
MYNN	MYNN_representative_N1	C2H2 ZF	ranking	candidate canonical
NANOG	NANOG_representative_N1	Homeodomain	ranking	candidate canonical
NCOA3	NCOA3_MCF-7_ENCSR573OJP_merged_N2	bHLH	experiment	candidate canonical
NEUROD1	NEUROD1_K562_ENCSR986CDX_merged_N3	bHLH	experiment	candidate canonical
NFATC3	NFATC3_GM12878_ENCSR437GBJ_merged_N1	Rel	experiment	candidate canonical
NFE2	NFE2_representative_N1	bZIP	ranking	candidate canonical
NFXL1	NFXL1_representativeHSAv2_N1	NFX	ranking	candidate canonical
NKX3-1	NKX3-1_HepG2_ENCSR647CXR_merged_N3	Homeodomain	experiment	candidate canonical
NR4A1	NR4A1_K562_ENCSR130PDE_merged_N1	Nuclear receptor	experiment	candidate canonical
OSR2	OSR2_HEK293_ENCSR324LTM_merged_N1	C2H2 ZF	experiment	candidate canonical
PBX1	PBX1_A549_ENCSR637RKG_merged_N3	Homeodomain	experiment	candidate canonical
PBX2	PBX2_representative_N2	Homeodomain	ranking	candidate canonical
PITX1	PITX1_HepG2_ENCSR374MAS_merged_N1	Homeodomain	experiment	candidate canonical
PPARG	PPARG_HepG2_ENCSR130VQL_merged_N1	Nuclear receptor	experiment	candidate canonical
PRDM10	PRDM10_representative_N1	C2H2 ZF	ranking	candidate canonical
RELB	RELB_GM12878_ENCSR387QUV_merged_N1	Rel	experiment	candidate canonical
RREB1	RREB1_representativeHSAv2_N1	C2H2 ZF	ranking	candidate canonical

SALL1	SALL1_HepG2_ENCSR407MQT_merged_N1	C2H2 ZF	experiment	candidate canonical
SATB2	SATB2_HepG2_ENCSR753KZY_merged_N3	CUT; Homeodomain	experiment	candidate canonical
SETDB1	SETDB1_HEK293_ENCSR348AGV_merged_N1	MBD	experiment	candidate canonical
SHOX2	SHOX2_K562_ENCSR184IQF_merged_N1	Homeodomain	experiment	candidate canonical
SIX5	SIX5_representative_N1	Homeodomain	ranking	candidate canonical
SKI	SKI_HepG2_ENCSR754MUD_merged_N2	Unknown	experiment	candidate canonical
SMAD3	SMAD3_K562_ENCSR376XAV_merged_N4	SMAD	experiment	candidate canonical
SMAD5	SMAD5_K562_ENCSR000FCD_merged_N2	SMAD	experiment	candidate canonical
SNAPC4	SNAPC4_HepG2_ENCSR306HAG_merged_N1	Myb/SANT	experiment	candidate canonical
SP140L	SP140L_HepG2_ENCSR107DKT_merged_N1	SAND	experiment	candidate canonical
STAT5A	STAT5A_K562_ENCSR000BRR_merged_N2	STAT	experiment	candidate canonical
TBX18	TBX18_K562_ENCSR385IUC_merged_N1	T-box	experiment	candidate canonical
TCF3	TCF3_representativeHSAv2_N1	bHLH	ranking	candidate canonical
TCFL5	TCFL5_K562_ENCSR844JVU_merged_N1	bHLH	experiment	candidate canonical
TEAD2	TEAD2_K562_ENCSR635GTR_merged_N1	TEA	experiment	candidate canonical
THAP1	THAP1_K562_ENCSR000BNN_merged_N1	THAP finger	experiment	candidate canonical
THAP12	THAP12_K562_ENCSR415NNQ_merged_N1	THAP finger	experiment	candidate canonical
THAP9	THAP9_HepG2_ENCSR123GPC_merged_N1	THAP finger	experiment	candidate canonical
TRERF1	TRERF1_WTC11_ENCSR547WSB_merged_N1	C2H2 ZF; Myb/SANT	experiment	candidate canonical
TSHZ1	TSHZ1_K562_ENCSR958JPH_merged_N1	C2H2 ZF	experiment	candidate canonical
YBX1	YBX1_HepG2_ENCSR799GOY_merged_N1	CSD	experiment	candidate canonical
ZBED4	ZBED4_HepG2_ENCSR409PMR_merged_N2	BED ZF	experiment	candidate canonical
ZBED5	ZBED5_HepG2_ENCSR656SIB_merged_N1	BED ZF	experiment	candidate canonical
ZBTB10	ZBTB10_representativeHSAv2_N1	C2H2 ZF	ranking	candidate canonical
ZBTB17	ZBTB17_representativeHSAv2_N2	C2H2 ZF	ranking	candidate canonical
ZBTB2	ZBTB2_representativeHSAv2_N2	C2H2 ZF	ranking	candidate canonical
ZBTB20	ZBTB20_HEK293_ENCSR460MBI_merged_N1	C2H2 ZF	experiment	candidate canonical
ZBTB21	ZBTB21_HepG2_ENCSR549PAU_merged_N3	C2H2 ZF	experiment	candidate canonical
ZBTB24	ZBTB24_HepG2_ENCSR396XDF_merged_N1	C2H2 ZF; AT hook	experiment	candidate canonical
ZBTB3	ZBTB3_HepG2_ENCSR481AIK_merged_N2	C2H2 ZF	experiment	candidate canonical
ZBTB34	ZBTB34_K562_ENCSR567QAD_merged_N1	C2H2 ZF	experiment	candidate canonical
ZBTB40	ZBTB40_representativeHSAv2_N2	C2H2 ZF	ranking	candidate canonical

ZBTB43	ZBTB43_HepG2_ENCSR173USN_merged_N3	C2H2 ZF	experiment	candidate canonical
ZBTB49	ZBTB49_HepG2_ENCSR803IYP_merged_N1	C2H2 ZF	experiment	candidate canonical
ZBTB5	ZBTB5_K562_ENCSR967AVR_merged_N1	C2H2 ZF	experiment	candidate canonical
ZBTB6	ZBTB6_HEK293_ENCSR619OUC_merged_N1	C2H2 ZF	experiment	candidate canonical
ZBTB7A	ZBTB7A_representative_N1	C2H2 ZF	ranking	candidate canonical
ZBTB7B	ZBTB7B_MCF-7_ENCSR277BXW_merged_N2	C2H2 ZF	experiment	candidate canonical
ZBTB8A	ZBTB8A_HEK293_ENCSR481FEC_merged_N1	C2H2 ZF	experiment	candidate canonical
ZBTB9	ZBTB9_K562_ENCSR536CBU_merged_N1	C2H2 ZF	experiment	candidate canonical
ZFP3	ZFP3_HEK293_ENCSR134QIE_merged_N1	C2H2 ZF	experiment	candidate canonical
ZFP37	ZFP37_HEK293_ENCSR365GRX_merged_N1	C2H2 ZF	experiment	candidate canonical
ZFP64	ZFP64_representative_N1	C2H2 ZF	ranking	candidate canonical
ZFP69B	ZFP69B_HEK293_ENCSR381VYR_merged_N1	C2H2 ZF	experiment	candidate canonical
ZFP91	ZFP91_representativeHSAv2_N1	C2H2 ZF	ranking	candidate canonical
ZFY	ZFY_HepG2_ENCSR949OEV_merged_N1	C2H2 ZF	experiment	candidate canonical
ZGPAT	ZGPAT_HepG2_ENCSR704IGU_merged_N2	CCCH ZF	experiment	candidate canonical
ZHX1	ZHX1_HepG2_ENCSR978DQT_merged_N2	Homeodomain	experiment	candidate canonical
ZKSCAN8	ZKSCAN8_HepG2_ENCSR808FFI_merged_N1	C2H2 ZF	experiment	candidate canonical
ZNF101	ZNF101_HEK293_ENCSR462FWS_merged_N1	C2H2 ZF	experiment	candidate canonical
ZNF133	ZNF133_HEK293_ENCSR283MWQ_merged_N1	C2H2 ZF	experiment	candidate canonical
ZNF138	ZNF138_HepG2_ENCSR910MUD_merged_N1	C2H2 ZF	experiment	candidate canonical
ZNF142	ZNF142_HepG2_ENCSR512ECF_merged_N1	C2H2 ZF	experiment	candidate canonical
ZNF143	ZNF143_representative_N1	C2H2 ZF	ranking	candidate canonical
ZNF160	ZNF160_HepG2_ENCSR917SSX_Rep1_N4	C2H2 ZF	recovered	candidate canonical
ZNF165	ZNF165_K562_ENCSR172XJS_merged_N1	C2H2 ZF	experiment	candidate canonical
ZNF175	ZNF175_K562_ENCSR011PEI_merged_N1	C2H2 ZF	experiment	candidate canonical
ZNF184	ZNF184_representative_N1	C2H2 ZF	ranking	candidate canonical
ZNF20	ZNF20_HepG2_ENCSR720PDY_merged_N1	C2H2 ZF	experiment	candidate canonical
ZNF205	ZNF205_HepG2_ENCSR157CEN_merged_N1	C2H2 ZF	experiment	candidate canonical
ZNF215	ZNF215_K562_ENCSR699RWG_merged_N1	C2H2 ZF	experiment	candidate canonical
ZNF217	ZNF217_HepG2_ENCSR055FQB_merged_N3	C2H2 ZF	experiment	candidate canonical
ZNF23	ZNF23_K562_ENCSR898IDK_merged_N2	C2H2 ZF	experiment	candidate canonical
ZNF239	ZNF239_representativeHSAv2_N1	C2H2 ZF	ranking	candidate canonical

ZNF250	ZNF250_K562_ENCSR630KQP_merged_N1	C2H2 ZF	experiment	candidate canonical
ZNF257	ZNF257_K562_ENCSR492FKD_merged_N1	C2H2 ZF	experiment	candidate canonical
ZNF3	ZNF3_HepG2_ENCSR182QWU_merged_N1	C2H2 ZF	experiment	candidate canonical
ZNF302	ZNF302_A549_ENCSR590IHT_merged_N1	C2H2 ZF	experiment	candidate canonical
ZNF311	ZNF311_K562_ENCSR388ZRV_Rep1_N1	C2H2 ZF	recovered	candidate canonical
ZNF316	ZNF316_representative_N1	C2H2 ZF	ranking	candidate canonical
ZNF318	ZNF318_HepG2_ENCSR832PID_merged_N1	C2H2 ZF	experiment	candidate canonical
ZNF319	ZNF319_K562_ENCSR231PDA_merged_N4	C2H2 ZF	experiment	candidate canonical
ZNF324	ZNF324_K562_ENCSR712KVZ_merged_N1	C2H2 ZF	experiment	candidate canonical
ZNF331	ZNF331_HepG2_ENCSR369TCR_merged_N1	C2H2 ZF	experiment	candidate canonical
ZNF34	ZNF34_HEK293_ENCSR727PIC_merged_N1	C2H2 ZF	experiment	candidate canonical
ZNF354C	ZNF354C_K562_ENCSR506SSQ_Rep1_N1	C2H2 ZF	recovered	candidate canonical
ZNF362	ZNF362_representativeHSAv2_N1	C2H2 ZF	ranking	candidate canonical
ZNF366	ZNF366_HEK293_ENCSR106EBH_merged_N1	C2H2 ZF	experiment	candidate canonical
ZNF394	ZNF394_HEK293_ENCSR125DNC_merged_N3	C2H2 ZF	experiment	candidate canonical
ZNF397	ZNF397_K562_ENCSR508EEX_merged_N1	C2H2 ZF	experiment	candidate canonical
ZNF407	ZNF407_representative_N1	C2H2 ZF	ranking	candidate canonical
ZNF408	ZNF408_K562_ENCSR758PLU_merged_N1	C2H2 ZF	experiment	candidate canonical
ZNF41	ZNF41_K562_ENCSR235PYI_merged_N1	C2H2 ZF	experiment	candidate canonical
ZNF423	ZNF423_WTC11_ENCSR413CVQ_merged_N1	C2H2 ZF	experiment	candidate canonical
ZNF426	ZNF426_HEK293_ENCSR224NFP_merged_N1	C2H2 ZF	experiment	candidate canonical
ZNF48	ZNF48_representative_N1	C2H2 ZF	ranking	candidate canonical
ZNF503	ZNF503_HepG2_ENCSR998YJI_merged_N3	C2H2 ZF	experiment	candidate canonical
ZNF511	ZNF511_representativeHSAv2_N1	C2H2 ZF	ranking	candidate canonical
ZNF512	ZNF512_K562_ENCSR591CCL_merged_N1	C2H2 ZF; BED ZF	experiment	candidate canonical
ZNF513	ZNF513_HEK293_ENCSR503DPC_merged_N1	C2H2 ZF	experiment	candidate canonical
ZNF518A	ZNF518A_HEK293_ENCSR159GFL_merged_N2	C2H2 ZF	experiment	candidate canonical
ZNF558	ZNF558_representativeHSAv2_N1	C2H2 ZF	ranking	candidate canonical
ZNF576	ZNF576_HepG2_ENCSR253HUM_merged_N1	C2H2 ZF	experiment	candidate canonical
ZNF577	ZNF577_HEK293_ENCSR776MDR_Rep2_N1	C2H2 ZF	recovered	candidate canonical
ZNF579	ZNF579_MCF-7_ENCSR018MQH_merged_N1	C2H2 ZF	experiment	candidate canonical
ZNF580	ZNF580_representative_N1	C2H2 ZF	ranking	candidate canonical

ZNF583	ZNF583_K562_ENCSR775EQV_merged_N1	C2H2 ZF	experiment	candidate canonical
ZNF584	ZNF584_K562_ENCSR149ZBI_merged_N1	C2H2 ZF	experiment	candidate canonical
ZNF585B	ZNF585B_HEK293_ENCSR011XCI_merged_N1	C2H2 ZF	experiment	candidate canonical
ZNF592	ZNF592_representative_N1	C2H2 ZF	ranking	candidate canonical
ZNF596	ZNF596_HEK293_ENCSR344SBD_merged_N1	C2H2 ZF	experiment	candidate canonical
ZNF600	ZNF600_HEK293_ENCSR017QBI_merged_N1	C2H2 ZF	experiment	candidate canonical
ZNF607	ZNF607_HepG2_ENCSR788SXN_merged_N2	C2H2 ZF	experiment	candidate canonical
ZNF610	ZNF610_HEK293_ENCSR691TXI_merged_N1	C2H2 ZF	experiment	candidate canonical
ZNF624	ZNF624_A549_ENCSR419VVI_merged_N1	C2H2 ZF	experiment	candidate canonical
ZNF629	ZNF629_representativeHSAv2_N1	C2H2 ZF	ranking	candidate canonical
ZNF639	ZNF639_representative_N1	C2H2 ZF	ranking	candidate canonical
ZNF644	ZNF644_representative_N1	C2H2 ZF	ranking	candidate canonical
ZNF646	ZNF646_HepG2_ENCSR488ZNK_merged_N1	C2H2 ZF	experiment	candidate canonical
ZNF654	ZNF654_HEK293_ENCSR504VDV_merged_N1	C2H2 ZF	experiment	candidate canonical
ZNF664	ZNF664_HEK293_ENCSR714LZQ_merged_N1	C2H2 ZF	experiment	candidate canonical
ZNF668	ZNF668_K562_ENCSR682AAS_merged_N1	C2H2 ZF	experiment	candidate canonical
ZNF671	ZNF671_WTC11_ENCSR260GQA_merged_N1	C2H2 ZF	experiment	candidate canonical
ZNF672	ZNF672_HepG2_ENCSR263YXJ_merged_N1	C2H2 ZF	experiment	candidate canonical
ZNF677	ZNF677_HEK293_ENCSR279KDC_merged_N1	C2H2 ZF	experiment	candidate canonical
ZNF691	ZNF691_HepG2_ENCSR130ZAR_merged_N1	C2H2 ZF	experiment	candidate canonical
ZNF697	ZNF697_representativeHSAv2_N1	C2H2 ZF	ranking	candidate canonical
ZNF7	ZNF7_HepG2_ENCSR553QMC_merged_N1	C2H2 ZF	experiment	candidate canonical
ZNF710	ZNF710_HepG2_ENCSR670UEX_merged_N1	C2H2 ZF	experiment	candidate canonical
ZNF746	ZNF746_HepG2_ENCSR591MYB_merged_N1	C2H2 ZF	experiment	candidate canonical
ZNF76	ZNF76_representativeHSAv2_N1	C2H2 ZF	ranking	candidate canonical
ZNF770	ZNF770_representativeHSAv2_N1	C2H2 ZF	ranking	candidate canonical
ZNF772	ZNF772_HepG2_ENCSR248WAU_merged_N1	C2H2 ZF	experiment	candidate canonical
ZNF775	ZNF775_HepG2_ENCSR856QJP_merged_N1	C2H2 ZF	experiment	candidate canonical
ZNF781	ZNF781_HEK293_ENCSR777YSB_merged_N1	C2H2 ZF	experiment	candidate canonical
ZNF784	ZNF784_HepG2_ENCSR082QGT_merged_N1	C2H2 ZF	experiment	candidate canonical
ZNF79	ZNF79_K562_ENCSR995FUM_merged_N1	C2H2 ZF	experiment	candidate canonical
ZNF816	ZNF816_HepG2_ENCSR905BNO_merged_N1	C2H2 ZF	experiment	candidate canonical

ZNF83	ZNF83_K562_ENCSR257XVY_merged_N1	C2H2 ZF	experiment	candidate canonical
ZNF850	ZNF850_HepG2_ENCSR756CJS_merged_N1	C2H2 ZF	experiment	candidate canonical
ZNF865	ZNF865_HepG2_ENCSR891KPP_merged_N1	C2H2 ZF	experiment	candidate canonical
ZSCAN12	ZSCAN12_HepG2_ENCSR574YRZ_merged_N1	C2H2 ZF	experiment	candidate canonical
ZSCAN16	ZSCAN16_HEK293_ENCSR864VJE_merged_N1	C2H2 ZF	experiment	candidate canonical
ZSCAN20	ZSCAN20_A549_ENCSR548FLW_merged_N1	C2H2 ZF	experiment	candidate canonical
ZSCAN21	ZSCAN21_representativeHSAv2_N1	C2H2 ZF	ranking	candidate canonical
ZSCAN25	ZSCAN25_HepG2_ENCSR037LQB_merged_N1	C2H2 ZF	experiment	candidate canonical
ZSCAN30	ZSCAN30_HEK293_ENCSR768VNZ_merged_N1	C2H2 ZF	experiment	candidate canonical
ZSCAN5C	ZSCAN5C_HEK293_ENCSR731AGO_merged_N1	C2H2 ZF	experiment	candidate canonical
ZSCAN9	ZSCAN9_HepG2_ENCSR712FAM_merged_N1	C2H2 ZF	experiment	candidate canonical
ZXDB	ZXDB_HEK293_ENCSR559IOZ_merged_N1	C2H2 ZF	experiment	candidate canonical
ZNF12	ZNF12_K562_ENCSR041YBR_merged_N1	C2H2 ZF	experiment	candidate canonical
ZNF2	ZNF2_HEK293_ENCSR011CKE_merged_N1	C2H2 ZF	experiment	candidate canonical
ZNF281	ZNF281_WTC11_ENCSR032DGZ_N1	C2H2 ZF	experiment	canonical
MYPOP	MYPOP_HepG2_ENCSR056SXR_N1	Myb/SANT	experiment	candidate canonical
ZKSCAN8	ZKSCAN8_WTC11_ENCSR115QIT_N1	C2H2 ZF	experiment	candidate canonical
E2F1	E2F1_K562_ENCSR153DWR_N1	E2F	experiment	canonical
ZBTB1	ZBTB1_K562_ENCSR166BAY_N2	C2H2 ZF	experiment	candidate canonical
ZBTB43	ZBTB43_WTC11_ENCSR199PXS_N2	C2H2 ZF	experiment	canonical
ZNF121	ZNF121_WTC11_ENCSR203PCF_N1	C2H2 ZF	experiment	candidate canonical
ZNF219	ZNF219_WTC11_ENCSR214RUE_N1	C2H2 ZF	experiment	canonical
USF2	USF2_WTC11_ENCSR229QAN_N1	bHLH	experiment	canonical
NFE2L1	NFE2L1_WTC11_ENCSR245QUM_N1	bZIP	experiment	canonical
ZNF564	ZNF564_HepG2_ENCSR397EOT_N1	C2H2 ZF	experiment	candidate canonical
ZFAT	ZFAT_HepG2_ENCSR409DYM_N1	C2H2 ZF	experiment	candidate canonical
ATF3	ATF3_K562_ENCSR568ZXG_N1	bZIP	experiment	canonical
ZNF184	ZNF184_WTC11_ENCSR640STN_N1	C2H2 ZF	experiment	candidate canonical
SP4	SP4_HepG2_ENCSR642PQK_N1	C2H2 ZF	experiment	canonical
NRF1	NRF1_HepG2_ENCSR689JMY_N1	Unknown	experiment	canonical
ZNF317	ZNF317_WTC11_ENCSR795KRU_N1	C2H2 ZF	experiment	candidate canonical
ZBTB43	ZBTB43_K562_ENCSR800KMQ_N1	C2H2 ZF	experiment	candidate canonical

ZNF841	ZNF841_HepG2_ENCSR808FZR_N1	C2H2 ZF	experiment	candidate canonical
MYBL2	MYBL2_WTC11_ENCSR859ZAC_N1	Myb/SANT	experiment	candidate canonical
POU2F1	POU2F1_HepG2_ENCSR871EQD_N1	Homeodomain; POU	experiment	canonical
ZBTB33	ZBTB33_K562_ENCSR955LXM_N1	C2H2 ZF	experiment	canonical
ZNF317	ZNF317_K562_ENCSR976MXN_N1	C2H2 ZF	experiment	candidate canonical

S2 Table. Frequencies of canonical binding, tethered binding and co-binding sites

TF (TF family)	Biosample	Canonical motif ID	Possible of co-binding TF (family)	X	Y	Z	Total no. of ChIP-seq peaks	Type of co-occurrence
ADNP (Homeodomain)	HepG2	ADNP_representativeHSAv2_N3	ZNF135 (C2H2 ZF)	14.60%	12.88%	72.52%	2740	Co-binding most frequent
AHDC1 (AT hook)	HepG2	AHDC1_HepG2_ENCSR168AUX_merged_N3	HNF4G (Nuclear recept	27.41%	55.09%	17.50%	3291	Tethered binding most frequent
AHDC1 (AT hook)	HepG2	AHDC1_HepG2_ENCSR168AUX_merged_N3	FOXC2 (Forkhead)	26.99%	59.17%	13.84%	3620	Tethered binding most frequent
AR (Nuclear receptor)	WTC11	AR_WTC11_ENCSR762LIP_merged_N3	ZNF460 (C2H2 ZF)	8.95%	31.99%	59.06%	447	Co-binding most frequent
ARID3A (ARID/BRIGHT)	HepG2	MA0151.1 from JASPAR	FOXC2 (Forkhead)	0.00%	100.00%	0.00%	8515	Tethered binding most frequent
ARID3A (ARID/BRIGHT)	K562	MA0151.1 from JASPAR	CTCF (C2H2 ZF)	0.00%	100.00%	0.00%	5508	Tethered binding most frequent
ARID3A (ARID/BRIGHT)	K562	MA0151.1 from JASPAR	GATA2 (GATA)	0.00%	100.00%	0.00%	3617	Tethered binding most frequent
ARID5B (ARID/BRIGHT)	HepG2	ARID5B_HepG2_ENCSR730DZO_merged_N2	RARG (Nuclear recepto	42.82%	41.76%	15.42%	26889	Tethered binding less frequent than canonical binding but more frequent than co-binding
ARID5B (ARID/BRIGHT)	HepG2	ARID5B_HepG2_ENCSR730DZO_merged_N2	FOXC2 (Forkhead)	41.43%	49.64%	8.93%	31098	Tethered binding most frequent
ARNT (bHLH)	K562	ARNT_HepG2_ENCSR029IBC_merged_N1	ZBTB26 (C2H2 ZF)	57.95%	39.03%	3.03%	5947	Tethered binding less frequent than canonical binding but more frequent than co-binding
ARNTL (bHLH)	HepG2	ARNTL_HepG2_ENCSR794LVK_merged_N1	PRDM9 (C2H2 ZF)	9.75%	74.62%	15.62%	10631	Tethered binding most frequent
ATF1 (bZIP)	K562	ATF1_representative_N1	SP2 (C2H2 ZF)	30.32%	27.76%	41.91%	742	Co-binding most frequent

ATF1 (bZIP)	HepG2	ATF1_representative_N1	ELK4 (Ets)	43.47%	37.98%	18.56%	1385	Tethered binding less frequent than canonical binding but more frequent than co-binding
ATF2 (bZIP)	GM12878	ATF2_representativeHSAv2_N1	RUNX1 (Runt)	33.59%	60.58%	5.83%	9375	Tethered binding most frequent
ATF3 (bZIP)	GM12878	ATF3_rv3	TFEC (bHLH)	8.79%	84.42%	6.79%	2105	Tethered binding most frequent
ATF3 (bZIP)	GM12878	ATF3_rv3	MITF (bHLH)	7.67%	84.57%	7.76%	2126	Tethered binding most frequent
ATF3 (bZIP)	H1	ATF3_rv3	USF2 (bHLH)	24.12%	71.30%	4.58%	2815	Tethered binding most frequent
ATF3 (bZIP)	H1	ATF3_rv3	TFEC (bHLH)	19.72%	74.01%	6.27%	3109	Tethered binding most frequent
ATF3 (bZIP)	HepG2	ATF3_rv3	USF2 (bHLH)	18.77%	74.38%	6.85%	2248	Tethered binding most frequent
ATF3 (bZIP)	HepG2	ATF3_rv3	ARNTL (bHLH)	15.84%	77.30%	6.86%	2538	Tethered binding most frequent
ATF3 (bZIP)	K562	ATF3_rv3	BHLHE41 (bHLH)	61.20%	29.88%	8.92%	9452	Tethered binding less frequent than canonical binding but more frequent than co-binding
ATF6 (bZIP)	HepG2	M04254_2.00 from Cis-BP	REST (C2H2 ZF)	19.75%	77.14%	3.11%	3407	Tethered binding most frequent
BACH1 (bZIP)	GM12878	BACH1_rv3	PATZ1 (C2H2 ZF; AT h	17.70%	78.31%	3.99%	21901	Tethered binding most frequent
BCL11A (C2H2 ZF)	GM12878	BCL11A_H1-hESC_ENCSR000BMJ_Rep1_N1	BATF3 (bZIP)	36.48%	57.97%	5.55%	13506	Tethered binding most frequent
BCL11A (C2H2 ZF)	GM12878	BCL11A_H1-hESC_ENCSR000BMJ_Rep1_N1	RUNX1 (Runt)	32.79%	59.89%	7.32%	14152	Tethered binding most frequent
BCL11A (C2H2 ZF)	GM12878	BCL11A_H1-hESC_ENCSR000BMJ_Rep1_N1	SPIB (Ets)	22.74%	69.57%	7.69%	18657	Tethered binding most frequent

BCL11A (C2H2 ZF)	HEK293	BCL11A_H1-hESC_ENCSR000BMJ_Rep1_N1	NR2F6 (Nuclear recepto	24.88%	64.24%	10.88%	9626	Tethered binding most frequent
BCL11B (C2H2 ZF)	HEK293	M04454_2.00 from Cis-BP	NR2C2 (Nuclear recepto	2.89%	95.92%	1.19%	6218	Tethered binding most frequent
BCL6 (C2H2 ZF)	HepG2	BCL6_rv3	FOXC2 (Forkhead)	41.53%	49.58%	8.89%	29077	Tethered binding most frequent
BCL6 (C2H2 ZF)	HepG2	BCL6_rv3	RARA (Nuclear recepto	48.02%	44.38%	7.61%	26359	Tethered binding less frequent than canonical binding but more frequent than co-binding
BHLHE40 (bHLH)	HepG2	BHLHE40_representative_N1	SP4 (C2H2 ZF)	28.39%	32.78%	38.83%	3054	Co-binding most frequent
BHLHE40 (bHLH)	HepG2	BHLHE40_representative_N1	FOXC2 (Forkhead)	70.51%	23.08%	6.41%	2669	Tethered binding less frequent than canonical binding but more frequent than co-binding
BHLHE40 (bHLH)	GM12878	BHLHE40_representative_N1	SP2 (C2H2 ZF)	18.26%	66.07%	15.67%	19609	Tethered binding most frequent
BHLHE40 (bHLH)	HepG2	BHLHE40_representative_N1	SP2 (C2H2 ZF)	22.03%	59.80%	18.17%	18550	Tethered binding most frequent
BRF2 (Unknown)	HepG2	BRF2_HepG2_ENCSR715EIP_merged_N1	ZNF93 (C2H2 ZF)	5.98%	93.38%	0.64%	786	Tethered binding most frequent
CDC5L (Myb/SANT)	K562	CDC5L_K562_ENCSR121PFY_merged_N3	FOXG1 (Forkhead)	5.39%	21.88%	72.72%	3857	Co-binding most frequent
CEBPA (bZIP)	HepG2	CEBPA_HepG2_ENCSR142IGM_merged_N1	FOXC2 (Forkhead)	60.37%	28.87%	10.76%	61677	Tethered binding less frequent than canonical binding but more frequent than co-binding

CEBPB (bZIP)	A549	CEBPB_representative_N1	FOXC2 (Forkhead)	70.22%	20.64%	9.14%	13766	Tethered binding less frequent than canonical binding but more frequent than co-binding
CEBPB (bZIP)	A549	CEBPB_representative_N1	FOXC2 (Forkhead)	77.55%	13.87%	8.58%	29167	Tethered binding less frequent than canonical binding but more frequent than co-binding
CEBPB (bZIP)	A549	CEBPB_representative_N1	FOXC2 (Forkhead)	69.41%	19.50%	11.09%	12769	Tethered binding less frequent than canonical binding but more frequent than co-binding
CREB1 (bZIP)	A549	CREB1_representative_N1	KLF15 (C2H2 ZF)	5.63%	82.36%	12.01%	8052	Tethered binding most frequent
CREB1 (bZIP)	A549	CREB1_representative_N1	PGR (Nuclear receptor)	50.63%	45.57%	3.79%	2609	Tethered binding less frequent than canonical binding but more frequent than co-binding
CREB1 (bZIP)	A549	CREB1_representative_N1	FOXC2 (Forkhead)	68.15%	26.69%	5.16%	697	Tethered binding less frequent than canonical binding but more frequent than co-binding
CREB1 (bZIP)	Ishikawa	CREB1_representative_N1	ELK4 (Ets)	43.11%	44.16%	12.72%	3136	Tethered binding most frequent
CREB1 (bZIP)	HepG2	CREB1_representative_N1	SP2 (C2H2 ZF)	57.88%	32.57%	9.55%	22309	Tethered binding less frequent than canonical binding but more frequent than co-binding
CREB1 (bZIP)	HepG2	CREB1_representative_N1	ELK4 (Ets)	40.74%	44.71%	14.55%	2364	Tethered binding most frequent
CREB1 (bZIP)	K562	CREB1_representative_N1	SP2 (C2H2 ZF)	18.86%	52.69%	28.46%	2551	Tethered binding most frequent

CREB1 (bZIP)	K562	CREB1_representative_N1	ELK4 (Ets)	40.47%	40.57%	18.96%	2031	Tethered binding most frequent
CREB1 (bZIP)	MCF-7	CREB1_representative_N1	SP2 (C2H2 ZF)	38.43%	51.89%	9.68%	20274	Tethered binding most frequent
CREB3 (bZIP)	HepG2	CREB3_representativeHSAv2_N1	REST (C2H2 ZF)	3.66%	95.11%	1.22%	573	Tethered binding most frequent
CREB3 (bZIP)	HepG2	CREB3_representativeHSAv2_N1	REST (C2H2 ZF)	9.73%	87.61%	2.65%	226	Tethered binding most frequent
CREB3 (bZIP)	HepG2	CREB3_representativeHSAv2_N1	USF1 (bHLH)	8.01%	91.03%	0.96%	312	Tethered binding most frequent
CREM (bZIP)	K562	CREM_rv3	ELK4 (Ets)	39.15%	54.14%	6.71%	16800	Tethered binding most frequent
CREM (bZIP)	WTC11	CREM_rv3	ELK4 (Ets)	28.19%	53.45%	18.36%	2898	Tethered binding most frequent
CREM (bZIP)	HepG2	CREM_rv3	ELK4 (Ets)	41.50%	28.67%	29.83%	1029	Co-binding less frequent than canonical binding but more frequent than tethered binding
CREM (bZIP)	HepG2	CREM_rv3	KLF12 (C2H2 ZF)	23.08%	38.63%	38.29%	1196	Tethered binding most frequent
CREM (bZIP)	GM12878	CREM_rv3	ELK4 (Ets)	30.53%	62.31%	7.16%	10797	Tethered binding most frequent
CREM (bZIP)	HepG2	CREM_rv3	THAP11 (THAP finger)	39.43%	55.42%	5.15%	5285	Tethered binding most frequent
CTCF (C2H2 ZF)	heart left ventric	CTCF_rv3	TLX3 (Homeodomain)	89.99%	0.58%	9.42%	10483	Co-binding less frequent than canonical binding but more frequent than tethered binding
DBP (bZIP)	HepG2	M04237_2.00 from Cis-BP	REST (C2H2 ZF)	6.12%	93.88%	0.00%	196	Tethered binding most frequent
DDIT3 (bZIP)	HepG2	DDIT3_rv3	ZNF610 (C2H2 ZF)	18.92%	76.54%	4.54%	1168	Tethered binding most frequent

DEAF1 (SAND)	K562	DEAF1_K562_ENCSR119FAD_merged_N2	CTCF (C2H2 ZF)	1.65%	90.10%	8.25%	4243	Tethered binding most frequent
DLX6 (Homeodomain)	HepG2	M04905_2.00 from Cis-BP	FOXC2 (Forkhead)	13.10%	82.33%	4.57%	2343	Tethered binding most frequent
DLX6 (Homeodomain)	HepG2	M04905_2.00 from Cis-BP	HNF4A (Nuclear recepto	11.90%	84.41%	3.69%	2656	Tethered binding most frequent
DMTF1 (Myb/SANT)	HepG2	DMTF1_representativeHSAv2_N1	PRDM9 (C2H2 ZF)	16.62%	47.68%	35.69%	367	Tethered binding most frequent
E2F1 (E2F)	HepG2	E2F1_representative_N1	NFYA (CBF/NF-Y)	65.56%	15.24%	19.21%	781	Co-binding less frequent than canonical binding but more frequent than tethered binding
E2F1 (E2F)	K562	E2F1_representative_N1	ATF6B (bZIP)	24.69%	69.71%	5.59%	8347	Tethered binding most frequent
E2F1 (E2F)	WTC11	E2F1_representative_N1	REST (C2H2 ZF)	34.39%	48.42%	17.19%	506	Tethered binding most frequent
E2F1 (E2F)	WTC11	E2F1_representative_N1	REST (C2H2 ZF)	48.85%	40.14%	11.01%	436	Tethered binding less frequent than canonical binding but more frequent than co-binding
E2F1 (E2F)	WTC11	E2F1_representative_N1	MLX (bHLH)	48.30%	31.85%	19.84%	383	Tethered binding less frequent than canonical binding but more frequent than co-binding
E2F4 (E2F)	K562	E2F4_representativeHSAv2_N4	SP2 (C2H2 ZF)	15.41%	57.72%	26.87%	18889	Tethered binding most frequent
E2F4 (E2F)	K562	E2F4_representativeHSAv2_N4	NFYA (CBF/NF-Y)	71.89%	19.48%	8.63%	9918	Tethered binding less frequent than canonical binding but more frequent than co-binding

E2F4 (E2F)	WTC11	E2F4_representativeHSAv2_N4	LIN54 (TCR/CxC)	83.42%	8.93%	7.65%	392	Tethered binding less frequent than canonical binding but more frequent than co-binding
E2F4 (E2F)	WTC11	E2F4_representativeHSAv2_N4	REST (C2H2 ZF)	41.21%	36.59%	22.20%	563	Tethered binding less frequent than canonical binding but more frequent than co-binding
E2F4 (E2F)	K562	E2F4_representativeHSAv2_N4	LIN54 (TCR/CxC)	60.29%	21.45%	18.26%	690	Tethered binding less frequent than canonical binding but more frequent than co-binding
E2F4 (E2F)	K562	E2F4_representativeHSAv2_N4	SP2 (C2H2 ZF)	23.00%	31.13%	45.87%	787	Co-binding most frequent
E2F4 (E2F)	K562	E2F4_representativeHSAv2_N4	NFYA (CBF/NF-Y)	62.33%	18.98%	18.68%	669	Tethered binding less frequent than canonical binding but more frequent than co-binding
E2F4 (E2F)	HepG2	E2F4_representativeHSAv2_N4	SP2 (C2H2 ZF)	12.18%	47.25%	40.57%	6413	Tethered binding most frequent
E2F4 (E2F)	HepG2	E2F4_representativeHSAv2_N4	NFYA (CBF/NF-Y)	56.55%	26.95%	16.50%	4631	Tethered binding less frequent than canonical binding but more frequent than co-binding
E2F5 (E2F)	WTC11	E2F5_rv3	KLF12 (C2H2 ZF)	13.38%	34.43%	52.20%	8380	Co-binding most frequent
E2F5 (E2F)	HepG2	E2F5_rv3	SP2 (C2H2 ZF)	22.88%	34.51%	42.61%	12115	Co-binding most frequent
E2F5 (E2F)	HepG2	E2F5_rv3	NFYA (CBF/NF-Y)	83.53%	8.26%	8.21%	8648	Tethered binding less frequent than canonical binding but more frequent than co-binding

E2F6 (E2F)	K562	E2F6_representative_N1	MYC (bHLH)	55.04%	36.77%	8.19%	32101	Tethered binding less frequent than canonical binding but more frequent than co-binding
E2F6 (E2F)	A549	E2F6_representative_N1	MAX (bHLH)	69.26%	23.36%	7.38%	9209	Tethered binding less frequent than canonical binding but more frequent than co-binding
E2F6 (E2F)	K562	E2F6_representative_N1	MYC (bHLH)	54.94%	35.89%	9.17%	17491	Tethered binding less frequent than canonical binding but more frequent than co-binding
E2F8 (E2F)	HepG2	E2F8_representativeHSAv2_N1	SP2 (C2H2 ZF)	14.19%	56.85%	28.96%	6496	Tethered binding most frequent
E2F8 (E2F)	GM12878	E2F8_representativeHSAv2_N1	ELK4 (Ets)	31.51%	47.89%	20.60%	4151	Tethered binding most frequent
E2F8 (E2F)	K562	E2F8_representativeHSAv2_N1	ZBTB7A (C2H2 ZF)	36.93%	52.07%	11.01%	7260	Tethered binding most frequent
EGR1 (C2H2 ZF)	GM12878	EGR1_representativeHSAv2_N2	GMEB1 (SAND)	58.32%	21.21%	20.47%	10314	Tethered binding less frequent than canonical binding but more frequent than co-binding
EGR1 (C2H2 ZF)	GM12878	EGR1_representativeHSAv2_N2	ELK4 (Ets)	67.36%	17.08%	15.56%	9800	Tethered binding less frequent than canonical binding but more frequent than co-binding
EGR1 (C2H2 ZF)	HCT116	EGR1_representativeHSAv2_N2	FOS (bZIP)	72.01%	18.03%	9.96%	8686	Tethered binding less frequent than canonical binding but more frequent than co-binding
ELF1 (Ets)	K562	ELF1_representativeHSAv2_N2	ZBTB7A (C2H2 ZF)	5.42%	50.67%	43.92%	17228	Tethered binding most frequent

ELF1 (Ets)	HepG2	ELF1_representativeHSAv2_N2	ZBTB7A (C2H2 ZF)	4.86%	46.93%	48.20%	11429	Co-binding most frequent
ELF1 (Ets)	HepG2	ELF1_representativeHSAv2_N2	KLF12 (C2H2 ZF)	23.27%	60.19%	16.54%	15952	Tethered binding most frequent
ELF1 (Ets)	HepG2	ELF1_representativeHSAv2_N2	THAP11 (THAP finger)	46.91%	43.30%	9.78%	11202	Tethered binding less frequent than canonical binding but more frequent than co-binding
ELF1 (Ets)	MCF-7	ELF1_representativeHSAv2_N2	ZBTB7A (C2H2 ZF)	4.68%	46.72%	48.60%	10203	Co-binding most frequent
ELF1 (Ets)	K562	ELF1_representativeHSAv2_N2	ZBTB7A (C2H2 ZF)	2.12%	19.40%	78.47%	1979	Co-binding most frequent
ELF1 (Ets)	K562	ELF1_representativeHSAv2_N2	KLF12 (C2H2 ZF)	26.63%	18.79%	54.58%	1964	Co-binding most frequent
ELF3 (Ets; AT hook)	HepG2	M04790_2.00 from Cis-BP	ELF1 (Ets)	11.07%	65.57%	23.37%	10618	Tethered binding most frequent
ELF3 (Ets; AT hook)	HepG2	M04790_2.00 from Cis-BP	FOXC2 (Forkhead)	33.05%	58.02%	8.93%	8709	Tethered binding most frequent
ELF4 (Ets)	WTC11	ELF4_representativeHSAv2_N1	REST (C2H2 ZF)	14.48%	75.78%	9.74%	801	Tethered binding most frequent
ELF4 (Ets)	K562	ELF4_representativeHSAv2_N1	TFEC (bHLH)	53.06%	35.99%	10.95%	2448	Tethered binding less frequent than canonical binding but more frequent than co-binding
ELF4 (Ets)	K562	ELF4_representativeHSAv2_N1	THAP11 (THAP finger)	72.79%	17.56%	9.64%	8296	Tethered binding less frequent than canonical binding but more frequent than co-binding
ELF4 (Ets)	HepG2	ELF4_representativeHSAv2_N1	SP2 (C2H2 ZF)	34.17%	17.37%	48.46%	518	Co-binding most frequent
ELK1 (Ets)	IMR-90	ELK1_representativeHSAv2_N1	SRF (MADS box)	77.46%	14.92%	7.63%	590	Tethered binding less frequent than canonical binding but more frequent than co-binding

EMX1 (Homeodomain)	WTC11	M03152_2.00 from Cis-BP	ZNF460 (C2H2 ZF)	2.67%	96.74%	0.59%	337	Tethered binding most frequent
ERF (Ets)	HepG2	M02968_2.00 from Cis-BP	REST (C2H2 ZF)	41.35%	54.23%	4.42%	6223	Tethered binding most frequent
ESRRA (Nuclear receptor)	WTC11	ESRRA_rv3	REST (C2H2 ZF)	25.50%	63.77%	10.73%	1463	Tethered binding most frequent
ESRRA (Nuclear receptor)	WTC11	ESRRA_rv3	REST (C2H2 ZF)	30.85%	58.82%	10.33%	1287	Tethered binding most frequent
ETS1 (Ets)	GM12878	ETS1_representative_N1	THAP11 (THAP finger)	63.92%	22.92%	13.16%	6920	Tethered binding less frequent than canonical binding but more frequent than co-binding
ETS1 (Ets)	GM12878	ETS1_representative_N1	THAP11 (THAP finger)	61.90%	23.65%	14.46%	6986	Tethered binding less frequent than canonical binding but more frequent than co-binding
ETS1 (Ets)	K562	ETS1_representative_N1	THAP11 (THAP finger)	42.63%	42.36%	15.01%	5984	Tethered binding less frequent than canonical binding but more frequent than co-binding
ETS1 (Ets)	A549	ETS1_representative_N1	THAP11 (THAP finger)	53.54%	33.76%	12.70%	3984	Tethered binding less frequent than canonical binding but more frequent than co-binding
ETS1 (Ets)	GM23338	ETS1_representative_N1	THAP11 (THAP finger)	48.40%	33.57%	18.03%	6047	Tethered binding less frequent than canonical binding but more frequent than co-binding
ETS1 (Ets)	GM23338	ETS1_representative_N1	THAP11 (THAP finger)	61.09%	26.83%	12.08%	5490	Tethered binding less frequent than canonical binding but more frequent than co-binding

ETS1 (Ets)	HepG2	ETS1_representative_N1	HNF4A (Nuclear recepto	25.29%	68.76%	5.95%	3697	Tethered binding most frequent
ETS1 (Ets)	HepG2	ETS1_representative_N1	TFAP4 (bHLH)	38.05%	55.47%	6.48%	2594	Tethered binding most frequent
ETV1 (Ets)	K562	ETV1_K562_ENCSR277DMR_merged_N1	GATA2 (GATA)	76.09%	17.95%	5.96%	9595	Tethered binding less frequent than canonical binding but more frequent than co-binding
ETV4 (Ets)	HepG2	ETV4_representativeHSAv2_N1	FOXC2 (Forkhead)	79.68%	12.28%	8.04%	4129	Tethered binding less frequent than canonical binding but more frequent than co-binding
ETV4 (Ets)	HepG2	ETV4_representativeHSAv2_N1	PATZ1 (C2H2 ZF; AT h	39.05%	27.72%	33.23%	5011	Co-binding less frequent than canonical binding but more frequent than tethered binding
ETV4 (Ets)	HepG2	ETV4_representativeHSAv2_N1	FOXC2 (Forkhead)	64.76%	27.24%	8.00%	9251	Tethered binding less frequent than canonical binding but more frequent than co-binding
ETV5 (Ets)	HepG2	ETV5_representativeHSAv2_N1	FOXC2 (Forkhead)	64.68%	29.36%	5.97%	12485	Tethered binding less frequent than canonical binding but more frequent than co-binding
ETV5 (Ets)	K562	ETV5_representativeHSAv2_N1	GATA5 (GATA)	80.82%	14.18%	5.00%	4880	Tethered binding less frequent than canonical binding but more frequent than co-binding

ETV6 (Ets)	GM12878	ETV6_representativeHSAv2_N2	IRF1 (IRF)	54.72%	31.86%	13.42%	9640	Tethered binding less frequent than canonical binding but more frequent than co-binding
ETV6 (Ets)	HepG2	ETV6_representativeHSAv2_N2	REST (C2H2 ZF)	44.98%	46.49%	8.53%	9706	Tethered binding most frequent
FOS (bZIP)	GM12878	FOS_representative_N1	NFYA (CBF/NF-Y)	1.35%	96.45%	2.20%	1409	Tethered binding most frequent
FOS (bZIP)	GM12878	FOS_representative_N1	NFYA (CBF/NF-Y)	1.16%	96.77%	2.07%	1546	Tethered binding most frequent
FOS (bZIP)	GM12878	FOS_representative_N1	PATZ1 (C2H2 ZF; AT h	2.65%	95.98%	1.37%	1243	Tethered binding most frequent
FOS (bZIP)	K562	FOS_representative_N1	NFYA (CBF/NF-Y)	58.99%	37.25%	3.75%	13298	Tethered binding less frequent than canonical binding but more frequent than co-binding
FOS (bZIP)	K562	FOS_representative_N1	NFYA (CBF/NF-Y)	62.13%	34.10%	3.77%	12662	Tethered binding less frequent than canonical binding but more frequent than co-binding
FOS (bZIP)	K562	FOS_representative_N1	SP2 (C2H2 ZF)	72.44%	22.95%	4.61%	10829	Tethered binding less frequent than canonical binding but more frequent than co-binding
FOSL1 (bZIP)	HepG2	FOSL1_representative_N1	FOXD2 (Forkhead)	82.11%	6.12%	11.77%	2795	Co-binding less frequent than canonical binding but more frequent than tethered binding
FOXA1 (Forkhead)	A549	FOXA1_representativeHSAv2_N3	PGR (Nuclear receptor)	35.29%	40.31%	24.40%	5016	Tethered binding most frequent

FOXA1 (Forkhead)	A549	FOXA1_representativeHSAv2_N3	ATF3 (bZIP)	64.51%	23.75%	11.74%	24278	Tethered binding less frequent than canonical binding but more frequent than co-binding
FOXC1 (Forkhead)	HepG2	M03013_2.00 from Cis-BP	BHLHE41 (bHLH)	22.43%	76.38%	1.18%	1266	Tethered binding most frequent
FOXF2 (Forkhead)	A549	FOXF2_A549_ENCSR445FHB_merged_N1	FOSL2 (bZIP)	58.82%	17.17%	24.01%	1258	Co-binding less frequent than canonical binding but more frequent than tethered binding
FOXJ3 (Forkhead)	K562	FOXJ3_representativeHSAv2_N1	RFX3 (RFX)	10.27%	78.08%	11.64%	146	Tethered binding most frequent
FOXJ3 (Forkhead)	HepG2	FOXJ3_representativeHSAv2_N1	NR2F6 (Nuclear recepto	33.01%	48.49%	18.50%	827	Tethered binding most frequent
FOXJ3 (Forkhead)	SK-N-SH	FOXJ3_representativeHSAv2_N1	HAND2 (bHLH)	20.04%	74.85%	5.11%	998	Tethered binding most frequent
FOXJ3 (Forkhead)	SK-N-SH	FOXJ3_representativeHSAv2_N1	TFAP2B (AP-2)	22.25%	71.80%	5.96%	890	Tethered binding most frequent
FOXK1 (Forkhead)	WTC11	FOXK1_rv3	ELK4 (Ets)	28.65%	61.20%	10.16%	2894	Tethered binding most frequent
FOXK1 (Forkhead)	WTC11	FOXK1_rv3	ZNF93 (C2H2 ZF)	13.25%	77.73%	9.02%	5043	Tethered binding most frequent
FOXK1 (Forkhead)	HepG2	FOXK1_rv3	ELK4 (Ets)	44.34%	49.91%	5.76%	12764	Tethered binding most frequent
FOXM1 (Forkhead)	GM12878	FOXM1_representative_N1	JUN (bZIP)	28.25%	63.30%	8.45%	6946	Tethered binding most frequent
FOXM1 (Forkhead)	GM12878	FOXM1_representative_N1	IRF1 (IRF)	18.71%	72.19%	9.10%	9166	Tethered binding most frequent
FOXM1 (Forkhead)	GM12878	FOXM1_representative_N1	RUNX1 (Runt)	30.60%	62.43%	6.97%	6785	Tethered binding most frequent
FOXM1 (Forkhead)	SK-N-SH	FOXM1_representative_N1	JUND (bZIP)	14.89%	75.78%	9.33%	4096	Tethered binding most frequent
FOXM1 (Forkhead)	MCF-7	FOXM1_representative_N1	ESR2 (Nuclear receptor	25.55%	70.24%	4.21%	3061	Tethered binding most frequent

FOXM1 (Forkhead)	Ishikawa	FOXM1_representative_N1	TEAD3 (TEA)	31.98%	62.30%	5.72%	3199	Tethered binding most frequent
FOXM1 (Forkhead)	K562	FOXM1_representative_N1	LIN54 (TCR/CxC)	34.78%	47.67%	17.55%	4502	Tethered binding most frequent
FOXM1 (Forkhead)	K562	FOXM1_representative_N1	GATA2 (GATA)	31.88%	62.93%	5.19%	6355	Tethered binding most frequent
FOXO1 (Forkhead)	HepG2	FOXO1_HepG2_ENCSR321OAA_merged_N1	HNF4G (Nuclear recept	27.88%	64.12%	8.00%	15134	Tethered binding most frequent
FOXP1 (Forkhead)	K562	FOXP1_representative_N1	RFX3 (RFX)	27.29%	61.45%	11.26%	1048	Tethered binding most frequent
FOXP2 (Forkhead)	PFSK-1	FOXP2_PFSK-1_ENCSR000BGA_merged_N1	PATZ1 (C2H2 ZF; AT h	9.20%	86.02%	4.78%	17495	Tethered binding most frequent
FOXP4 (Forkhead)	WTC11	FOXP4_representativeHSAv2_N1	REST (C2H2 ZF)	21.92%	76.01%	2.07%	3039	Tethered binding most frequent
GATA1 (GATA)	erythroblast	GATA1_representative_N2	NEUROD2 (bHLH)	61.41%	29.92%	8.67%	10643	Tethered binding less frequent than canonical binding but more frequent than co-binding
GATA2 (GATA)	K562	GATA2_representativeHSAv2_N1	MYOG (bHLH)	0.00%	100.00%	0.00%	2536	Tethered binding most frequent
GATA2 (GATA)	endothelial-cell-	GATA2_representativeHSAv2_N1	ERG (Ets)	0.00%	100.00%	0.00%	5786	Tethered binding most frequent
GATA2 (GATA)	HepG2	GATA2_representativeHSAv2_N1	FOXC2 (Forkhead)	0.00%	100.00%	0.00%	2083	Tethered binding most frequent
GATA3 (GATA)	T47D	GATA3_representative_N1	FOXC2 (Forkhead)	0.00%	100.00%	0.00%	9571	Tethered binding most frequent
GATA3 (GATA)	SK-N-SH	GATA3_representative_N1	FOS (bZIP)	0.00%	100.00%	0.00%	20924	Tethered binding most frequent
GATA3 (GATA)	A549	GATA3_representative_N1	BATF3 (bZIP)	0.00%	100.00%	0.00%	6791	Tethered binding most frequent
GATA3 (GATA)	A549	GATA3_representative_N1	FOXC2 (Forkhead)	0.00%	100.00%	0.00%	5077	Tethered binding most frequent
GATA3 (GATA)	MCF-7	GATA3_representative_N1	FOXC2 (Forkhead)	0.00%	100.00%	0.00%	2225	Tethered binding most frequent

GATA3 (GATA)	MCF-7	GATA3_representative_N1	FOXD2 (Forkhead)	0.00%	100.00%	0.00%	2252	Tethered binding most frequent
GATA3 (GATA)	SH-SY5Y	GATA3_representative_N1	HAND2 (bHLH)	0.00%	100.00%	0.00%	2819	Tethered binding most frequent
GATA3 (GATA)	MCF-7	GATA3_representative_N1	FOXC2 (Forkhead)	0.00%	100.00%	0.00%	10515	Tethered binding most frequent
GATA4 (GATA)	HepG2	GATA4_HepG2_ENCSR590CNM_merged_N1	FOXC2 (Forkhead)	25.15%	64.87%	9.99%	3094	Tethered binding most frequent
GATA4 (GATA)	HepG2	GATA4_HepG2_ENCSR590CNM_merged_N1	TCF7L2 (HMG/Sox)	33.84%	57.82%	8.34%	2577	Tethered binding most frequent
GATAD2A (GATA)	HepG2	GATAD2A_K562_ENCSR160QYK_Rep2_N1	CEBPD (bZIP)	68.65%	25.15%	6.20%	18236	Tethered binding less frequent than canonical binding but more frequent than co-binding
GATAD2A (GATA)	HepG2	GATAD2A_K562_ENCSR160QYK_Rep2_N1	FOXC2 (Forkhead)	53.48%	38.27%	8.25%	22113	Tethered binding less frequent than canonical binding but more frequent than co-binding
GFI1 (C2H2 ZF)	HepG2	GFI1_HepG2_ENCSR849FVL_merged_N1	FOXC2 (Forkhead)	41.36%	45.05%	13.59%	3798	Tethered binding most frequent
GFI1 (C2H2 ZF)	HepG2	GFI1_HepG2_ENCSR849FVL_merged_N1	THRB (Nuclear receptor	42.48%	42.32%	15.20%	3618	Tethered binding less frequent than canonical binding but more frequent than co-binding
GMEB1 (SAND)	K562	GMEB1_representativeHSAv2_N1	SP2 (C2H2 ZF)	12.38%	50.47%	37.15%	2972	Tethered binding most frequent
GMEB1 (SAND)	HepG2	GMEB1_representativeHSAv2_N1	THAP11 (THAP finger)	42.09%	47.32%	10.59%	4514	Tethered binding most frequent
GMEB1 (SAND)	HepG2	GMEB1_representativeHSAv2_N1	ELF4 (Ets)	37.32%	52.06%	10.63%	4960	Tethered binding most frequent

GMEB1 (SAND)	K562	GMEB1_representativeHSAv2_N1	ATF1 (bZIP)	41.60%	20.87%	37.52%	3502	Co-binding less frequent than canonical binding but more frequent than tethered binding
GTF2I (GTF2I-like)	WTC11	GTF2I_representativeHSAv2_N1	ZNF787 (C2H2 ZF)	13.84%	40.03%	46.13%	672	Co-binding most frequent
HES1 (bHLH)	K562	M04090_2.00 from Cis-BP	GATA3 (GATA)	33.00%	64.40%	2.61%	3452	Tethered binding most frequent
HES1 (bHLH)	K562	M04090_2.00 from Cis-BP	RUNX1 (Runt)	38.38%	57.81%	3.81%	2913	Tethered binding most frequent
HES2 (bHLH)	A549	HES2_A549_ENCSR686BIE_merged_N4	CEBPA (bZIP)	14.76%	73.56%	11.67%	3915	Tethered binding most frequent
HES2 (bHLH)	A549	HES2_A549_ENCSR686BIE_merged_N4	BACH1 (bZIP)	31.50%	61.05%	7.45%	2657	Tethered binding most frequent
HES2 (bHLH)	A549	HES2_A549_ENCSR686BIE_merged_N4	CEBPD (bZIP)	11.22%	75.67%	13.11%	1159	Tethered binding most frequent
HES2 (bHLH)	A549	HES2_A549_ENCSR686BIE_merged_N4	CEBPE (bZIP)	17.26%	68.81%	13.94%	904	Tethered binding most frequent
HES2 (bHLH)	A549	HES2_A549_ENCSR686BIE_merged_N4	FOSL1 (bZIP)	26.76%	63.19%	10.05%	766	Tethered binding most frequent
HES2 (bHLH)	A549	HES2_A549_ENCSR686BIE_merged_N4	CEBPA (bZIP)	15.89%	72.15%	11.96%	2474	Tethered binding most frequent
HES2 (bHLH)	A549	HES2_A549_ENCSR686BIE_merged_N4	FOXC2 (Forkhead)	15.75%	51.78%	32.47%	1429	Tethered binding most frequent
HES2 (bHLH)	A549	HES2_A549_ENCSR686BIE_merged_N4	CEBPD (bZIP)	15.76%	74.39%	9.85%	5747	Tethered binding most frequent

HES2 (bHLH)	A549	HES2_A549_ENCSR686BIE_merged_N4	CEBPE (bZIP)	15.79%	74.52%	9.69%	5777	Tethered binding most frequent
HES2 (bHLH)	A549	HES2_A549_ENCSR686BIE_merged_N4	BNC2 (C2H2 ZF)	24.97%	67.25%	7.79%	4494	Tethered binding most frequent
HES2 (bHLH)	A549	HES2_A549_ENCSR686BIE_merged_N4	CEBPE (bZIP)	18.11%	73.08%	8.82%	6340	Tethered binding most frequent
HES2 (bHLH)	A549	HES2_A549_ENCSR686BIE_merged_N4	CEBPA (bZIP)	15.79%	74.92%	9.29%	6806	Tethered binding most frequent
HES2 (bHLH)	A549	HES2_A549_ENCSR686BIE_merged_N4	FOSL2 (bZIP)	23.04%	69.23%	7.73%	5547	Tethered binding most frequent
HES2 (bHLH)	A549	HES2_A549_ENCSR686BIE_merged_N4	FOXC2 (Forkhead)	14.13%	48.00%	37.86%	3283	Tethered binding most frequent
HES2 (bHLH)	A549	HES2_A549_ENCSR686BIE_merged_N4	CEBPE (bZIP)	19.04%	71.54%	9.42%	5084	Tethered binding most frequent
HES2 (bHLH)	A549	HES2_A549_ENCSR686BIE_merged_N4	CEBPA (bZIP)	15.78%	74.60%	9.62%	5697	Tethered binding most frequent
HES2 (bHLH)	A549	HES2_A549_ENCSR686BIE_merged_N4	JUND (bZIP)	25.07%	67.43%	7.49%	4443	Tethered binding most frequent
HES2 (bHLH)	A549	HES2_A549_ENCSR686BIE_merged_N4	CEBPA (bZIP)	14.89%	73.53%	11.58%	3136	Tethered binding most frequent
HES2 (bHLH)	A549	HES2_A549_ENCSR686BIE_merged_N4	CEBPE (bZIP)	17.02%	72.18%	10.79%	2984	Tethered binding most frequent
HES2 (bHLH)	A549	HES2_A549_ENCSR686BIE_merged_N4	FOXC2 (Forkhead)	15.76%	49.48%	34.75%	1643	Tethered binding most frequent

HES2 (bHLH)	A549	HES2_A549_ENCSR686BIE_merged_N4	CEBPA (bZIP)	16.39%	71.64%	11.96%	2257	Tethered binding most frequent
HES2 (bHLH)	A549	HES2_A549_ENCSR686BIE_merged_N4	CEBPE (bZIP)	20.69%	68.63%	10.69%	2040	Tethered binding most frequent
HES2 (bHLH)	A549	HES2_A549_ENCSR686BIE_merged_N4	FOXC2 (Forkhead)	14.87%	51.18%	33.94%	1311	Tethered binding most frequent
HES2 (bHLH)	A549	HES2_A549_ENCSR686BIE_merged_N4	CEBPE (bZIP)	15.58%	73.97%	10.45%	3350	Tethered binding most frequent
HES2 (bHLH)	A549	HES2_A549_ENCSR686BIE_merged_N4	CEBPA (bZIP)	12.94%	76.00%	11.07%	3633	Tethered binding most frequent
HES2 (bHLH)	A549	HES2_A549_ENCSR686BIE_merged_N4	CEBPA (bZIP)	13.86%	75.36%	10.78%	3933	Tethered binding most frequent
HES2 (bHLH)	A549	HES2_A549_ENCSR686BIE_merged_N4	FOSL2 (bZIP)	28.20%	64.92%	6.88%	2762	Tethered binding most frequent
HES2 (bHLH)	A549	HES2_A549_ENCSR686BIE_merged_N4	CEBPD (bZIP)	15.96%	74.04%	10.01%	4926	Tethered binding most frequent
HES2 (bHLH)	A549	HES2_A549_ENCSR686BIE_merged_N4	CEBPG (bZIP)	20.65%	70.26%	9.09%	4300	Tethered binding most frequent
HES2 (bHLH)	A549	HES2_A549_ENCSR686BIE_merged_N4	ATF3 (bZIP)	28.20%	64.04%	7.76%	3557	Tethered binding most frequent
HES2 (bHLH)	A549	HES2_A549_ENCSR686BIE_merged_N4	CEBPA (bZIP)	12.91%	75.62%	11.46%	2207	Tethered binding most frequent
HES2 (bHLH)	A549	HES2_A549_ENCSR686BIE_merged_N4	CEBPE (bZIP)	15.31%	73.17%	11.52%	2005	Tethered binding most frequent

HES2 (bHLH)	A549	HES2_A549_ENCSR686BIE_merged_N4	NFE2L1 (bZIP)	28.81%	63.87%	7.32%	1489	Tethered binding most frequent
HES2 (bHLH)	A549	HES2_A549_ENCSR686BIE_merged_N4	FOXC2 (Forkhead)	16.48%	52.85%	30.67%	1141	Tethered binding most frequent
HIC2 (C2H2 ZF)	HepG2	HIC2_HepG2_ENCSR015LYB_merged_N4	HNF4G (Nuclear recept	41.02%	42.43%	16.55%	9408	Tethered binding most frequent
HIC2 (C2H2 ZF)	HepG2	HIC2_HepG2_ENCSR015LYB_merged_N4	FOXC2 (Forkhead)	70.87%	23.04%	6.10%	7037	Tethered binding less frequent than canonical binding but more frequent than co-binding
HIVEP1 (C2H2 ZF)	HepG2	HIVEP1_representativeHSAv2_N2	NFKB2 (Rel)	74.49%	7.71%	17.79%	13133	Co-binding less frequent than canonical binding but more frequent than tethered binding
HIVEP1 (C2H2 ZF)	HepG2	HIVEP1_representativeHSAv2_N2	FOXC2 (Forkhead)	94.69%	2.38%	2.93%	12415	Co-binding less frequent than canonical binding but more frequent than tethered binding
HMBOX1 (Homeodomain)	K562	HMBOX1_K562_ENCSR757IIU_merged_N1	GATA2 (GATA)	100.00%	0.00%	0.00%	3431	not co-binding
HMG20A (HMG/Sox)	HepG2	HMG20A_HepG2_ENCSR072GJV_merged_N3	FOXC2 (Forkhead)	20.85%	71.33%	7.82%	8082	Tethered binding most frequent
HMG20A (HMG/Sox)	K562	HMG20A_HepG2_ENCSR072GJV_merged_N3	GATA2 (GATA)	7.50%	87.38%	5.13%	507	Tethered binding most frequent
HNF1A (Homeodomain)	HepG2	HNF1A_rv3	WT1 (C2H2 ZF)	23.01%	70.21%	6.78%	10933	Tethered binding most frequent

HNF1A (Homeodomain)	HepG2	HNF1A_rv3	FOXC2 (Forkhead)	60.04%	26.16%	13.80%	6502	Tethered binding less frequent than canonical binding but more frequent than co-binding
HNF1B (Homeodomain)	HepG2	HNF1B_HepG2_ENCSR127XTZ_merged_N1	FOXC2 (Forkhead)	49.57%	41.00%	9.42%	2695	Tethered binding less frequent than canonical binding but more frequent than co-binding
HNF4A (Nuclear receptor)	HepG2	HNF4A_representativeHSAv2_N1	FOXC2 (Forkhead)	71.38%	16.38%	12.24%	15993	Tethered binding less frequent than canonical binding but more frequent than co-binding
HNF4G (Nuclear receptor)	HepG2	HNF4G_representativeHSAv2_N1	CEBPA (bZIP)	84.33%	8.51%	7.16%	14799	Tethered binding less frequent than canonical binding but more frequent than co-binding
HNF4G (Nuclear receptor)	HepG2	HNF4G_representativeHSAv2_N1	FOXJ3 (Forkhead)	75.76%	12.03%	12.21%	7673	Co-binding less frequent than canonical binding but more frequent than tethered binding
HOMEZ (Homeodomain)	HepG2	HOMEZ_HepG2_ENCSR117CHD_merged_N1	FOXC2 (Forkhead)	48.37%	40.72%	10.90%	8943	Tethered binding less frequent than canonical binding but more frequent than co-binding
HOXA3 (Homeodomain)	HepG2	M10651_2.00	ELK4 (Ets)	4.83%	94.36%	0.80%	7469	Tethered binding most frequent
HOXA3 (Homeodomain)	HepG2	M10651_2.00	CREB5 (bZIP)	7.70%	91.44%	0.85%	4921	Tethered binding most frequent
HOXA3 (Homeodomain)	HepG2	M10651_2.00	YY1 (C2H2 ZF)	7.28%	92.04%	0.68%	5286	Tethered binding most frequent

HOXA5 (Homeodomain)	HepG2	HOXA5_HepG2_ENCSR869JZW_merged_N2	REST (C2H2 ZF)	15.64%	82.81%	1.54%	2985	Tethered binding most frequent
HOXB13 (Homeodomain)	A549	M05203_2.00 from Cis-BP	ZNF597 (C2H2 ZF)	4.33%	94.22%	1.44%	277	Tethered binding most frequent
HOXD1 (Homeodomain)	HepG2	HOXD1_HepG2_ENCSR359TWG_merged_N1	FOXC2 (Forkhead)	26.28%	65.06%	8.66%	1016	Tethered binding most frequent
IKZF1 (C2H2 ZF)	GM12878	IKZF1_K562_ENCSR948VFL_merged_N1	IRF1 (IRF)	0.00%	100.00%	0.00%	10327	Tethered binding most frequent
IKZF1 (C2H2 ZF)	GM12878	IKZF1_K562_ENCSR948VFL_merged_N1	RUNX1 (Runt)	0.00%	100.00%	0.00%	4631	Tethered binding most frequent
IKZF1 (C2H2 ZF)	K562	IKZF1_K562_ENCSR948VFL_merged_N1	STAT4 (STAT)	0.00%	100.00%	0.00%	31861	Tethered binding most frequent
IKZF1 (C2H2 ZF)	K562	IKZF1_K562_ENCSR948VFL_merged_N1	GATA2 (GATA)	0.00%	100.00%	0.00%	12068	Tethered binding most frequent
IKZF1 (C2H2 ZF)	GM12878	IKZF1_K562_ENCSR948VFL_merged_N1	SPIB (Ets)	0.00%	100.00%	0.00%	22952	Tethered binding most frequent
IKZF1 (C2H2 ZF)	GM12878	IKZF1_K562_ENCSR948VFL_merged_N1	THAP11 (THAP finger)	0.00%	100.00%	0.00%	11140	Tethered binding most frequent
IKZF1 (C2H2 ZF)	GM12878	IKZF1_K562_ENCSR948VFL_merged_N1	NRF1 (Unknown)	0.00%	100.00%	0.00%	6429	Tethered binding most frequent
IKZF1 (C2H2 ZF)	GM12878	IKZF1_K562_ENCSR948VFL_merged_N1	SPIB (Ets)	0.00%	100.00%	0.00%	34917	Tethered binding most frequent
IRF1 (IRF)	K562	IRF1_representative_N1	KLF12 (C2H2 ZF)	0.99%	94.80%	4.21%	5176	Tethered binding most frequent
IRF1 (IRF)	K562	IRF1_representative_N1	SP1 (C2H2 ZF)	1.46%	94.55%	3.99%	4936	Tethered binding most frequent
IRF1 (IRF)	K562	IRF1_representative_N1	NFYA (CBF/NF-Y)	8.82%	87.04%	4.14%	2076	Tethered binding most frequent

IRF2 (IRF)	HepG2	IRF2_representative_N1	ELF5 (Ets)	44.46%	49.49%	6.05%	18933	Tethered binding most frequent
IRF3 (IRF)	SK-N-SH	M05532_2.00 from Cis-BP	NFYA (CBF/NF-Y)	2.83%	94.68%	2.49%	2088	Tethered binding most frequent
IRF3 (IRF)	SK-N-SH	M05532_2.00 from Cis-BP	NFYB (Unknown)	16.39%	81.44%	2.17%	598	Tethered binding most frequent
IRF3 (IRF)	SK-N-SH	M05532_2.00 from Cis-BP	KLF15 (C2H2 ZF)	2.95%	94.71%	2.33%	2100	Tethered binding most frequent
IRF4 (IRF)	GM12878	M03334_2.00 from Cis-BP	BATF (bZIP)	31.62%	62.06%	6.32%	14657	Tethered binding most frequent
IRF4 (IRF)	GM12878	M03334_2.00 from Cis-BP	SPIB (Ets)	13.19%	69.73%	17.08%	18370	Tethered binding most frequent
IRF9 (IRF)	K562	IRF9_K562_ENCSR926KTP_merged_N2	CEBPG (bZIP)	30.34%	63.28%	6.38%	3919	Tethered binding most frequent
ISL2 (Homeodomain)	HepG2	M05204_2.00 from Cis-BP	TCF7L2 (HMG/Sox)	16.19%	81.58%	2.23%	3279	Tethered binding most frequent
ISL2 (Homeodomain)	HepG2	M05204_2.00 from Cis-BP	FOXC1 (Forkhead)	7.59%	89.32%	3.10%	5653	Tethered binding most frequent
JUN (bZIP)	endothelial-cell-	JUN_representative HSAv2_N1	ERG (Ets)	66.79%	17.49%	15.71%	30712	Tethered binding less frequent than canonical binding but more frequent than co-binding
JUN (bZIP)	K562	JUN_representative HSAv2_N1	GATA5 (GATA)	81.21%	8.94%	9.85%	4800	Co-binding less frequent than canonical binding but more frequent than tethered binding
JUN (bZIP)	K562	JUN_representative HSAv2_N1	GATA2 (GATA)	81.61%	7.82%	10.57%	3632	Co-binding less frequent than canonical binding but more frequent than tethered binding
JUN (bZIP)	A549	JUN_representative HSAv2_N1	CTCF (C2H2 ZF)	25.44%	69.83%	4.73%	14406	Tethered binding most frequent

JUN (bZIP)	A549	JUN_representative HSAv2_N1	PGR (Nuclear receptor)	56.21%	35.22%	8.57%	7751	Tethered binding less frequent than canonical binding but more frequent than co-binding
JUN (bZIP)	A549	JUN_representative HSAv2_N1	PGR (Nuclear receptor)	58.51%	32.64%	8.85%	6927	Tethered binding less frequent than canonical binding but more frequent than co-binding
JUN (bZIP)	A549	JUN_representative HSAv2_N1	PRDM9 (C2H2 ZF)	20.11%	77.48%	2.41%	28588	Tethered binding most frequent
JUN (bZIP)	A549	JUN_representative HSAv2_N1	PGR (Nuclear receptor)	62.66%	30.20%	7.14%	10695	Tethered binding less frequent than canonical binding but more frequent than co-binding
JUN (bZIP)	HepG2	JUN_representative HSAv2_N1	FOXC2 (Forkhead)	58.86%	29.12%	12.02%	2871	Tethered binding less frequent than canonical binding but more frequent than co-binding
JUN (bZIP)	A549	JUN_representative HSAv2_N1	IRX1 (Homeodomain)	58.97%	31.03%	10.00%	10707	Tethered binding less frequent than canonical binding but more frequent than co-binding
JUN (bZIP)	A549	JUN_representative HSAv2_N1	PGR (Nuclear receptor)	47.04%	43.57%	9.39%	6679	Tethered binding less frequent than canonical binding but more frequent than co-binding
JUNB (bZIP)	A549	JUNB_representative_N1	FOXC2 (Forkhead)	70.82%	15.22%	13.96%	13423	Tethered binding less frequent than canonical binding but more frequent than co-binding

JUNB (bZIP)	A549	JUNB_representative_N1	FOXD2 (Forkhead)	73.86%	14.58%	11.57%	14152	Tethered binding less frequent than canonical binding but more frequent than co-binding
JUND (bZIP)	H1	JUND_rv3	PRDM15 (C2H2 ZF)	28.69%	66.05%	5.26%	11481	Tethered binding most frequent
JUND (bZIP)	K562	JUND_rv3	ZNF148 (C2H2 ZF)	55.01%	26.59%	18.40%	24465	Tethered binding less frequent than canonical binding but more frequent than co-binding
JUND (bZIP)	K562	JUND_rv3	ZNF281 (C2H2 ZF)	39.27%	49.77%	10.96%	46229	Tethered binding most frequent
KLF10 (C2H2 ZF)	HEK293	KLF10_rv3	NFYA (CBF/NF-Y)	85.23%	4.62%	10.15%	15047	Co-binding less frequent than canonical binding but more frequent than tethered binding
KLF16 (C2H2 ZF)	K562	KLF16_representative_N2	GATA2 (GATA)	70.68%	25.74%	3.57%	14415	Tethered binding less frequent than canonical binding but more frequent than co-binding
KLF16 (C2H2 ZF)	K562	KLF16_representative_N2	TWIST2 (bHLH)	100.00%	0.00%	0.00%	10704	not co-binding
KLF17 (C2H2 ZF)	HEK293	KLF17_HEK293_ENCSR065WUF_merged_N1	ATF3 (bZIP)	74.80%	13.84%	11.37%	25584	Tethered binding less frequent than canonical binding but more frequent than co-binding
KLF4 (C2H2 ZF)	MCF-7	KLF4_MCF-7_ENCSR265WJC_merged_N1	FOXC2 (Forkhead)	82.47%	7.40%	10.13%	4107	Co-binding less frequent than canonical binding but more frequent than tethered binding

LCORL (Pipsqueak)	HepG2	M01304_2.00 from Cis-BP	NFIA (SMAD)	12.62%	82.56%	4.82%	2678	Tethered binding most frequent
LEF1 (HMG/Sox)	K562	LEF1_K562_ENCSR832OGB_merged_N1	GATA3 (GATA)	35.49%	58.05%	6.46%	4751	Tethered binding most frequent
LEF1 (HMG/Sox)	K562	LEF1_K562_ENCSR832OGB_merged_N1	GATA5 (GATA)	66.18%	21.89%	11.93%	1425	Tethered binding less frequent than canonical binding but more frequent than co-binding
MAX (bHLH)	Ishikawa	MAX_rv3	E2F6 (E2F)	31.48%	59.94%	8.57%	21007	Tethered binding most frequent
MAX (bHLH)	SK-N-SH	MAX_rv3	ELK4 (Ets)	43.33%	51.43%	5.23%	14049	Tethered binding most frequent
MAX (bHLH)	HepG2	MAX_rv3	KLF12 (C2H2 ZF)	25.25%	59.96%	14.79%	16479	Tethered binding most frequent
MAX (bHLH)	HeLa-S3	MAX_rv3	FOSL2 (bZIP)	30.45%	65.28%	4.27%	17250	Tethered binding most frequent
MAX (bHLH)	liver	MAX_rv3	ELF4 (Ets)	47.17%	47.16%	5.67%	6016	Tethered binding less frequent than canonical binding but more frequent than co-binding
MAZ (C2H2 ZF)	HeLa-S3	MAZ_representative HSAv2_N2	PATZ1 (C2H2 ZF; AT h	10.51%	6.78%	82.71%	21083	Co-binding most frequent
MAZ (C2H2 ZF)	HepG2	MAZ_representative HSAv2_N2	PATZ1 (C2H2 ZF; AT h	3.47%	6.91%	89.63%	20000	Co-binding most frequent
MAZ (C2H2 ZF)	IMR-90	MAZ_representative HSAv2_N2	PATZ1 (C2H2 ZF; AT h	9.75%	10.16%	80.09%	42273	Co-binding most frequent
MAZ (C2H2 ZF)	K562	MAZ_representative HSAv2_N2	PATZ1 (C2H2 ZF; AT h	20.88%	10.33%	68.79%	40277	Co-binding most frequent
MAZ (C2H2 ZF)	HEK293	MAZ_representative HSAv2_N2	PATZ1 (C2H2 ZF; AT h	16.22%	5.22%	78.56%	24852	Co-binding most frequent
MAZ (C2H2 ZF)	K562	MAZ_representative HSAv2_N2	PATZ1 (C2H2 ZF; AT h	13.35%	5.59%	81.05%	11922	Co-binding most frequent
MAZ (C2H2 ZF)	HepG2	MAZ_representative HSAv2_N2	PATZ1 (C2H2 ZF; AT h	5.49%	7.55%	86.95%	12630	Co-binding most frequent

MBD2 (MBD)	K562	M09256_2.00 from Cis-BP	NFYB (Unknown)	86.49%	8.99%	4.53%	4618	Tethered binding less frequent than canonical binding but more frequent than co-binding
MBD2 (MBD)	K562	M09256_2.00 from Cis-BP	NFYA (CBF/NF-Y)	55.32%	33.83%	10.85%	6352	Tethered binding less frequent than canonical binding but more frequent than co-binding
MBD2 (MBD)	MCF-7	M09256_2.00 from Cis-BP	NFYA (CBF/NF-Y)	63.16%	28.88%	7.96%	7215	Tethered binding less frequent than canonical binding but more frequent than co-binding
MEF2A (MADS box)	GM12878	MEF2A_rv3	IRF1 (IRF)	34.54%	49.78%	15.67%	16463	Tethered binding most frequent
MEF2A (MADS box)	K562	MEF2A_rv3	JUND (bZIP)	68.20%	18.75%	13.05%	2752	Tethered binding less frequent than canonical binding but more frequent than co-binding
MEF2A (MADS box)	SK-N-SH	MEF2A_rv3	FOSL2 (bZIP)	38.68%	45.41%	15.91%	7293	Tethered binding most frequent
MEF2A (MADS box)	HepG2	MEF2A_rv3	FOXC2 (Forkhead)	21.31%	70.13%	8.57%	4623	Tethered binding most frequent
MEF2B (MADS box)	GM12878	MEF2B_GM12878_ENCSR177VFS_merged_N2	EBF1 (EBF1)	0.00%	100.00%	0.00%	13084	Tethered binding most frequent
MEF2D (MADS box)	HepG2	MEF2D_rv3	FOXC2 (Forkhead)	22.51%	68.96%	8.53%	3354	Tethered binding most frequent
MEIS2 (Homeodomain)	K562	MEIS2_rv3	GATA3 (GATA)	56.39%	37.07%	6.54%	12777	Tethered binding less frequent than canonical binding but more frequent than co-binding
MGA (T-box)	K562	M02822_2.00 from Cis-BP	MYC (bHLH)	9.32%	88.48%	2.20%	7786	Tethered binding most frequent

MGA (T-box)	HepG2	M02822_2.00 from Cis-BP	MYC (bHLH)	8.20%	89.82%	1.97%	3498	Tethered binding most frequent
MGA (T-box)	HepG2	M02822_2.00 from Cis-BP	MAX (bHLH)	8.76%	89.09%	2.15%	3263	Tethered binding most frequent
MITF (bHLH)	K562	MITF_representative_N1	GATA3 (GATA)	70.37%	22.09%	7.54%	2707	Tethered binding less frequent than canonical binding but more frequent than co-binding
MIXL1 (Homeodomain)	HepG2	M05361_2.00 from Cis-BP	FOXC2 (Forkhead)	6.23%	92.03%	1.73%	5598	Tethered binding most frequent
MIXL1 (Homeodomain)	HepG2	M05361_2.00 from Cis-BP	NR2F6 (Nuclear recepto	6.17%	92.48%	1.35%	5928	Tethered binding most frequent
MLX (bHLH)	HepG2	MLX_rv3	HNF4A (Nuclear recept	0.00%	100.00%	0.00%	8477	Tethered binding most frequent
MLX (bHLH)	HepG2	MLX_rv3	FOXC2 (Forkhead)	0.00%	100.00%	0.00%	5359	Tethered binding most frequent
MNT (bHLH)	HepG2	MNT_rv3	KLF12 (C2H2 ZF)	22.83%	65.41%	11.76%	13895	Tethered binding most frequent
MNT (bHLH)	HepG2	MNT_rv3	FOXF1 (Forkhead)	56.39%	40.29%	3.32%	8049	Tethered binding less frequent than canonical binding but more frequent than co-binding
MNT (bHLH)	K562	MNT_rv3	KLF10 (C2H2 ZF)	26.61%	56.75%	16.64%	10999	Tethered binding most frequent
MSX2 (Homeodomain)	MCF-7	M03127_2.00 from Cis-BP	ESR2 (Nuclear receptor	12.92%	84.58%	2.50%	240	Tethered binding most frequent
MSX2 (Homeodomain)	MCF-7	M03127_2.00 from Cis-BP	FOXC2 (Forkhead)	7.01%	88.22%	4.78%	314	Tethered binding most frequent
MXI1 (bHLH)	GM12878	MXI1_rv3	ELK4 (Ets)	39.33%	50.23%	10.44%	2367	Tethered binding most frequent
MXI1 (bHLH)	GM12878	MXI1_rv3	RFX3 (RFX)	48.57%	44.67%	6.76%	2129	Tethered binding less frequent than canonical binding but more frequent than co-binding

MXI1 (bHLH)	H1	MXI1_rv3	RFX3 (RFX)	48.79%	44.70%	6.51%	3841	Tethered binding less frequent than canonical binding but more frequent than co-binding
MXI1 (bHLH)	H1	MXI1_rv3	MEIS2 (Homeodomain)	100.00%	0.00%	0.00%	2124	not co-binding
MXI1 (bHLH)	HepG2	MXI1_rv3	ZBTB7A (C2H2 ZF)	32.34%	60.87%	6.79%	5829	Tethered binding most frequent
MXI1 (bHLH)	HepG2	MXI1_rv3	KLF12 (C2H2 ZF)	11.60%	78.19%	10.21%	10458	Tethered binding most frequent
MXI1 (bHLH)	K562	MXI1_rv3	RFX3 (RFX)	44.37%	50.72%	4.91%	3811	Tethered binding most frequent
MXI1 (bHLH)	SK-N-SH	MXI1_rv3	MEIS2 (Homeodomain)	100.00%	0.00%	0.00%	4811	not co-binding
MYBL2 (Myb/SANT)	HepG2	MYBL2_representati veHSAv2_N1	FOXC2 (Forkhead)	23.22%	68.45%	8.33%	4548	Tethered binding most frequent
MYBL2 (Myb/SANT)	HepG2	MYBL2_representati veHSAv2_N1	SP2 (C2H2 ZF)	17.13%	79.64%	3.24%	15847	Tethered binding most frequent
MYBL2 (Myb/SANT)	HepG2	MYBL2_representati veHSAv2_N1	NFYA (CBF/NF-Y)	46.10%	50.41%	3.49%	6508	Tethered binding most frequent
MYC (bHLH)	HepG2	MYC_rv3	ZNF281 (C2H2 ZF)	34.43%	41.60%	23.97%	2274	Tethered binding most frequent
MYC (bHLH)	endothelial-cell-	MYC_rv3	WT1 (C2H2 ZF)	22.97%	54.19%	22.85%	3379	Tethered binding most frequent
MYC (bHLH)	endothelial-cell-	MYC_rv3	ELF1 (Ets)	42.56%	42.56%	14.88%	2695	Tethered binding most frequent
MYC (bHLH)	MCF-7	MYC_rv3	ELK4 (Ets)	49.51%	45.18%	5.31%	20465	Tethered binding less frequent than canonical binding but more frequent than co-binding
MYC (bHLH)	MCF-7	MYC_rv3	WT1 (C2H2 ZF)	13.38%	75.52%	11.09%	27513	Tethered binding most frequent
MYC (bHLH)	MCF10A	MYC_rv3	FOS (bZIP)	25.44%	66.83%	7.73%	16469	Tethered binding most frequent
MYC (bHLH)	MCF10A	MYC_rv3	CEBPE (bZIP)	43.33%	51.36%	5.32%	11231	Tethered binding most frequent
MYC (bHLH)	MCF10A	MYC_rv3	PATZ1 (C2H2 ZF; AT h	26.08%	64.15%	9.77%	27154	Tethered binding most frequent

MYC (bHLH)	A549	MYC_rv3	ZNF281 (C2H2 ZF)	18.95%	62.10%	18.95%	7568	Tethered binding most frequent
MYC (bHLH)	K562	MYC_rv3	ELK4 (Ets)	49.91%	43.35%	6.74%	13826	Tethered binding less frequent than canonical binding but more frequent than co-binding
MYC (bHLH)	K562	MYC_rv3	ELK4 (Ets)	53.67%	38.77%	7.56%	7941	Tethered binding less frequent than canonical binding but more frequent than co-binding
MYC (bHLH)	NB4	MYC_rv3	ELK4 (Ets)	48.54%	34.95%	16.51%	3119	Tethered binding less frequent than canonical binding but more frequent than co-binding
MYC (bHLH)	HeLa-S3	MYC_rv3	FOSL2 (bZIP)	31.01%	63.69%	5.30%	13937	Tethered binding most frequent
MYC (bHLH)	K562	MYC_rv3	ELK4 (Ets)	58.29%	30.24%	11.47%	3426	Tethered binding less frequent than canonical binding but more frequent than co-binding
MYNN (C2H2 ZF)	HEK293	MYNN_representative_N1	RFX5 (RFX)	65.13%	31.28%	3.59%	2759	Tethered binding less frequent than canonical binding but more frequent than co-binding
MYNN (C2H2 ZF)	K562	MYNN_representative_N1	RFX5 (RFX)	50.27%	45.18%	4.55%	4774	Tethered binding less frequent than canonical binding but more frequent than co-binding
MYRF (Ndt80/PhoG)	HepG2	M02385_2.00 from Cis-BP	REST (C2H2 ZF)	0.77%	99.02%	0.21%	7234	Tethered binding most frequent
NANOG (Homeodomain)	H1	NANOG_representative_N1	POU5F1B (Homeodoma	12.24%	14.89%	72.87%	2835	Co-binding most frequent

NANOG (Homeodomain)	H1	NANOG_representative_N1	ZIC2 (C2H2 ZF)	38.43%	49.50%	12.08%	4778	Tethered binding most frequent
NANOG (Homeodomain)	H1	NANOG_representative_N1	SOX6 (HMG/Sox)	58.31%	26.72%	14.97%	3293	Tethered binding less frequent than canonical binding but more frequent than co-binding
NANOG (Homeodomain)	GM23338	NANOG_representative_N1	POU5F1B (Homeodoma	8.77%	12.19%	79.03%	6167	Co-binding most frequent
NCOA1 (bHLH)	K562	NCOA1_K562_ENCSR711SNW_merged_N1	GATA3 (GATA)	46.02%	49.44%	4.54%	5702	Tethered binding most frequent
NCOA1 (bHLH)	K562	NCOA1_K562_ENCSR711SNW_merged_N1	GATA2 (GATA)	37.91%	56.44%	5.65%	6619	Tethered binding most frequent
NCOA1 (bHLH)	K562	NCOA1_K562_ENCSR711SNW_merged_N1	ELK4 (Ets)	54.14%	42.94%	2.92%	3288	Tethered binding less frequent than canonical binding but more frequent than co-binding
NCOA3 (bHLH)	MCF-7	NCOA3_MCF-7_ENCSR573OJP_merged_N2	ESR2 (Nuclear receptor	31.87%	47.71%	20.42%	4775	Tethered binding most frequent
NEUROD1 (bHLH)	K562	M04148_2.00 from Cis-BP	NFYA (CBF/NF-Y)	14.77%	84.44%	0.80%	3765	Tethered binding most frequent
NEUROD1 (bHLH)	K562	M04148_2.00 from Cis-BP	SP2 (C2H2 ZF)	1.95%	97.31%	0.74%	21751	Tethered binding most frequent
NEUROD1 (bHLH)	K562	M04148_2.00 from Cis-BP	PRDM15 (C2H2 ZF)	13.37%	85.80%	0.82%	4128	Tethered binding most frequent
NFAT5 (Rel)	HepG2	M03447_2.00 from Cis-BP	REST (C2H2 ZF)	9.60%	87.20%	3.20%	250	Tethered binding most frequent
NFATC3 (Rel)	GM12878	NFATC3_rv3	SPI1 (Ets)	0.00%	100.00%	0.00%	9115	Tethered binding most frequent
NFIA (SMAD)	HepG2	NFIA_rv3	FOXC2 (Forkhead)	74.30%	18.91%	6.79%	38786	Tethered binding less frequent than canonical binding but more frequent than co-binding

NFIC (SMAD)	HepG2	NFIC_rv3	FOXC1 (Forkhead)	27.27%	63.75%	8.98%	6728	Tethered binding most frequent
NFIC (SMAD)	HepG2	NFIC_rv3	TCF7L2 (HMG/Sox)	41.73%	51.02%	7.25%	4980	Tethered binding most frequent
NFIC (SMAD)	GM12878	NFIC_rv3	FOS (bZIP)	17.96%	78.20%	3.84%	10647	Tethered binding most frequent
NFIC (SMAD)	GM12878	NFIC_rv3	IRF1 (IRF)	9.77%	86.01%	4.21%	16594	Tethered binding most frequent
NFIC (SMAD)	GM12878	NFIC_rv3	RUNX1 (Runt)	17.17%	78.47%	4.36%	10782	Tethered binding most frequent
NFIC (SMAD)	SK-N-SH	NFIC_rv3	JDP2 (bZIP)	56.45%	33.46%	10.09%	40462	Tethered binding less frequent than canonical binding but more frequent than co-binding
NFIC (SMAD)	Ishikawa	NFIC_rv3	ZIC3 (C2H2 ZF)	66.58%	15.02%	18.40%	34193	Co-binding less frequent than canonical binding but more frequent than tethered binding
NFYA (CBF/NF-Y)	HeLa-S3	NFYA_representativeHSAv2_N1	SP2 (C2H2 ZF)	30.54%	29.32%	40.14%	5805	Co-binding most frequent
NFYA (CBF/NF-Y)	HeLa-S3	NFYA_representativeHSAv2_N1	PKNOX1 (Homeodomai	77.75%	8.54%	13.71%	4486	Co-binding less frequent than canonical binding but more frequent than tethered binding
NFYA (CBF/NF-Y)	HepG2	NFYA_representativeHSAv2_N1	SP2 (C2H2 ZF)	21.37%	33.99%	44.65%	4849	Co-binding most frequent
NFYA (CBF/NF-Y)	K562	NFYA_representativeHSAv2_N1	SP2 (C2H2 ZF)	34.30%	10.93%	54.77%	4134	Co-binding most frequent
NFYB (Unknown)	GM12878	NFYB_representativeHSAv2_N2	NFYA (CBF/NF-Y)	0.75%	85.75%	13.49%	6374	Tethered binding most frequent
NFYB (Unknown)	HeLa-S3	NFYB_representativeHSAv2_N2	NFYA (CBF/NF-Y)	0.77%	86.74%	12.50%	6786	Tethered binding most frequent

NFYB (Unknown)	HeLa-S3	NFYB_representativeHSAv2_N2	SP2 (C2H2 ZF)	8.27%	77.65%	14.08%	4027	Tethered binding most frequent
NFYB (Unknown)	K562	NFYB_representativeHSAv2_N2	NFYA (CBF/NF-Y)	0.69%	88.60%	10.72%	13867	Tethered binding most frequent
NFYB (Unknown)	K562	NFYB_representativeHSAv2_N2	NFYA (CBF/NF-Y)	1.21%	88.64%	10.15%	13914	Tethered binding most frequent
NFYB (Unknown)	WTC11	NFYB_representativeHSAv2_N2	NFYA (CBF/NF-Y)	0.91%	82.69%	16.40%	4750	Tethered binding most frequent
NFYB (Unknown)	WTC11	NFYB_representativeHSAv2_N2	SP2 (C2H2 ZF)	5.86%	78.77%	15.37%	3871	Tethered binding most frequent
NFYB (Unknown)	HepG2	NFYB_representativeHSAv2_N2	NFYA (CBF/NF-Y)	1.34%	83.88%	14.78%	8505	Tethered binding most frequent
NFYB (Unknown)	HepG2	NFYB_representativeHSAv2_N2	NFYA (CBF/NF-Y)	1.85%	85.01%	13.14%	9147	Tethered binding most frequent
NFYB (Unknown)	HepG2	NFYB_representativeHSAv2_N2	SP2 (C2H2 ZF)	5.07%	87.87%	7.06%	11303	Tethered binding most frequent
NFYC (Unknown)	HepG2	NFYC_HepG2_ENCSR569ARC_merged_N2	NFYA (CBF/NF-Y)	1.73%	85.26%	13.01%	10577	Tethered binding most frequent
NFYC (Unknown)	HepG2	NFYC_HepG2_ENCSR569ARC_merged_N2	SP2 (C2H2 ZF)	5.53%	88.62%	5.85%	13702	Tethered binding most frequent
NKX3-1 (Homeodomain)	HepG2	NKX3-1_HepG2_ENCSR647CXR_merged_N2	REST (C2H2 ZF)	70.48%	8.29%	21.23%	3005	Co-binding less frequent than canonical binding but more frequent than tethered binding
NKX3-1 (Homeodomain)	HepG2	NKX3-1_HepG2_ENCSR647CXR_merged_N2	ZNF384 (C2H2 ZF)	93.32%	6.03%	0.65%	2933	Tethered binding less frequent than canonical binding but more frequent than co-binding
NR2C1 (Nuclear receptor)	K562	NR2C1_representative_N2	ELF2 (Ets)	65.09%	26.08%	8.83%	9661	Tethered binding less frequent than canonical binding but more frequent than co-binding

NR2C1 (Nuclear receptor)	K562	NR2C1_representative_N2	ELF4 (Ets)	64.55%	26.22%	9.22%	7417	Tethered binding less frequent than canonical binding but more frequent than co-binding
NR2C1 (Nuclear receptor)	GM12878	NR2C1_representative_N2	ELK4 (Ets)	55.55%	32.84%	11.61%	5804	Tethered binding less frequent than canonical binding but more frequent than co-binding
NR2C1 (Nuclear receptor)	GM12878	NR2C1_representative_N2	ZBTB7A (C2H2 ZF)	57.42%	32.70%	9.88%	5792	Tethered binding less frequent than canonical binding but more frequent than co-binding
NR2C2 (Nuclear receptor)	HeLa-S3	NR2C2_rv3	ELF2 (Ets)	39.66%	44.86%	15.48%	1460	Tethered binding most frequent
NR2C2 (Nuclear receptor)	HepG2	NR2C2_rv3	ELF2 (Ets)	41.43%	43.19%	15.38%	1938	Tethered binding most frequent
NR2C2 (Nuclear receptor)	HepG2	NR2C2_rv3	ELF1 (Ets)	45.56%	40.42%	14.02%	1848	Tethered binding less frequent than canonical binding but more frequent than co-binding
NR2C2 (Nuclear receptor)	K562	NR2C2_rv3	ELF2 (Ets)	35.64%	43.92%	20.44%	362	Tethered binding most frequent
NR2C2 (Nuclear receptor)	WTC11	NR2C2_rv3	ELF2 (Ets)	31.87%	48.17%	19.96%	546	Tethered binding most frequent
NR2C2 (Nuclear receptor)	K562	NR2C2_rv3	ELF2 (Ets)	46.79%	37.71%	15.50%	1400	Tethered binding less frequent than canonical binding but more frequent than co-binding
NR2C2 (Nuclear receptor)	K562	NR2C2_rv3	KLF12 (C2H2 ZF)	33.87%	39.36%	26.77%	1438	Tethered binding most frequent

NR2C2 (Nuclear receptor)	HepG2	NR2C2_rv3	ELF1 (Ets)	59.27%	31.04%	9.69%	10330	Tethered binding less frequent than canonical binding but more frequent than co-binding
NR2C2 (Nuclear receptor)	HepG2	NR2C2_rv3	KLF15 (C2H2 ZF)	39.14%	43.66%	17.20%	12645	Tethered binding most frequent
NR2C2 (Nuclear receptor)	K562	NR2C2_rv3	ELF2 (Ets)	58.87%	32.45%	8.69%	15037	Tethered binding less frequent than canonical binding but more frequent than co-binding
NR2F1 (Nuclear receptor)	HepG2	NR2F1_representative_N1	FOXC2 (Forkhead)	67.45%	12.42%	20.13%	596	Co-binding less frequent than canonical binding but more frequent than tethered binding
NR2F1 (Nuclear receptor)	K562	NR2F1_representative_N1	GATA2 (GATA)	77.90%	14.77%	7.32%	17591	Tethered binding less frequent than canonical binding but more frequent than co-binding
NR2F2 (Nuclear receptor)	K562	NR2F2_representative_N1	GATA2 (GATA)	54.52%	36.62%	8.86%	8037	Tethered binding less frequent than canonical binding but more frequent than co-binding
NR2F2 (Nuclear receptor)	K562	NR2F2_representative_N1	NEUROD2 (bHLH)	55.20%	33.60%	11.20%	7672	Tethered binding less frequent than canonical binding but more frequent than co-binding
NR2F2 (Nuclear receptor)	MCF-7	NR2F2_representative_N1	FOXC2 (Forkhead)	53.27%	37.08%	9.66%	15214	Tethered binding less frequent than canonical binding but more frequent than co-binding

NR2F2 (Nuclear receptor)	HepG2	NR2F2_representative_N1	FOXC2 (Forkhead)	58.31%	29.19%	12.49%	8557	Tethered binding less frequent than canonical binding but more frequent than co-binding
NR2F2 (Nuclear receptor)	HepG2	NR2F2_representative_N1	TCF7L2 (HMG/Sox)	71.49%	17.87%	10.64%	7377	Tethered binding less frequent than canonical binding but more frequent than co-binding
NR2F2 (Nuclear receptor)	liver	NR2F2_representative_N1	IKZF1 (C2H2 ZF)	53.85%	31.68%	14.48%	19226	Tethered binding less frequent than canonical binding but more frequent than co-binding
NR2F2 (Nuclear receptor)	liver	NR2F2_representative_N1	EHF (Ets)	64.07%	24.29%	11.64%	13017	Tethered binding less frequent than canonical binding but more frequent than co-binding
NR2F6 (Nuclear receptor)	HepG2	NR2F6_representative_N1	FOXC2 (Forkhead)	61.56%	26.85%	11.59%	16347	Tethered binding less frequent than canonical binding but more frequent than co-binding
NR2F6 (Nuclear receptor)	K562	NR2F6_representative_N1	GATA3 (GATA)	68.41%	22.94%	8.66%	11236	Tethered binding less frequent than canonical binding but more frequent than co-binding
NR2F6 (Nuclear receptor)	HepG2	NR2F6_representative_N1	FOXD2 (Forkhead)	69.55%	21.59%	8.87%	15487	Tethered binding less frequent than canonical binding but more frequent than co-binding
NR3C1 (Nuclear receptor)	A549	NR3C1_representative_N1	BNC2 (C2H2 ZF)	35.56%	51.64%	12.79%	852	Tethered binding most frequent

NR3C1 (Nuclear receptor)	A549	NR3C1_representative_N1	FOXC2 (Forkhead)	56.62%	35.02%	8.36%	634	Tethered binding less frequent than canonical binding but more frequent than co-binding
NR3C1 (Nuclear receptor)	A549	NR3C1_representative_N1	ATF3 (bZIP)	47.86%	41.09%	11.05%	7152	Tethered binding less frequent than canonical binding but more frequent than co-binding
NR3C1 (Nuclear receptor)	A549	NR3C1_representative_N1	FOSL2 (bZIP)	66.78%	17.04%	16.17%	575	Tethered binding less frequent than canonical binding but more frequent than co-binding
NR3C1 (Nuclear receptor)	A549	NR3C1_representative_N1	CEBPD (bZIP)	66.16%	18.88%	14.97%	588	Tethered binding less frequent than canonical binding but more frequent than co-binding
NR3C1 (Nuclear receptor)	A549	NR3C1_representative_N1	CEBPA (bZIP)	60.67%	30.06%	9.27%	3084	Tethered binding less frequent than canonical binding but more frequent than co-binding
NR3C1 (Nuclear receptor)	A549	NR3C1_representative_N1	FOS (bZIP)	50.48%	40.38%	9.14%	16392	Tethered binding less frequent than canonical binding but more frequent than co-binding
NR3C1 (Nuclear receptor)	A549	NR3C1_representative_N1	ATF3 (bZIP)	42.43%	48.59%	8.98%	15191	Tethered binding most frequent
NR3C1 (Nuclear receptor)	A549	NR3C1_representative_N1	ATF3 (bZIP)	52.12%	35.61%	12.26%	3083	Tethered binding less frequent than canonical binding but more frequent than co-binding

NR3C1 (Nuclear receptor)	A549	NR3C1_representative_N1	CEBPD (bZIP)	58.17%	32.33%	9.50%	2601	Tethered binding less frequent than canonical binding but more frequent than co-binding
NR3C1 (Nuclear receptor)	A549	NR3C1_representative_N1	CEBPD (bZIP)	67.67%	21.02%	11.31%	1556	Tethered binding less frequent than canonical binding but more frequent than co-binding
NR3C1 (Nuclear receptor)	A549	NR3C1_representative_N1	BACH1 (bZIP)	33.26%	57.28%	9.46%	8184	Tethered binding most frequent
NRF1 (Unknown)	H1	NRF1_representative_N1	KLF12 (C2H2 ZF)	31.30%	6.37%	62.33%	4255	Co-binding most frequent
NRF1 (Unknown)	K562	NRF1_representative_N1	PATZ1 (C2H2 ZF; AT h	36.33%	10.02%	53.65%	3862	Co-binding most frequent
NRF1 (Unknown)	SK-N-SH	NRF1_representative_N1	KLF15 (C2H2 ZF)	22.75%	16.41%	60.84%	6769	Co-binding most frequent
NRF1 (Unknown)	K562	NRF1_representative_N1	SP4 (C2H2 ZF)	32.98%	34.06%	32.97%	14432	Tethered binding most frequent
NRF1 (Unknown)	HepG2	NRF1_representative_N1	SP2 (C2H2 ZF)	33.43%	26.27%	40.30%	13873	Co-binding most frequent
PATZ1 (C2H2 ZF; AT hook)	HepG2	PATZ1_representativeHSAv2_N1	SP5 (C2H2 ZF)	44.93%	12.68%	42.39%	47103	Co-binding less frequent than canonical binding but more frequent than tethered binding
PATZ1 (C2H2 ZF; AT hook)	HepG2	PATZ1_representativeHSAv2_N1	FOXC2 (Forkhead)	81.31%	11.61%	7.08%	46531	Tethered binding less frequent than canonical binding but more frequent than co-binding
PATZ1 (C2H2 ZF; AT hook)	HEK293	PATZ1_representativeHSAv2_N1	EGR1 (C2H2 ZF)	12.46%	19.82%	67.72%	40362	Co-binding most frequent

PAX5 (Paired box)	GM12878	PAX5_representative_N1	SPIB (Ets)	49.76%	42.87%	7.36%	23822	Tethered binding less frequent than canonical binding but more frequent than co-binding
PBX1 (Homeodomain)	A549	PBX1_A549_ENCSR637RKG_merged_N3	NFYA (CBF/NF-Y)	54.41%	24.35%	21.24%	2632	Tethered binding less frequent than canonical binding but more frequent than co-binding
PBX1 (Homeodomain)	A549	PBX1_A549_ENCSR637RKG_merged_N3	NFYB (Unknown)	60.75%	23.54%	15.71%	2604	Tethered binding less frequent than canonical binding but more frequent than co-binding
PBX1 (Homeodomain)	A549	PBX1_A549_ENCSR637RKG_merged_N3	SP2 (C2H2 ZF)	35.86%	36.02%	28.12%	3112	Tethered binding most frequent
PBX2 (Homeodomain)	K562	PBX2_representative_N2	NFYA (CBF/NF-Y)	69.41%	21.36%	9.24%	6410	Tethered binding less frequent than canonical binding but more frequent than co-binding
PBX2 (Homeodomain)	K562	PBX2_representative_N2	SP2 (C2H2 ZF)	36.92%	47.61%	15.47%	9622	Tethered binding most frequent
PBX2 (Homeodomain)	K562	PBX2_representative_N2	NFYA (CBF/NF-Y)	60.45%	17.15%	22.39%	2157	Co-binding less frequent than canonical binding but more frequent than tethered binding
PBX2 (Homeodomain)	K562	PBX2_representative_N2	SP2 (C2H2 ZF)	34.37%	29.73%	35.90%	2543	Co-binding most frequent
PBX2 (Homeodomain)	HepG2	PBX2_representative_N2	NFYA (CBF/NF-Y)	63.66%	18.72%	17.63%	3200	Tethered binding less frequent than canonical binding but more frequent than co-binding

PBX2 (Homeodomain)	HepG2	PBX2_representative_N2	SP2 (C2H2 ZF)	46.27%	29.49%	24.23%	3689	Tethered binding less frequent than canonical binding but more frequent than co-binding
PBX3 (Homeodomain)	GM12878	PBX3_representativeHSAv2_N1	NFYB (Unknown)	70.18%	16.84%	12.98%	4900	Tethered binding less frequent than canonical binding but more frequent than co-binding
PBX3 (Homeodomain)	GM12878	PBX3_representativeHSAv2_N1	SP2 (C2H2 ZF)	44.76%	32.32%	22.92%	6021	Tethered binding less frequent than canonical binding but more frequent than co-binding
PBX3 (Homeodomain)	A549	PBX3_representativeHSAv2_N1	NFYA (CBF/NF-Y)	50.07%	29.92%	20.02%	2293	Tethered binding less frequent than canonical binding but more frequent than co-binding
PBX3 (Homeodomain)	A549	PBX3_representativeHSAv2_N1	SP2 (C2H2 ZF)	17.77%	59.96%	22.28%	4013	Tethered binding most frequent
PBX3 (Homeodomain)	SK-N-SH	PBX3_representativeHSAv2_N1	NFYB (Unknown)	52.36%	34.87%	12.76%	8651	Tethered binding less frequent than canonical binding but more frequent than co-binding
PBX3 (Homeodomain)	SK-N-SH	PBX3_representativeHSAv2_N1	JUNB (bZIP)	30.54%	65.36%	4.10%	16263	Tethered binding most frequent
PBX3 (Homeodomain)	HEK293	PBX3_representativeHSAv2_N1	NFYA (CBF/NF-Y)	57.05%	24.96%	17.99%	1907	Tethered binding less frequent than canonical binding but more frequent than co-binding
PBX3 (Homeodomain)	HEK293	PBX3_representativeHSAv2_N1	SP2 (C2H2 ZF)	6.67%	73.64%	19.69%	5429	Tethered binding most frequent
PITX1 (Homeodomain)	HepG2	M04931_2.00 from Cis-BP	FOXD3 (Forkhead)	33.69%	61.21%	5.10%	3336	Tethered binding most frequent

PKNOX1 (Homeodomain)	K562	PKNOX1_representative_N2	NFYA (CBF/NF-Y)	78.96%	13.13%	7.91%	17985	Tethered binding less frequent than canonical binding but more frequent than co-binding
PKNOX1 (Homeodomain)	K562	PKNOX1_representative_N2	SP2 (C2H2 ZF)	57.95%	28.97%	13.08%	21995	Tethered binding less frequent than canonical binding but more frequent than co-binding
PKNOX1 (Homeodomain)	HEK293T	PKNOX1_representative_N2	NFYB (Unknown)	78.87%	12.88%	8.25%	14892	Tethered binding less frequent than canonical binding but more frequent than co-binding
PKNOX1 (Homeodomain)	HEK293T	PKNOX1_representative_N2	SP2 (C2H2 ZF)	68.17%	19.43%	12.40%	16102	Tethered binding less frequent than canonical binding but more frequent than co-binding
PKNOX1 (Homeodomain)	GM12878	PKNOX1_representative_N2	NFYA (CBF/NF-Y)	78.38%	12.97%	8.65%	13611	Tethered binding less frequent than canonical binding but more frequent than co-binding
PKNOX1 (Homeodomain)	GM12878	PKNOX1_representative_N2	SP2 (C2H2 ZF)	59.22%	27.47%	13.31%	16331	Tethered binding less frequent than canonical binding but more frequent than co-binding
PKNOX1 (Homeodomain)	MCF-7	PKNOX1_representative_N2	NFYB (Unknown)	78.93%	12.11%	8.96%	13886	Tethered binding less frequent than canonical binding but more frequent than co-binding

PKNOX1 (Homeodomain)	MCF-7	PKNOX1_representative_N2	SP2 (C2H2 ZF)	57.70%	25.92%	16.38%	16474	Tethered binding less frequent than canonical binding but more frequent than co-binding
PPARG (Nuclear receptor)	HepG2	PPARG_HepG2_ENCSR130VQL_merged_N1	FOXC2 (Forkhead)	55.14%	31.94%	12.92%	10696	Tethered binding less frequent than canonical binding but more frequent than co-binding
PRDM1 (C2H2 ZF)	HeLa-S3	PRDM1_representative_N1	FOSL2 (bZIP)	68.45%	8.67%	22.88%	3540	Co-binding less frequent than canonical binding but more frequent than tethered binding
PRDM1 (C2H2 ZF)	HEK293	PRDM1_representative_N1	IRF3 (IRF)	21.68%	29.67%	48.65%	66301	Co-binding most frequent
PRDM1 (C2H2 ZF)	A549	PRDM1_representative_N1	FOSL2 (bZIP)	70.23%	13.61%	16.16%	3211	Co-binding less frequent than canonical binding but more frequent than tethered binding
PRRX2 (Homeodomain)	WTC11	M05263_2.00 from Cis-BP	ZNF460 (C2H2 ZF)	2.83%	96.11%	1.06%	283	Tethered binding most frequent
RARA (Nuclear receptor)	HepG2	RARA_HepG2_ENCSR500WXT_merged_N1	FOXC2 (Forkhead)	52.52%	36.83%	10.64%	18793	Tethered binding less frequent than canonical binding but more frequent than co-binding
RBPJ (CSL)	HepG2	RBPJ_rv3	HNF4A (Nuclear recepto	37.68%	53.17%	9.15%	26088	Tethered binding most frequent
RELA (Rel)	GM19099	RELA_representativeHSAv2_N1	SPIB (Ets)	53.11%	31.89%	15.00%	11007	Tethered binding less frequent than canonical binding but more frequent than co-binding

RELA (Rel)	HepG2	RELA_representativeHSAv2_N1	PATZ1 (C2H2 ZF; AT h	6.83%	83.56%	9.61%	16363	Tethered binding most frequent
RELA (Rel)	WTC11	RELA_representativeHSAv2_N1	BHLHE40 (bHLH)	24.48%	69.60%	5.92%	625	Tethered binding most frequent
RELA (Rel)	WTC11	RELA_representativeHSAv2_N1	REST (C2H2 ZF)	18.60%	77.49%	3.91%	844	Tethered binding most frequent
RFX1 (RFX)	MCF-7	RFX1_representative_N1	SP4 (C2H2 ZF)	64.69%	18.47%	16.84%	17947	Tethered binding less frequent than canonical binding but more frequent than co-binding
RFX5 (RFX)	GM12878	RFX5_representative_N2	NFYA (CBF/NF-Y)	31.28%	52.52%	16.20%	2679	Tethered binding most frequent
RFX5 (RFX)	HeLa-S3	RFX5_representative_N2	NFYA (CBF/NF-Y)	48.65%	42.75%	8.60%	6919	Tethered binding less frequent than canonical binding but more frequent than co-binding
RFX5 (RFX)	HepG2	RFX5_representative_N2	NFYA (CBF/NF-Y)	49.29%	39.26%	11.45%	5102	Tethered binding less frequent than canonical binding but more frequent than co-binding
RFX5 (RFX)	SK-N-SH	RFX5_representative_N2	NFYA (CBF/NF-Y)	41.84%	43.61%	14.55%	3781	Tethered binding most frequent
RFX5 (RFX)	A549	RFX5_representative_N2	NFYA (CBF/NF-Y)	45.45%	39.07%	15.48%	2022	Tethered binding less frequent than canonical binding but more frequent than co-binding
RFX5 (RFX)	MCF-7	RFX5_representative_N2	NFYA (CBF/NF-Y)	55.32%	32.54%	12.14%	5998	Tethered binding less frequent than canonical binding but more frequent than co-binding

RREB1 (C2H2 ZF)	HepG2	RREB1_representativeHSAv2_N3	FOXC2 (Forkhead)	55.82%	37.03%	7.16%	16848	Tethered binding less frequent than canonical binding but more frequent than co-binding
RUNX1 (Runt)	K562	RUNX1_rv3	GATA5 (GATA)	41.10%	46.71%	12.19%	1124	Tethered binding most frequent
RUNX3 (Runt)	GM12878	RUNX3_GM12878_ENCSR000BRI_merged_N2	IRF1 (IRF)	44.90%	42.54%	12.56%	50411	Tethered binding less frequent than canonical binding but more frequent than co-binding
RXRA (Nuclear receptor)	HepG2	RXRA_representative_N1	FOXC2 (Forkhead)	32.92%	59.60%	7.48%	6373	Tethered binding most frequent
RXRA (Nuclear receptor)	SK-N-SH	RXRA_representative_N1	FOS (bZIP)	14.60%	76.08%	9.33%	7482	Tethered binding most frequent
RXRA (Nuclear receptor)	HepG2	RXRA_representative_N1	FOXC2 (Forkhead)	69.94%	26.52%	3.53%	35248	Tethered binding less frequent than canonical binding but more frequent than co-binding
RXRB (Nuclear receptor)	HepG2	RXRB_HepG2_ENCSR560SEP_merged_N1	FOXC2 (Forkhead)	52.38%	36.91%	10.71%	15603	Tethered binding less frequent than canonical binding but more frequent than co-binding
SALL1 (C2H2 ZF)	HepG2	SALL1_HepG2_ENCSR407MQT_merged_N1	FOXC2 (Forkhead)	63.95%	27.25%	8.80%	19828	Tethered binding less frequent than canonical binding but more frequent than co-binding
SETDB1 (MBD)	HEK293	SETDB1_HEK293_ENCSR348AGV_merged_N1	ZNF75D (C2H2 ZF)	40.29%	53.73%	5.98%	8196	Tethered binding most frequent
SIX1 (Homeodomain)	HepG2	SIX1_HepG2_ENCSR561BQM_merged_N1	FOXC1 (Forkhead)	36.21%	55.68%	8.12%	9647	Tethered binding most frequent

SIX4 (Homeodomain)	WTC11	SIX4_rv3	PATZ1 (C2H2 ZF; AT h	8.87%	83.72%	7.41%	2052	Tethered binding most frequent
SIX4 (Homeodomain)	WTC11	SIX4_rv3	ZNF32 (C2H2 ZF)	13.01%	66.57%	20.42%	999	Tethered binding most frequent
SIX4 (Homeodomain)	HepG2	SIX4_rv3	ZNF320 (C2H2 ZF)	7.23%	86.96%	5.81%	14103	Tethered binding most frequent
SIX4 (Homeodomain)	HepG2	SIX4_rv3	FOXC2 (Forkhead)	27.08%	68.13%	4.78%	5771	Tethered binding most frequent
SIX5 (Homeodomain)	K562	M08208_2.00 from Cis-BP	THAP11 (THAP finger)	2.57%	19.75%	77.68%	1792	Co-binding most frequent
SIX5 (Homeodomain)	K562	M08208_2.00 from Cis-BP	THAP11 (THAP finger)	2.08%	25.22%	72.70%	1923	Co-binding most frequent
SIX5 (Homeodomain)	H1	M08208_2.00 from Cis-BP	THAP11 (THAP finger)	1.02%	21.42%	77.56%	2255	Co-binding most frequent
SIX5 (Homeodomain)	H1	M08208_2.00 from Cis-BP	THAP11 (THAP finger)	1.31%	16.81%	81.88%	2130	Co-binding most frequent
SIX5 (Homeodomain)	GM12878	M08208_2.00 from Cis-BP	THAP11 (THAP finger)	2.42%	34.50%	63.08%	2484	Co-binding most frequent
SIX5 (Homeodomain)	GM12878	M08208_2.00 from Cis-BP	THAP11 (THAP finger)	2.88%	29.04%	68.08%	2293	Co-binding most frequent
SIX5 (Homeodomain)	K562	M08208_2.00 from Cis-BP	ZNF76 (C2H2 ZF)	9.62%	29.37%	61.01%	2111	Co-binding most frequent
SIX5 (Homeodomain)	K562	M08208_2.00 from Cis-BP	THAP11 (THAP finger)	1.81%	20.44%	77.75%	1874	Co-binding most frequent
SIX5 (Homeodomain)	K562	M08208_2.00 from Cis-BP	THAP11 (THAP finger)	2.07%	18.70%	79.23%	1834	Co-binding most frequent
SIX5 (Homeodomain)	A549	M08208_2.00 from Cis-BP	THAP11 (THAP finger)	4.17%	57.22%	38.61%	3142	Tethered binding most frequent
SIX5 (Homeodomain)	A549	M08208_2.00 from Cis-BP	THAP11 (THAP finger)	6.77%	37.25%	55.98%	2142	Co-binding most frequent
SKI (Unknown)	HepG2	SKI_HepG2_ENCSR754MUD_merged_N2	FOXC2 (Forkhead)	27.89%	66.88%	5.22%	11182	Tethered binding most frequent
SKI (Unknown)	HepG2	SKI_HepG2_ENCSR754MUD_merged_N2	NR6A1 (Nuclear recepto	26.79%	68.20%	5.01%	11645	Tethered binding most frequent

SMAD3 (SMAD)	HepG2	SMAD3_K562_ENCSR376XAV_merged_N1	REST (C2H2 ZF)	28.66%	67.08%	4.26%	9117	Tethered binding most frequent
SMAD3 (SMAD)	HepG2	SMAD3_K562_ENCSR376XAV_merged_N1	FOXC2 (Forkhead)	31.66%	64.27%	4.07%	8399	Tethered binding most frequent
SMAD4 (SMAD)	HepG2	MA1153.1 from JASPAR	NR5A1 (Nuclear recepto	14.89%	81.48%	3.63%	31383	Tethered binding most frequent
SMAD4 (SMAD)	HepG2	MA1153.1 from JASPAR	FOXC2 (Forkhead)	26.54%	70.05%	3.41%	19403	Tethered binding most frequent
SMAD5 (SMAD)	K562	SMAD5_K562_ENCSR000FCD_merged_N1	ZNF93 (C2H2 ZF)	13.80%	75.51%	10.69%	22563	Tethered binding most frequent
SOX13 (HMG/Sox)	HepG2	SOX13_representative_N1	FOXC2 (Forkhead)	31.37%	56.50%	12.13%	4370	Tethered binding most frequent
SOX13 (HMG/Sox)	HepG2	SOX13_representative_N1	FOXC2 (Forkhead)	29.85%	60.52%	9.63%	16207	Tethered binding most frequent
SOX5 (HMG/Sox)	HepG2	SOX5_HepG2_ENCSR961WLZ_merged_N1	FOXC2 (Forkhead)	29.13%	61.77%	9.10%	12720	Tethered binding most frequent
SOX6 (HMG/Sox)	HepG2	SOX6_representativeHSAv2_N1	FOXC2 (Forkhead)	36.86%	47.87%	15.27%	12132	Tethered binding most frequent
SOX6 (HMG/Sox)	HepG2	SOX6_representativeHSAv2_N1	NR2F6 (Nuclear recepto	31.88%	52.01%	16.11%	13180	Tethered binding most frequent
SOX6 (HMG/Sox)	K562	SOX6_representativeHSAv2_N1	GATA2 (GATA)	30.86%	59.53%	9.61%	11283	Tethered binding most frequent
SP1 (C2H2 ZF)	GM12878	SP1_representative_N3	NFYB (Unknown)	94.43%	1.64%	3.93%	15962	Co-binding less frequent than canonical binding but more frequent than tethered binding
SP1 (C2H2 ZF)	GM12878	SP1_representative_N3	NFYA (CBF/NF-Y)	75.18%	10.54%	14.28%	17551	Co-binding less frequent than canonical binding but more frequent than tethered binding

SP1 (C2H2 ZF)	H1-hESC	SP1_representative_N3	TLX3 (Homeodomain)	80.00%	8.63%	11.37%	15443	Co-binding less frequent than canonical binding but more frequent than tethered binding
SP1 (C2H2 ZF)	H1-hESC	SP1_representative_N3	NFYA (CBF/NF-Y)	68.88%	14.41%	16.71%	16485	Co-binding less frequent than canonical binding but more frequent than tethered binding
SP1 (C2H2 ZF)	K562	SP1_representative_N3	NFYA (CBF/NF-Y)	49.84%	12.94%	37.22%	3834	Co-binding less frequent than canonical binding but more frequent than tethered binding
SP1 (C2H2 ZF)	K562	SP1_representative_N3	NFYA (CBF/NF-Y)	54.30%	12.02%	33.68%	3794	Co-binding less frequent than canonical binding but more frequent than tethered binding
SP1 (C2H2 ZF)	A549	SP1_representative_N3	NFYA (CBF/NF-Y)	74.27%	12.01%	13.72%	16948	Co-binding less frequent than canonical binding but more frequent than tethered binding
SP1 (C2H2 ZF)	HCT116	SP1_representative_N3	FOS (bZIP)	32.83%	58.65%	8.53%	22059	Tethered binding most frequent
SP1 (C2H2 ZF)	liver	SP1_representative_N3	HNF4A (Nuclear recept	42.87%	50.56%	6.58%	43115	Tethered binding most frequent

SP1 (C2H2 ZF)	liver	SP1_representative_N3	CEBPD (bZIP)	63.05%	33.82%	3.13%	32212	Tethered binding less frequent than canonical binding but more frequent than co-binding
SP1 (C2H2 ZF)	liver	SP1_representative_N3	FOXD2 (Forkhead)	55.40%	40.85%	3.75%	36039	Tethered binding less frequent than canonical binding but more frequent than co-binding
SP1 (C2H2 ZF)	liver	SP1_representative_N3	HNF4A (Nuclear recept[illegible]	40.61%	52.06%	7.33%	37498	Tethered binding most frequent
SP1 (C2H2 ZF)	HepG2	SP1_representative_N3	NFYA (CBF/NF-Y)	83.61%	4.13%	12.26%	16519	Co-binding less frequent than canonical binding but more frequent than tethered binding
SP1 (C2H2 ZF)	K562	SP1_representative_N3	NFYB (Unknown)	94.43%	1.64%	3.93%	15962	Co-binding less frequent than canonical binding but more frequent than tethered binding
SP1 (C2H2 ZF)	K562	SP1_representative_N3	NFYA (CBF/NF-Y)	75.18%	10.54%	14.28%	17551	Co-binding less frequent than canonical binding but more frequent than tethered binding
SP2 (C2H2 ZF)	K562	SP2_representative HSAv2_N1	NFYA (CBF/NF-Y)	68.26%	17.51%	14.24%	14372	Tethered binding less frequent than canonical binding but more frequent than co-binding
SP2 (C2H2 ZF)	HepG2	SP2_representative HSAv2_N1	NFYA (CBF/NF-Y)	33.74%	12.30%	53.96%	821	Co-binding most frequent

SP2 (C2H2 ZF)	H1-hESC	SP2_representative HSAv2_N1	NFYA (CBF/NF-Y)	40.93%	15.17%	43.91%	1813	Co-binding most frequent
SP2 (C2H2 ZF)	HEK293	SP2_representative HSAv2_N1	NFYA (CBF/NF-Y)	76.12%	9.33%	14.56%	19092	Co-binding less frequent than canonical binding but more frequent than tethered binding
SP2 (C2H2 ZF)	HEK293	SP2_representative HSAv2_N1	NFYA (CBF/NF-Y)	79.11%	8.08%	12.81%	18832	Co-binding less frequent than canonical binding but more frequent than tethered binding
SP2 (C2H2 ZF)	HepG2	SP2_representative HSAv2_N1	NFYA (CBF/NF-Y)	44.59%	11.03%	44.38%	3770	Co-binding less frequent than canonical binding but more frequent than tethered binding
SP2 (C2H2 ZF)	HepG2	SP2_representative HSAv2_N1	PKNOX1 (Homeodomai	68.29%	6.94%	24.78%	3604	Co-binding less frequent than canonical binding but more frequent than tethered binding
SP2 (C2H2 ZF)	HepG2	SP2_representative HSAv2_N1	NFYB (Unknown)	56.90%	8.39%	34.72%	3661	Co-binding less frequent than canonical binding but more frequent than tethered binding
SP4 (C2H2 ZF)	H1-hESC	SP4_H1-hESC_ENCSR000BQV_merged_N1	TFE3 (bHLH)	82.43%	12.06%	5.52%	16615	Tethered binding less frequent than canonical binding but more frequent than co-binding

SP5 (C2H2 ZF)	HepG2	SP5_HepG2_ENCSR019NPF_merged_N1	FOXC2 (Forkhead)	77.70%	13.58%	8.72%	32653	Tethered binding less frequent than canonical binding but more frequent than co-binding
SRF (MADS box)	GM12878	SRF_representative_N1	ELF1 (Ets)	44.49%	45.29%	10.22%	6586	Tethered binding most frequent
SRF (MADS box)	HCT116	SRF_representative_N1	FOS (bZIP)	34.90%	54.87%	10.24%	4943	Tethered binding most frequent
STAT3 (STAT)	MCF10A	STAT3_representative_N1	FOSL1 (bZIP)	28.29%	59.76%	11.95%	44836	Tethered binding most frequent
STAT3 (STAT)	MCF10A	STAT3_representative_N1	SP4 (C2H2 ZF)	49.33%	39.33%	11.34%	29734	Tethered binding less frequent than canonical binding but more frequent than co-binding
STAT3 (STAT)	MCF10A	STAT3_representative_N1	FOS (bZIP)	40.12%	46.85%	13.02%	15021	Tethered binding most frequent
STAT3 (STAT)	MCF10A	STAT3_representative_N1	KLF5 (C2H2 ZF)	42.13%	44.01%	13.85%	14259	Tethered binding most frequent
STAT3 (STAT)	MCF10A	STAT3_representative_N1	FOS (bZIP)	31.32%	57.26%	11.42%	44583	Tethered binding most frequent
STAT3 (STAT)	MCF10A	STAT3_representative_N1	CEBPE (bZIP)	57.51%	35.29%	7.20%	29444	Tethered binding less frequent than canonical binding but more frequent than co-binding
STAT3 (STAT)	HeLa-S3	STAT3_representative_N1	FOS (bZIP)	23.34%	66.07%	10.59%	10122	Tethered binding most frequent
STAT5A (STAT)	GM12878	STAT5A_K562_ENCSR000BRR_merged_N2	JUND (bZIP)	43.54%	52.93%	3.52%	8543	Tethered binding most frequent
STAT5A (STAT)	K562	STAT5A_K562_ENCSR000BRR_merged_N2	GATA2 (GATA)	29.24%	61.39%	9.37%	5452	Tethered binding most frequent
STAT5B (STAT)	HepG2	MA1625.1 from JASPAR	REST (C2H2 ZF)	20.80%	74.76%	4.43%	1466	Tethered binding most frequent

STAT5B (STAT)	HepG2	MA1625.1 from JASPAR	ZNF93 (C2H2 ZF)	3.62%	91.93%	4.45%	4583	Tethered binding most frequent
STAT6 (STAT)	HepG2	MA0520.1 from JASPAR	FOXC2 (Forkhead)	14.56%	79.95%	5.49%	364	Tethered binding most frequent
TAL1 (bHLH)	K562	TAL1_rv3	GATA2 (GATA)	28.90%	61.62%	9.49%	13347	Tethered binding most frequent
TAL1 (bHLH)	K562	TAL1_rv3	GATA5 (GATA)	26.99%	63.87%	9.13%	11429	Tethered binding most frequent
TAL1 (bHLH)	K562	TAL1_rv3	ZNF384 (C2H2 ZF)	18.96%	70.34%	10.70%	13920	Tethered binding most frequent
TBX2 (T-box)	HepG2	M03556_2.00 from Cis-BP	FOXC2 (Forkhead)	19.56%	76.44%	4.00%	3875	Tethered binding most frequent
TBX21 (T-box)	GM12878	TBX21_GM12878_ENCSR739IHN_merged_N1	RUNX1 (Runt)	51.54%	39.90%	8.56%	20927	Tethered binding less frequent than canonical binding but more frequent than co-binding
TBX3 (T-box)	HepG2	TBX3_HepG2_ENCSR238QRG_merged_N2	FOXC2 (Forkhead)	29.72%	62.87%	7.41%	1134	Tethered binding most frequent
TCF12 (bHLH)	HepG2	TCF12_representative_N1	FOXC2 (Forkhead)	34.65%	57.17%	8.17%	2043	Tethered binding most frequent
TCF12 (bHLH)	HepG2	TCF12_representative_N1	HNF1A (Homeodomain)	41.49%	50.20%	8.31%	1757	Tethered binding most frequent
TCF12 (bHLH)	HepG2	TCF12_representative_N1	PRDM9 (C2H2 ZF)	12.25%	73.59%	14.16%	3313	Tethered binding most frequent
TCF12 (bHLH)	A549	TCF12_representative_N1	FOXC2 (Forkhead)	43.31%	50.93%	5.76%	15903	Tethered binding most frequent
TCF12 (bHLH)	A549	TCF12_representative_N1	BACH1 (bZIP)	39.02%	52.31%	8.67%	16364	Tethered binding most frequent
TCF12 (bHLH)	MCF-7	TCF12_representative_N1	FOXC2 (Forkhead)	26.38%	65.76%	7.86%	4681	Tethered binding most frequent
TCF12 (bHLH)	MCF-7	TCF12_representative_N1	ESR2 (Nuclear receptor	40.99%	49.87%	9.13%	3198	Tethered binding most frequent
TCF12 (bHLH)	Ishikawa	TCF12_representative_N1	ZIC3 (C2H2 ZF)	20.74%	49.71%	29.55%	14347	Tethered binding most frequent

TCF12 (bHLH)	Ishikawa	TCF12_representative_N1	TEAD3 (TEA)	57.43%	35.72%	6.85%	11224	Tethered binding less frequent than canonical binding but more frequent than co-binding
TCF12 (bHLH)	K562	TCF12_representative_N1	GATA2 (GATA)	22.59%	65.26%	12.15%	5498	Tethered binding most frequent
TCF12 (bHLH)	K562	TCF12_representative_N1	GATA3 (GATA)	31.36%	56.79%	11.86%	4420	Tethered binding most frequent
TCF12 (bHLH)	HepG2	TCF12_representative_N1	FOXC2 (Forkhead)	37.35%	51.37%	11.28%	656	Tethered binding most frequent
TCF12 (bHLH)	HepG2	TCF12_representative_N1	FOXC2 (Forkhead)	38.36%	51.45%	10.20%	657	Tethered binding most frequent
TCF12 (bHLH)	K562	TCF12_representative_N1	GATA5 (GATA)	29.90%	59.63%	10.47%	11017	Tethered binding most frequent
TCF12 (bHLH)	K562	TCF12_representative_N1	ZNF384 (C2H2 ZF)	22.45%	66.46%	11.09%	13261	Tethered binding most frequent
TCF3 (bHLH)	GM12878	TCF3_rv3	IRF1 (IRF)	54.33%	34.61%	11.06%	22306	Tethered binding less frequent than canonical binding but more frequent than co-binding
TCF3 (bHLH)	K562	TCF3_rv3	GATA2 (GATA)	20.03%	69.57%	10.40%	14067	Tethered binding most frequent
TCF3 (bHLH)	K562	TCF3_rv3	GATA2 (GATA)	30.25%	60.88%	8.86%	10944	Tethered binding most frequent
TCF7 (HMG/Sox)	HepG2	TCF7_representative_N1	FOXC2 (Forkhead)	46.72%	39.34%	13.94%	10114	Tethered binding less frequent than canonical binding but more frequent than co-binding
TCF7 (HMG/Sox)	K562	TCF7_representative_N1	GATA2 (GATA)	48.01%	37.40%	14.59%	1460	Tethered binding less frequent than canonical binding but more frequent than co-binding

TCF7L2 (HMG/Sox)	HCT116	TCF7L2_representative_N1	ELF3 (Ets; AT hook)	64.86%	26.70%	8.43%	6082	Tethered binding less frequent than canonical binding but more frequent than co-binding
TCF7L2 (HMG/Sox)	HepG2	TCF7L2_representative_N1	FOXC1 (Forkhead)	50.91%	30.72%	18.37%	2194	Tethered binding less frequent than canonical binding but more frequent than co-binding
TCF7L2 (HMG/Sox)	HepG2	TCF7L2_representative_N1	FOXC2 (Forkhead)	41.92%	44.49%	13.60%	7861	Tethered binding most frequent
TEAD4 (TEA)	K562	TEAD4_rv3	GATA2 (GATA)	39.99%	53.90%	6.11%	24168	Tethered binding most frequent
TEAD4 (TEA)	K562	TEAD4_rv3	FERD3L (bHLH)	46.89%	47.82%	5.30%	21350	Tethered binding most frequent
TEAD4 (TEA)	HepG2	TEAD4_rv3	FOXC1 (Forkhead)	50.62%	35.40%	13.98%	10686	Tethered binding less frequent than canonical binding but more frequent than co-binding
TEAD4 (TEA)	A549	TEAD4_rv3	REST (C2H2 ZF)	52.93%	41.46%	5.60%	8564	Tethered binding less frequent than canonical binding but more frequent than co-binding
TEAD4 (TEA)	SK-N-SH	TEAD4_rv3	ATF3 (bZIP)	32.74%	54.52%	12.74%	16982	Tethered binding most frequent
TEAD4 (TEA)	HCT116	TEAD4_rv3	FOS (bZIP)	41.51%	39.26%	19.23%	5533	Tethered binding less frequent than canonical binding but more frequent than co-binding
TFAP4 (bHLH)	HepG2	TFAP4_representativeHSAv2_N1	FOXC2 (Forkhead)	72.43%	17.22%	10.35%	19995	Tethered binding less frequent than canonical binding but more frequent than co-binding

TFAP4 (bHLH)	HepG2	TFAP4_representativeHSAv2_N1	FOXC2 (Forkhead)	66.17%	24.89%	8.94%	28638	Tethered binding less frequent than canonical binding but more frequent than co-binding
TFAP4 (bHLH)	HepG2	TFAP4_representativeHSAv2_N1	HNF4A (Nuclear recept	65.77%	24.38%	9.86%	28442	Tethered binding less frequent than canonical binding but more frequent than co-binding
TFAP4 (bHLH)	K562	TFAP4_representativeHSAv2_N1	PATZ1 (C2H2 ZF; AT h	24.73%	49.57%	25.71%	14024	Tethered binding most frequent
TFDP1 (E2F)	HepG2	TFDP1_representativeHSAv2_N1	NFYA (CBF/NF-Y)	71.93%	19.94%	8.12%	12727	Tethered binding less frequent than canonical binding but more frequent than co-binding
TFDP2 (E2F)	HepG2	TFDP2_HepG2_ENCSR069JKP_merged_N1	SP2 (C2H2 ZF)	18.20%	52.16%	29.64%	16634	Tethered binding most frequent
TFDP2 (E2F)	HepG2	TFDP2_HepG2_ENCSR069JKP_merged_N1	NFYA (CBF/NF-Y)	69.98%	19.86%	10.16%	9930	Tethered binding less frequent than canonical binding but more frequent than co-binding
TGIF2 (Homeodomain)	WTC11	TGIF2_representativeHSAv2_N1	REST (C2H2 ZF)	24.20%	70.48%	5.32%	1504	Tethered binding most frequent
TGIF2 (Homeodomain)	HepG2	TGIF2_representativeHSAv2_N1	REST (C2H2 ZF)	35.50%	59.08%	5.42%	4763	Tethered binding most frequent
THAP1 (THAP finger)	K562	MA0597.2 from JASPAR	YY1 (C2H2 ZF)	80.26%	13.51%	6.23%	11324	Tethered binding less frequent than canonical binding but more frequent than co-binding

THAP1 (THAP finger)	K562	MA0597.2 from JASPAR	ELK1 (Ets)	59.43%	27.80%	12.77%	13565	Tethered binding less frequent than canonical binding but more frequent than co-binding
THAP12 (THAP finger)	K562	M02538_2.00 from Cis-BP	ZNF574 (C2H2 ZF)	20.72%	77.05%	2.24%	671	Tethered binding most frequent
THAP9 (THAP finger)	HepG2	THAP9_HepG2_ENCSR123GPC_merged_N3	ELK4 (Ets)	57.20%	37.52%	5.28%	13264	Tethered binding less frequent than canonical binding but more frequent than co-binding
THAP9 (THAP finger)	HepG2	THAP9_HepG2_ENCSR123GPC_merged_N3	YY1 (C2H2 ZF)	63.22%	32.03%	4.76%	12193	Tethered binding less frequent than canonical binding but more frequent than co-binding
THRA (Nuclear receptor)	HepG2	THRA_representativeHSAv2_N1	FOXC2 (Forkhead)	52.64%	41.29%	6.08%	16131	Tethered binding less frequent than canonical binding but more frequent than co-binding
THRB (Nuclear receptor)	HepG2	THRB_representativeHSAv2_N1	FOXC2 (Forkhead)	70.34%	21.01%	8.65%	7687	Tethered binding less frequent than canonical binding but more frequent than co-binding
USF1 (bHLH)	GM12878	USF1_representativeHSAv2_N1	SP2 (C2H2 ZF)	58.61%	11.45%	29.94%	8174	Co-binding less frequent than canonical binding but more frequent than tethered binding

USF1 (bHLH)	HepG2	USF1_representativeHSAv2_N1	SP4 (C2H2 ZF)	60.20%	19.32%	20.48%	30266	Co-binding less frequent than canonical binding but more frequent than tethered binding
USF1 (bHLH)	A549	USF1_representativeHSAv2_N1	KLF12 (C2H2 ZF)	50.41%	19.76%	29.83%	6892	Co-binding less frequent than canonical binding but more frequent than tethered binding
USF1 (bHLH)	K562	USF1_representativeHSAv2_N1	NFYB (Unknown)	78.85%	7.04%	14.12%	31427	Co-binding less frequent than canonical binding but more frequent than tethered binding
USF1 (bHLH)	SK-N-SH	USF1_representativeHSAv2_N1	SP4 (C2H2 ZF)	55.20%	29.78%	15.02%	40563	Tethered binding less frequent than canonical binding but more frequent than co-binding
USF1 (bHLH)	Ishikawa	USF1_representativeHSAv2_N1	SP4 (C2H2 ZF)	47.96%	31.28%	20.76%	27290	Tethered binding less frequent than canonical binding but more frequent than co-binding
USF1 (bHLH)	SK-N-SH	USF1_representativeHSAv2_N1	SP2 (C2H2 ZF)	60.30%	14.60%	25.10%	10244	Co-binding less frequent than canonical binding but more frequent than tethered binding

USF1 (bHLH)	HCT116	USF1_representativeHSAv2_N1	NFYB (Unknown)	71.27%	4.76%	23.97%	9356	Co-binding less frequent than canonical binding but more frequent than tethered binding
USF1 (bHLH)	HCT116	USF1_representativeHSAv2_N1	THAP11 (THAP finger)	77.35%	4.09%	18.56%	9291	Co-binding less frequent than canonical binding but more frequent than tethered binding
USF1 (bHLH)	HepG2	USF1_representativeHSAv2_N1	KLF12 (C2H2 ZF)	39.99%	42.50%	17.51%	40911	Tethered binding most frequent
USF1 (bHLH)	WTC11	USF1_representativeHSAv2_N1	SP2 (C2H2 ZF)	36.85%	23.74%	39.41%	5720	Co-binding most frequent
USF1 (bHLH)	WTC11	USF1_representativeHSAv2_N1	NFYB (Unknown)	72.76%	7.47%	19.77%	4714	Co-binding less frequent than canonical binding but more frequent than tethered binding
USF1 (bHLH)	K562	USF1_representativeHSAv2_N1	NFYA (CBF/NF-Y)	72.90%	5.72%	21.38%	7487	Co-binding less frequent than canonical binding but more frequent than tethered binding
USF2 (bHLH)	GM12878	USF2_representativeHSAv2_N1	SP4 (C2H2 ZF)	50.80%	11.23%	37.97%	3571	Co-binding less frequent than canonical binding but more frequent than tethered binding

USF2 (bHLH)	H1	USF2_representativeHSAv2_N1	NFYB (Unknown)	72.47%	6.92%	20.61%	6270	Co-binding less frequent than canonical binding but more frequent than tethered binding
USF2 (bHLH)	H1	USF2_representativeHSAv2_N1	SP4 (C2H2 ZF)	49.62%	15.55%	34.83%	6911	Co-binding less frequent than canonical binding but more frequent than tethered binding
USF2 (bHLH)	HepG2	USF2_representativeHSAv2_N1	KLF12 (C2H2 ZF)	53.25%	7.66%	39.09%	2090	Co-binding less frequent than canonical binding but more frequent than tethered binding
USF2 (bHLH)	IMR-90	USF2_representativeHSAv2_N1	NFYB (Unknown)	76.20%	12.61%	11.19%	19385	Tethered binding less frequent than canonical binding but more frequent than co-binding
USF2 (bHLH)	IMR-90	USF2_representativeHSAv2_N1	KLF12 (C2H2 ZF)	49.93%	33.86%	16.21%	25612	Tethered binding less frequent than canonical binding but more frequent than co-binding
USF2 (bHLH)	A549	USF2_representativeHSAv2_N1	NFYA (CBF/NF-Y)	74.63%	6.33%	19.04%	6906	Co-binding less frequent than canonical binding but more frequent than tethered binding
USF2 (bHLH)	SK-N-SH	USF2_representativeHSAv2_N1	SP4 (C2H2 ZF)	33.37%	11.16%	55.47%	1909	Co-binding most frequent

WT1 (C2H2 ZF)	HEK293	WT1_HEK293_ENCSR966PJJ_merged_N1	FOXE1 (Forkhead)	64.85%	26.71%	8.44%	16628	Tethered binding less frequent than canonical binding but more frequent than co-binding
XBP1 (bZIP)	HepG2	M04010_2.00 from Cis-BP	REST (C2H2 ZF)	29.69%	63.32%	6.99%	229	Tethered binding most frequent
YY1 (C2H2 ZF)	SK-N-SH	YY1_representative_N1	ETV6 (Ets)	68.81%	21.55%	9.64%	9918	Tethered binding less frequent than canonical binding but more frequent than co-binding
ZBTB10 (C2H2 ZF)	HepG2	ZBTB10_representativeHSAv2_N1	FOXC2 (Forkhead)	80.36%	10.12%	9.51%	494	Tethered binding less frequent than canonical binding but more frequent than co-binding
ZBTB11 (C2H2 ZF)	GM12878	ZBTB11_representativeHSAv2_N2	ELK4 (Ets)	11.94%	60.86%	27.20%	603	Tethered binding most frequent
ZBTB11 (C2H2 ZF)	MCF-7	ZBTB11_representativeHSAv2_N2	ELK4 (Ets)	8.99%	62.04%	28.97%	1001	Tethered binding most frequent
ZBTB11 (C2H2 ZF)	K562	ZBTB11_representativeHSAv2_N2	ELK4 (Ets)	11.13%	68.12%	20.75%	2516	Tethered binding most frequent
ZBTB11 (C2H2 ZF)	K562	ZBTB11_representativeHSAv2_N2	ELK4 (Ets)	18.06%	64.03%	17.90%	620	Tethered binding most frequent
ZBTB11 (C2H2 ZF)	K562	ZBTB11_representativeHSAv2_N2	ELF1 (Ets)	13.07%	74.59%	12.34%	5105	Tethered binding most frequent
ZBTB11 (C2H2 ZF)	HEK293	ZBTB11_representativeHSAv2_N2	ELK4 (Ets)	16.98%	66.65%	16.37%	4099	Tethered binding most frequent
ZBTB11 (C2H2 ZF)	K562	ZBTB11_representativeHSAv2_N2	ELK4 (Ets)	8.35%	68.94%	22.71%	982	Tethered binding most frequent
ZBTB12 (C2H2 ZF)	K562	M04634_2.00 from Cis-BP	JDP2 (bZIP)	21.82%	62.81%	15.37%	1366	Tethered binding most frequent

ZBTB2 (C2H2 ZF)	HepG2	ZBTB2_representativeHSAv2_N2	ELK4 (Ets)	63.62%	9.15%	27.23%	3463	Co-binding less frequent than canonical binding but more frequent than tethered binding
ZBTB2 (C2H2 ZF)	K562	ZBTB2_representativeHSAv2_N2	NRF1 (Unknown)	78.34%	9.66%	12.01%	19647	Co-binding less frequent than canonical binding but more frequent than tethered binding
ZBTB2 (C2H2 ZF)	K562	ZBTB2_representativeHSAv2_N2	NRF1 (Unknown)	77.94%	10.77%	11.30%	19892	Co-binding less frequent than canonical binding but more frequent than tethered binding
ZBTB21 (C2H2 ZF)	HEK293	ZBTB21_representativeHSAv2_N3	CREB3 (bZIP)	52.52%	34.78%	12.70%	12877	Tethered binding less frequent than canonical binding but more frequent than co-binding
ZBTB21 (C2H2 ZF)	HEK293	ZBTB21_representativeHSAv2_N3	JUN (bZIP)	55.18%	31.79%	13.04%	12313	Tethered binding less frequent than canonical binding but more frequent than co-binding
ZBTB21 (C2H2 ZF)	HepG2	ZBTB21_representativeHSAv2_N3	ATF3 (bZIP)	70.87%	18.28%	10.85%	12933	Tethered binding less frequent than canonical binding but more frequent than co-binding

ZBTB21 (C2H2 ZF)	HepG2	ZBTB21_representativeHSAv2_N3	ELK4 (Ets)	68.62%	12.55%	18.82%	12086	Co-binding less frequent than canonical binding but more frequent than tethered binding
ZBTB26 (C2H2 ZF)	HEK293	ZBTB26_representativeHSAv2_N1	E2F2 (E2F)	28.24%	17.92%	53.84%	10829	Co-binding most frequent
ZBTB33 (C2H2 ZF)	HepG2	ZBTB33_representative_N1	PATZ1 (C2H2 ZF; AT h	17.30%	33.24%	49.46%	1775	Co-binding most frequent
ZBTB34 (C2H2 ZF)	HepG2	ZBTB34_K562_ENCSR567QAD_merged_N1	FOXC2 (Forkhead)	8.21%	88.42%	3.37%	475	Tethered binding most frequent
ZBTB34 (C2H2 ZF)	HepG2	ZBTB34_K562_ENCSR567QAD_merged_N1	HNF4G (Nuclear recept	9.09%	87.80%	3.10%	451	Tethered binding most frequent
ZBTB37 (C2H2 ZF)	HepG2	M04603_2.00 from Cis-BP	FOXC2 (Forkhead)	12.59%	84.93%	2.48%	564	Tethered binding most frequent
ZBTB40 (C2H2 ZF)	GM12878	ZBTB40_representativeHSAv2_N2	ELK4 (Ets)	62.49%	35.81%	1.70%	9046	Tethered binding less frequent than canonical binding but more frequent than co-binding
ZBTB40 (C2H2 ZF)	MCF-7	ZBTB40_representativeHSAv2_N2	ELF1 (Ets)	72.64%	26.72%	0.64%	9704	Tethered binding less frequent than canonical binding but more frequent than co-binding
ZBTB40 (C2H2 ZF)	MCF-7	ZBTB40_representativeHSAv2_N2	ELK4 (Ets)	67.53%	30.60%	1.86%	10247	Tethered binding less frequent than canonical binding but more frequent than co-binding
ZBTB40 (C2H2 ZF)	HepG2	ZBTB40_representativeHSAv2_N2	ELK4 (Ets)	55.77%	42.53%	1.70%	2885	Tethered binding less frequent than canonical binding but more frequent than co-binding

ZBTB42 (C2H2 ZF)	HepG2	ZBTB42_HepG2_ENCSR232AAR_merged_N1	FOXC2 (Forkhead)	78.47%	7.77%	13.77%		1017	Co-binding less frequent than canonical binding but more frequent than tethered binding
ZBTB43 (C2H2 ZF)	HepG2	ZBTB43_HepG2_ENCSR173USN_merged_N2	FOXD2 (Forkhead)	41.03%	43.78%	15.19%		619	Tethered binding most frequent
ZBTB7B (C2H2 ZF)	HepG2	ZBTB7B_representativeHSAv2_N1	FOXC2 (Forkhead)	67.72%	25.85%	6.44%		20286	Tethered binding less frequent than canonical binding but more frequent than co-binding
ZEB1 (C2H2 ZF; Homeodomain)	GM12878	ZEB1_representative_N1	SCRT2 (C2H2 ZF)	100.00%	0.00%	0.00%		19171	not co-binding
ZEB2 (C2H2 ZF; Homeodomain)	K562	ZEB2_rv3	SCRT2 (C2H2 ZF)	100.00%	0.00%	0.00%		9745	not co-binding
ZEB2 (C2H2 ZF; Homeodomain)	K562	ZEB2_rv3	GATA2 (GATA)	53.10%	40.52%	6.38%		16384	Tethered binding less frequent than canonical binding but more frequent than co-binding
ZEB2 (C2H2 ZF; Homeodomain)	K562	ZEB2_rv3	THAP11 (THAP finger)	67.32%	26.89%	5.78%		13330	Tethered binding less frequent than canonical binding but more frequent than co-binding
ZEB2 (C2H2 ZF; Homeodomain)	K562	ZEB2_rv3	GATA3 (GATA)	57.03%	37.73%	5.25%		12754	Tethered binding less frequent than canonical binding but more frequent than co-binding
ZFHX2 (Homeodomain)	HEK293	ZFHX2_HEK293_ENCSR632SIM_merged_N3	EGR1 (C2H2 ZF)	7.74%	31.47%	60.79%		41401	Co-binding most frequent

ZFHX2 (Homeodomain)	HEK293	ZFHX2_HEK293_ENCSR632SIM_merged_N3	EGR1 (C2H2 ZF)	15.73%	28.86%	55.41%	39885	Co-binding most frequent
ZFHX3 (C2H2 ZF; Homeodomain)	HepG2	M01849_2.00 from Cis-BP	TCF7L2 (HMG/Sox)	24.80%	67.15%	8.05%	621	Tethered binding most frequent
ZFHX3 (C2H2 ZF; Homeodomain)	HepG2	M01849_2.00 from Cis-BP	NR2F6 (Nuclear recepto	21.71%	70.86%	7.43%	700	Tethered binding most frequent
ZFHX3 (C2H2 ZF; Homeodomain)	HepG2	M01849_2.00 from Cis-BP	FOXC2 (Forkhead)	13.16%	79.35%	7.49%	988	Tethered binding most frequent
ZFHX3 (C2H2 ZF; Homeodomain)	HepG2	M01849_2.00 from Cis-BP	ZNF281 (C2H2 ZF)	26.32%	65.13%	8.55%	585	Tethered binding most frequent
ZFP41 (C2H2 ZF)	HepG2	M04598_2.00 from Cis-BP	MYC (bHLH)	24.95%	71.46%	3.59%	918	Tethered binding most frequent
ZFP91 (C2H2 ZF)	K562	ZFP91_rv3	PATZ1 (C2H2 ZF; AT h	42.25%	6.22%	51.53%	1013	Co-binding most frequent
ZHX1 (Homeodomain)	HepG2	M02093_2.00 from Cis-BP	CREM (bZIP)	4.46%	95.12%	0.42%	471	Tethered binding most frequent
ZHX1 (Homeodomain)	HepG2	M02093_2.00 from Cis-BP	YY1 (C2H2 ZF)	2.77%	95.75%	1.48%	541	Tethered binding most frequent
ZHX2 (Homeodomain)	HepG2	ZHX2_HepG2_ENCSR401VBM_merged_N1	REST (C2H2 ZF)	73.10%	9.82%	17.07%	1517	Co-binding less frequent than canonical binding but more frequent than tethered binding
ZHX2 (Homeodomain)	HepG2	ZHX2_HepG2_ENCSR401VBM_merged_N1	FOXC2 (Forkhead)	83.30%	14.45%	2.25%	1599	Tethered binding less frequent than canonical binding but more frequent than co-binding
ZKSCAN8 (C2H2 ZF)	HepG2	ZKSCAN8_rv3	FOXC2 (Forkhead)	30.04%	63.80%	6.16%	12312	Tethered binding most frequent
ZNF10 (C2H2 ZF)	HEK293	ZNF10_HEK293_ENCSR019WUS_merged_N2	DUXA (Homeodomain)	66.12%	15.63%	18.24%	10196	Co-binding less frequent than canonical binding but more frequent than tethered binding

ZNF143 (C2H2 ZF)	GM12878	ZNF143_representativeHSAv2_N3	THAP11 (THAP finger)	68.39%	13.98%	17.63%	18331	Co-binding less frequent than canonical binding but more frequent than tethered binding
ZNF143 (C2H2 ZF)	H1-hESC	ZNF143_representativeHSAv2_N3	THAP11 (THAP finger)	65.93%	16.36%	17.72%	19124	Co-binding less frequent than canonical binding but more frequent than tethered binding
ZNF143 (C2H2 ZF)	H1-hESC	ZNF143_representativeHSAv2_N3	THAP11 (THAP finger)	61.52%	19.44%	19.05%	19855	Tethered binding less frequent than canonical binding but more frequent than co-binding
ZNF143 (C2H2 ZF)	HeLa-S3	ZNF143_representativeHSAv2_N3	THAP11 (THAP finger)	52.69%	9.53%	37.79%	5060	Co-binding less frequent than canonical binding but more frequent than tethered binding
ZNF143 (C2H2 ZF)	HeLa-S3	ZNF143_representativeHSAv2_N3	THAP11 (THAP finger)	54.09%	9.09%	36.81%	5036	Co-binding less frequent than canonical binding but more frequent than tethered binding
ZNF143 (C2H2 ZF)	K562	ZNF143_representativeHSAv2_N3	THAP11 (THAP finger)	45.80%	22.03%	32.16%	13689	Co-binding less frequent than canonical binding but more frequent than tethered binding

ZNF143 (C2H2 ZF)	K562	ZNF143_representativeHSAv2_N3	THAP11 (THAP finger)	52.48%	19.66%	27.87%	13284	Co-binding less frequent than canonical binding but more frequent than tethered binding
ZNF143 (C2H2 ZF)	WTC11	ZNF143_representativeHSAv2_N3	THAP11 (THAP finger)	30.11%	9.39%	60.51%	3036	Co-binding most frequent
ZNF143 (C2H2 ZF)	WTC11	ZNF143_representativeHSAv2_N3	THAP11 (THAP finger)	27.95%	9.66%	62.40%	3045	Co-binding most frequent
ZNF143 (C2H2 ZF)	WTC11	ZNF143_representativeHSAv2_N3	THAP11 (THAP finger)	29.10%	9.45%	61.45%	3038	Co-binding most frequent
ZNF143 (C2H2 ZF)	WTC11	ZNF143_representativeHSAv2_N3	THAP11 (THAP finger)	29.97%	9.39%	60.64%	3036	Co-binding most frequent
ZNF143 (C2H2 ZF)	HepG2	ZNF143_representativeHSAv2_N3	THAP11 (THAP finger)	49.11%	7.92%	42.97%	4480	Co-binding less frequent than canonical binding but more frequent than tethered binding
ZNF143 (C2H2 ZF)	HepG2	ZNF143_representativeHSAv2_N3	THAP11 (THAP finger)	49.19%	7.94%	42.87%	4481	Co-binding less frequent than canonical binding but more frequent than tethered binding
ZNF143 (C2H2 ZF)	K562	ZNF143_representativeHSAv2_N3	THAP11 (THAP finger)	30.70%	6.90%	62.40%	2479	Co-binding most frequent
ZNF143 (C2H2 ZF)	K562	ZNF143_representativeHSAv2_N3	THAP11 (THAP finger)	30.56%	6.94%	62.50%	2480	Co-binding most frequent
ZNF143 (C2H2 ZF)	K562	ZNF143_representativeHSAv2_N3	THAP11 (THAP finger)	28.16%	7.16%	64.68%	2486	Co-binding most frequent
ZNF143 (C2H2 ZF)	K562	ZNF143_representativeHSAv2_N3	THAP11 (THAP finger)	28.49%	7.12%	64.39%	2485	Co-binding most frequent

ZNF143 (C2H2 ZF)	GM12878	ZNF143_representativeHSAv2_N3	THAP11 (THAP finger)	67.27%	16.09%	16.63%	17056	Co-binding less frequent than canonical binding but more frequent than tethered binding
ZNF143 (C2H2 ZF)	GM12878	ZNF143_representativeHSAv2_N3	THAP11 (THAP finger)	38.33%	27.58%	34.09%	19762	Co-binding less frequent than canonical binding but more frequent than tethered binding
ZNF18 (C2H2 ZF)	HepG2	ZNF18_HEK293_ENCSR977HTH_merged_N2	TBX6 (T-box)	10.41%	49.49%	40.10%	788	Tethered binding most frequent
ZNF18 (C2H2 ZF)	HepG2	ZNF18_HEK293_ENCSR977HTH_merged_N2	EOMES (T-box)	10.69%	49.94%	39.37%	795	Tethered binding most frequent
ZNF18 (C2H2 ZF)	HEK293	ZNF18_HEK293_ENCSR977HTH_merged_N2	TBX6 (T-box)	24.39%	35.59%	40.02%	21023	Co-binding most frequent
ZNF217 (C2H2 ZF)	MCF-7	ZNF217_HepG2_ENCSR055FQB_merged_N3	GRHL1 (Grainyhead)	31.62%	52.79%	15.59%	3656	Tethered binding most frequent
ZNF217 (C2H2 ZF)	HepG2	ZNF217_HepG2_ENCSR055FQB_merged_N3	FOXC2 (Forkhead)	67.58%	22.13%	10.28%	17648	Tethered binding less frequent than canonical binding but more frequent than co-binding
ZNF217 (C2H2 ZF)	HepG2	ZNF217_HepG2_ENCSR055FQB_merged_N3	HNF4G (Nuclear recept	49.13%	31.49%	19.38%	20057	Tethered binding less frequent than canonical binding but more frequent than co-binding

ZNF217 (C2H2 ZF)	MCF-7	ZNF217_HepG2_ENCSR055FQB_merged_N3	THAP11 (THAP finger)	73.80%	16.32%	9.88%	17411	Tethered binding less frequent than canonical binding but more frequent than co-binding
ZNF24 (C2H2 ZF)	K562	ZNF24_rv3	ZNF524 (C2H2 ZF; AT l	75.61%	21.90%	2.49%	20649	Tethered binding less frequent than canonical binding but more frequent than co-binding
ZNF300 (C2H2 ZF)	HEK293	M07594_2.00 from Cis-BP	NFIA (SMAD)	41.61%	52.20%	6.19%	1067	Tethered binding most frequent
ZNF316 (C2H2 ZF)	K562	ZNF316_representative_N1	MAFG (bZIP)	13.69%	80.97%	5.34%	55335	Tethered binding most frequent
ZNF354B (C2H2 ZF)	K562	ZNF354B_K562_ENCSR674SCQ_merged_N2	USF1 (bHLH)	48.73%	47.27%	4.00%	1375	Tethered binding less frequent than canonical binding but more frequent than co-binding
ZNF423 (C2H2 ZF)	HEK293	ZNF423_rv3	EBF3 (EBF1)	33.00%	17.24%	49.76%	8239	Co-binding most frequent
ZNF444 (C2H2 ZF)	MCF-7	ZNF444_rv3	ELF1 (Ets)	87.30%	4.57%	8.13%	20307	Co-binding less frequent than canonical binding but more frequent than tethered binding
ZNF503 (C2H2 ZF)	HepG2	ZNF503_HepG2_ENCSR998YJI_merged_N3	FOXC2 (Forkhead)	16.71%	59.86%	23.43%	700	Tethered binding most frequent
ZNF503 (C2H2 ZF)	HepG2	ZNF503_HepG2_ENCSR998YJI_merged_N3	HNF1A (Homeodomain)	38.94%	45.01%	16.05%	511	Tethered binding most frequent
ZNF518A (C2H2 ZF)	HEK293	ZNF518A_HEK293_ENCSR159GFL_merged_N2	ELK4 (Ets)	68.54%	29.29%	2.17%	12478	Tethered binding less frequent than canonical binding but more frequent than co-binding

ZNF552 (C2H2 ZF)	HepG2	M07655_2.00 from Cis-BP	MYC (bHLH)	24.80%	51.97%	23.23%	508	Tethered binding most frequent
ZNF562 (C2H2 ZF)	HepG2	ZNF562_HepG2_ENCSR727IJD_merged_N1	SOX11 (HMG/Sox)	13.11%	3.83%	83.06%	183	Co-binding most frequent
ZNF574 (C2H2 ZF)	HepG2	ZNF574_representativeHSAv2_N2	ELK4 (Ets)	24.02%	70.10%	5.88%	5733	Tethered binding most frequent
ZNF580 (C2H2 ZF)	HEK293	ZNF580_HepG2_ENCSR173CTF_merged_N1	FEV (Ets)	49.02%	47.22%	3.76%	5078	Tethered binding less frequent than canonical binding but more frequent than co-binding
ZNF619 (C2H2 ZF)	HepG2	M07649_2.00 from Cis-BP	ELK4 (Ets)	71.88%	18.31%	9.81%	10174	Tethered binding less frequent than canonical binding but more frequent than co-binding
ZNF629 (C2H2 ZF)	HEK293	ZNF629_rv3	CREB5 (bZIP)	25.74%	68.87%	5.39%	13519	Tethered binding most frequent
ZNF629 (C2H2 ZF)	HepG2	ZNF629_rv3	ESRRA (Nuclear recept	61.34%	2.43%	36.23%	9536	Co-binding less frequent than canonical binding but more frequent than tethered binding
ZNF639 (C2H2 ZF)	HEK293	ZNF639_K562_ENCSR497VFH_merged_N1	ATF3 (bZIP)	71.28%	20.18%	8.54%	19494	Tethered binding less frequent than canonical binding but more frequent than co-binding
ZNF639 (C2H2 ZF)	HepG2	ZNF639_K562_ENCSR497VFH_merged_N1	ELK4 (Ets)	77.00%	8.20%	14.80%	24441	Co-binding less frequent than canonical binding but more frequent than tethered binding

ZNF639 (C2H2 ZF)	K562	ZNF639_K562_ENCSR497VFH_merged_N1	NRF1 (Unknown)	64.83%	12.26%	22.91%	15681	Co-binding less frequent than canonical binding but more frequent than tethered binding
ZNF639 (C2H2 ZF)	K562	ZNF639_K562_ENCSR497VFH_merged_N1	NRF1 (Unknown)	64.24%	12.43%	23.34%	15710	Co-binding less frequent than canonical binding but more frequent than tethered binding
ZNF644 (C2H2 ZF)	HepG2	ZNF644_HepG2_ENCSR578CXC_merged_N2	HNF4G (Nuclear recept	32.10%	60.72%	7.18%	26375	Tethered binding most frequent
ZNF644 (C2H2 ZF)	K562	ZNF644_HepG2_ENCSR578CXC_merged_N2	HNF4G (Nuclear recept	31.48%	61.13%	7.38%	24839	Tethered binding most frequent
ZNF644 (C2H2 ZF)	K562	ZNF644_HepG2_ENCSR578CXC_merged_N2	FOXC2 (Forkhead)	41.21%	52.52%	6.27%	20333	Tethered binding most frequent
ZNF644 (C2H2 ZF)	K562	ZNF644_HepG2_ENCSR578CXC_merged_N2	CEBPD (bZIP)	55.12%	39.70%	5.17%	16011	Tethered binding less frequent than canonical binding but more frequent than co-binding
ZNF746 (C2H2 ZF)	HepG2	ZNF746_HepG2_ENCSR591MYB_merged_N1	FOXC2 (Forkhead)	83.16%	7.86%	8.97%	3065	Co-binding less frequent than canonical binding but more frequent than tethered binding
ZNF76 (C2H2 ZF)	HEK293	ZNF76_representativeHSAv2_N3	THAP11 (THAP finger)	57.14%	20.16%	22.70%	7704	Co-binding less frequent than canonical binding but more frequent than tethered binding

ZSCAN25 (C2H2 ZF)	HepG2	ZSCAN25_HepG2_ENCSR037LQB_merged_N1	HNF4G (Nuclear recept	66.91%	15.77%	17.32%	10671	Co-binding less frequent than canonical binding but more frequent than tethered binding
ZZZ3 (Myb/SANT)	HepG2	M01272_2.00 from Cis-BP	ELK3 (Ets)	1.18%	98.49%	0.32%	930	Tethered binding most frequent

S3 Table. Co-occurring motifs

TF	co-binding TF(s)	tethered binding TF(s)	has same co-binding factors?
FOSL2	BNC2		
CREB1	BNC2,ELF1,SP1,AHDC1,ETS1,SP2		
CEBPB	BNC2,FOXA1,FOXA2,AHDC1		
HNF4G	CEBPB,FOXP4		
NR2C1	ELF1,GABPA		
NR2C2	ELF1,SP1,ZBTB21,ELF4		
E2F8	ELF2,ZBTB21		
ZNF121	EMX1,AR,ERG		
YY1	ETS1,KAT7		
ATF1	ETS1,ZBTB21		
JUNB	FOXA1,AHDC1,ZBTB48,GATAD2A,FOXM1		
ZNF644	FOXA1,CEBPB,ZHX1		
BHLHE40	FOXA1,HIVEP1,ZBTB2		
ETV4	FOXA1,PATZ1,AHDC1		
TFAP4	FOXA1,SP1,ZNF644		
TCF7L2	FOXJ3,ELF3,AHDC1		
ETV5	FOXP4,GATA4		
GMEB1	GABPA,SIX5,ZBTB21		
NR2F1	GATA1,AHDC1		
NR2F6	GATA1,FOXP4,AHDC1		
JUN	GATAD2A,GATA1,FOXP4,ELF3		
FOXA1	GZF1,NR3C1,JUN,AHDC1		
HNF4A	HOMEZ,FOXP4		
ETV6	IRF1,ZBTB49		
TEAD4	JUN,JUNB		
E2F6	MAX		
E2F1	MYBL2,NFYB		
E2F4	MYBL2,REST,ZBTB21,NFYB,SP2		
E2F5	MYBL2,SP1,NFYB,ZBTB21		

MAZ	NFXL1		
SP1	NFYB,NFYA		
SP2	NFYB,NFYA,NFYC,THAP12		
PKNOX1	NFYB,NFYA,SP1,SP2		
USF2	NFYB,SP1,DRAP1,ZBTB21,MAX,NFYA,SP3,CREB3		
CTCF	NKX3-1,ERG		
NFYA	PBX3,NFYB,SP1,SP2		
TCF7	RUNX3,GATA4,AHDC1		
IKZF1	RUNX3,GATA4,NRF1,SIX5		
NFYB	SP1,NFYA,SP2		
USF1	SP1,ZBTB2,DRAP1,ZBTB21,HIVEP1,NFYB		
NRF1	SP5,SP1,ZBTB21,ZBTB2		
ELK1	SRF,ZBTB11		
ZEB1	TCF4,TCF12,EGR2		
NR2F2	TCF7L2,ASCL2,GATA2,ELF3,AHDC1		
ZNF143	THAP11,ETS1		
ELF4	USF2,ZNF143,ZBTB11,SP2		
ETS1	ZNF143,SIX5		
ELF1	ZNF143,SP1,ZBTB21		
SIX5	ZNF76,ZNF143		
FOXK1		ELF2,ELK1,KAT7	
JUND		MAZ,VEZF1,NEUROD1	
SRF		ETV6,JUNB	
ESRRA	SALL1,ZNF644	ZNF790,REST	no
GATA2	HAND2,TCF12,GATA1::TAL1,FOXJ3	ELF3	no
GATA3	HAND2,FOXP4	JUNB,FOXJ3	no
HNF1A	FOXA1	HIVEP1	no
MAX	JUNB,SP1,ETS1,USF2,CREB3	E2F6,ETV6	no
MEF2A	IKZF1,FOSL1,JUN,IRF4	AHDC1	no
MEF2D	ZNF197	FOXJ3	no
MNT	FOXP1,ZSCAN4,SP1	ZBTB2	no
MXI1	RFX1,TGIF2	GABPA,ELF1,RFX1,ZBTB21	no

MYC	ELF1,ETV6,JUNB,CEBPB,ELK4,KLF17,ZNF444,ELK1	MAZ,IKZF3	no
NFIC	ZNF423	TCF7L2,AHDC1	no
RELA	IKZF1,IKZF2,ZNF25	ZGPAT	no
RXRA	FOXA1,FOXP4	JUNB	no
SIX4	AHDC1	GZF1,PATZ1	no
SOX6	MIXL1,AHDC1	GATA2	no
TCF12	ESR1	GATA1,GATA2,AHDC1	no
CREM	ELF1,ETS1,ZBTB21	ELF1,SIX5,ELK4	partial
NR3C1	JUNB,FOXP4,FOSL1,JUN,GATAD2A,CEBPB	IKZF1,JUN	partial
PBX3	NFYB,NFYA,SP2	SP1	partial
STAT3	FOSL2,JUNB,SP5,KLF17,CEBPB	JUNB	partial
ATF3	BNC2	BNC2	yes
MYNN	RFX1	RFX1	yes
RFX5	NFYB	NFYB	yes
SOX13	FOXA1	FOXA1	yes
ZBTB33	ZGPAT	ZGPAT	yes

S4 Table. Summary of TFBSs within 200 bp of CTCF-bound sites

Rank	TF	TF Family	No. of TFBSs	Percentage
1	FOS	bZIP	4625	12.47%
2	JUND	bZIP	4059	10.94%
3	FOSL2	bZIP	3575	9.64%
4	FOXA1	Forkhead	3559	9.60%
5	CEBPB	bZIP	3304	8.91%
6	RXRA	Nuclear receptor	3119	8.41%
7	MAZ	C2H2 ZF	3108	8.38%
8	FOXA2	Forkhead	3107	8.38%
9	IKZF1	C2H2 ZF	2956	7.97%
10	JUN	bZIP	2868	7.73%
11	SPI1	Ets	2842	7.66%
12	JUNB	bZIP	2596	7.00%
13	ELF1	Ets	2462	6.64%
14	NR2F2	Nuclear receptor	2351	6.34%
15	NFIC	SMAD	2336	6.30%
16	FOSL1	bZIP	2143	5.78%
17	HNF4A	Nuclear receptor	2072	5.59%
18	YY1	C2H2 ZF	2023	5.45%
19	EGR1	C2H2 ZF	1938	5.23%
20	GATA3	GATA	1931	5.21%
21	MAX	bHLH	1891	5.10%
22	TEAD4	TEA	1817	4.90%
23	MAFK	bZIP	1815	4.89%
24	NR2F6	Nuclear receptor	1761	4.75%
25	TCF12	bHLH	1751	4.72%
26	STAT3	STAT	1737	4.68%
27	ATF3	bZIP	1700	4.58%
28	TFAP4	bHLH	1607	4.33%
29	GABPA	Ets	1491	4.02%

30	IKZF2	C2H2 ZF	1451	3.91%
31	TCF7L2	HMG/Sox	1442	3.89%
32	FOXA3	Forkhead	1441	3.89%
33	PATZ1	C2H2 ZF; AT hook	1439	3.88%
34	NR3C1	Nuclear receptor	1439	3.88%
35	REST	C2H2 ZF	1434	3.87%
36	CEBPG	bZIP	1432	3.86%
37	GATA2	GATA	1401	3.78%
38	NR2F1	Nuclear receptor	1395	3.76%
39	ZBTB7A	C2H2 ZF	1376	3.71%
40	CEBPA	bZIP	1347	3.63%
41	ZIC2	C2H2 ZF	1337	3.61%
42	USF1	bHLH	1335	3.60%
43	RUNX3	Runt	1324	3.57%
44	GLIS1	C2H2 ZF	1240	3.34%
45	ETV5	Ets	1239	3.34%
46	MAFF	bZIP	1220	3.29%
47	ZGPAT	CCCH ZF	1218	3.28%
48	TEAD3	TEA	1171	3.16%
49	RFX1	RFX	1160	3.13%
50	RXRB	Nuclear receptor	1155	3.11%
51	RXRB	Nuclear receptor	1155	3.11%
52	RXRB	Nuclear receptor	1155	3.11%
53	RXRB	Nuclear receptor	1155	3.11%
54	RXRB	Nuclear receptor	1155	3.11%
55	RXRB	Nuclear receptor	1155	3.11%
56	SP1	C2H2 ZF	1153	3.11%
57	CTCFL	C2H2 ZF	1144	3.08%
58	SOX13	HMG/Sox	1107	2.98%
59	NFE2	bZIP	1075	2.90%
60	ZNF263	C2H2 ZF	1038	2.80%
61	ATF2	bZIP	1029	2.77%

62	PKNOX1	Homeodomain	1023	2.76%
63	MEF2A	MADS box	1016	2.74%
64	ETV6	Ets	1009	2.72%
65	FOXP1	Forkhead	981	2.65%
66	HNF4G	Nuclear receptor	973	2.62%
67	RARA	Nuclear receptor	959	2.59%
68	ELF3	Ets; AT hook	953	2.57%
69	MYC	bHLH	936	2.52%
70	VEZF1	C2H2 ZF	931	2.51%
71	SP5	C2H2 ZF	912	2.46%
72	SOX5	HMG/Sox	904	2.44%
73	NFE2L2	bZIP	880	2.37%
74	EGR2	C2H2 ZF	877	2.36%
75	GATAD2A	GATA	876	2.36%
76	NFIA	SMAD	859	2.32%
77	CREB1	bZIP	852	2.30%
78	ATF7	bZIP	841	2.27%
79	BATF	bZIP	832	2.24%
80	IKZF3	C2H2 ZF	806	2.17%
81	SOX6	HMG/Sox	797	2.15%
82	ZNF143	C2H2 ZF	796	2.15%
83	SCRT2	C2H2 ZF	763	2.06%
84	MAFG	bZIP	753	2.03%
85	E2F6	E2F	751	2.03%
86	ZBTB8A	C2H2 ZF	750	2.02%
87	KLF1	C2H2 ZF	746	2.01%
88	ZNF398	C2H2 ZF	743	2.00%
89	FOXO1	Forkhead	731	1.97%
90	PRDM10	C2H2 ZF	730	1.97%
91	THRA	Nuclear receptor	728	1.96%
92	NRF1	Unknown	725	1.95%
93	SP2	C2H2 ZF	709	1.91%

94	ZNF644	C2H2 ZF	692	1.87%
95	HOMEZ	Homeodomain	689	1.86%
96	SALL1	C2H2 ZF	686	1.85%
97	MIXL1	Homeodomain	682	1.84%
98	PPARG	Nuclear receptor	670	1.81%
99	FOXK1	Forkhead	670	1.81%
100	BCL6	C2H2 ZF	665	1.79%
101	ZSCAN30	C2H2 ZF	660	1.78%
102	USF2	bHLH	654	1.76%
103	ZFX	C2H2 ZF	647	1.74%
104	NFIL3	bZIP	641	1.73%
105	GATA1	GATA	637	1.72%
106	CEBPD	bZIP	629	1.70%
107	SP3	C2H2 ZF	622	1.68%
108	ESRRA	Nuclear receptor	615	1.66%
109	WT1	C2H2 ZF	605	1.63%
110	RBPJ	CSL	603	1.63%
111	TCF3	bHLH	602	1.62%
112	ETV4	Ets	601	1.62%
113	BHLHE40	bHLH	598	1.61%
114	EBF1	EBF1	597	1.61%
115	ESR1	Nuclear receptor	595	1.60%
116	IRF2	IRF	592	1.60%
117	BACH1	bZIP	584	1.57%
118	ZNF148	C2H2 ZF	584	1.57%
119	IRF4	IRF	581	1.57%
120	RREB1	C2H2 ZF	576	1.55%
121	SRF	MADS box	566	1.53%
122	OSR2	C2H2 ZF	556	1.50%
123	CREM	bZIP	545	1.47%
124	RELA	Rel	544	1.47%
125	ZNF331	C2H2 ZF	544	1.47%

126	MEIS2	Homeodomain	536	1.45%
127	POU2F2	Homeodomain; POU	522	1.41%
128	TCF7	HMG/Sox	520	1.40%
129	TFDP1	E2F	509	1.37%
130	ZNF384	C2H2 ZF	509	1.37%
131	NFIB	SMAD	505	1.36%
132	KLF17	C2H2 ZF	503	1.36%
133	FOXK2	Forkhead	502	1.35%
134	ATF4	bZIP	501	1.35%
135	ZNF217	C2H2 ZF	499	1.35%
136	ZNF366	C2H2 ZF	498	1.34%
137	KLF9	C2H2 ZF	494	1.33%
138	ZNF770	C2H2 ZF	490	1.32%
139	MNT	bHLH	489	1.32%
140	RELB	Rel	474	1.28%
141	NR2C2	Nuclear receptor	474	1.28%
142	ERF	Ets	458	1.23%
143	ZBTB20	C2H2 ZF	454	1.22%
144	FEZF1	C2H2 ZF	453	1.22%
145	PAX5	Paired box	450	1.21%
146	TFE3	bHLH	447	1.21%
147	FOXP4	Forkhead	441	1.19%
148	RFX5	RFX	441	1.19%
149	STAT1	STAT	437	1.18%
150	MXI1	bHLH	436	1.18%
151	ETV1	Ets	431	1.16%
152	TBX21	T-box	425	1.15%
153	ZNF281	C2H2 ZF	415	1.12%
154	NFYB	Unknown	414	1.12%
155	ZNF24	C2H2 ZF	413	1.11%
156	THRB	Nuclear receptor	410	1.11%
157	ONECUT1	CUT; Homeodomain	407	1.10%

158	ZNF48	C2H2 ZF	406	1.09%
159	HLF	bZIP	403	1.09%
160	ZNF189	C2H2 ZF	402	1.08%
161	E2F4	E2F	398	1.07%
162	ZNF600	C2H2 ZF	386	1.04%
163	ZBTB17	C2H2 ZF	383	1.03%
164	NFATC3	Rel	376	1.01%
165	KLF8	C2H2 ZF	371	1.00%
166	SMAD5	SMAD	369	0.99%
167	ZNF362	C2H2 ZF	367	0.99%
168	HMG20A	HMG/Sox	364	0.98%
169	ZSCAN9	C2H2 ZF	358	0.97%
170	HIC2	C2H2 ZF	354	0.95%
171	RFX3	RFX	352	0.95%
172	ATF1	bZIP	343	0.92%
173	KLF10	C2H2 ZF	336	0.91%
174	ETS1	Ets	335	0.90%
175	HNF1A	Homeodomain	331	0.89%
176	ZEB2	C2H2 ZF; Homeodomain	323	0.87%
177	ZNF652	C2H2 ZF	323	0.87%
178	ZNF692	C2H2 ZF	322	0.87%
179	TFDP2	E2F	321	0.87%
180	ZXDB	C2H2 ZF	313	0.84%
181	NANOG	Homeodomain	312	0.84%
182	MEF2D	MADS box	310	0.84%
183	ELF4	Ets	308	0.83%
184	MYBL2	Myb/SANT	308	0.83%
185	ZNF205	C2H2 ZF	306	0.83%
186	HIC1	C2H2 ZF	301	0.81%
187	ZNF444	C2H2 ZF	298	0.80%
188	NFYC	Unknown	293	0.79%
189	NR2C1	Nuclear receptor	293	0.79%

190	ZEB1	C2H2 ZF; Homeodomain	286	0.77%
191	ZNF511	C2H2 ZF	281	0.76%
192	ZBTB48	C2H2 ZF	280	0.76%
193	ZNF449	C2H2 ZF	280	0.76%
194	E2F5	E2F	268	0.72%
195	AHDC1	AT hook	266	0.72%
196	ZNF341	C2H2 ZF	264	0.71%
197	SIX1	Homeodomain	260	0.70%
198	SIX4	Homeodomain	257	0.69%
199	ZBTB26	C2H2 ZF	253	0.68%
200	TAL1	bHLH	253	0.68%
201	ZSCAN25	C2H2 ZF	252	0.68%
202	FOXM1	Forkhead	246	0.66%
203	KLF6	C2H2 ZF	245	0.66%
204	LCOR	Pipsqueak	237	0.64%
205	ZFP64	C2H2 ZF	235	0.63%
206	ZNF423	C2H2 ZF	233	0.63%
207	PRDM4	C2H2 ZF	231	0.62%
208	PBX3	Homeodomain	228	0.61%
209	KLF11	C2H2 ZF	224	0.60%
210	FOXP2	Forkhead	222	0.60%
211	ZNF394	C2H2 ZF	222	0.60%
212	DRAP1	Unknown	215	0.58%
213	DLX6	Homeodomain	210	0.57%
214	ZSCAN4	C2H2 ZF	205	0.55%
215	ZFP69B	C2H2 ZF	204	0.55%
216	ZNF740	C2H2 ZF	203	0.55%
217	IRF1	IRF	202	0.54%
218	NFYA	CBF/NF-Y	196	0.53%
219	ZFY	C2H2 ZF	193	0.52%
220	PITX1	Homeodomain	190	0.51%
221	ZNF18	C2H2 ZF	187	0.50%

222	MXD4	bHLH	185	0.50%
223	SP4	C2H2 ZF	182	0.49%
224	MLX	bHLH	181	0.49%
225	ZBTB10	C2H2 ZF	175	0.47%
226	HHEX	Homeodomain	174	0.47%
227	ZFP91	C2H2 ZF	171	0.46%
228	LEF1	HMG/Sox	170	0.46%
229	ZFP37	C2H2 ZF	168	0.45%
230	NR5A1	Nuclear receptor	166	0.45%
231	SCRT1	C2H2 ZF	166	0.45%
232	BCL6B	C2H2 ZF	166	0.45%
233	MEF2B	MADS box	166	0.45%
234	ZNF792	C2H2 ZF	165	0.44%
235	GATA4	GATA	164	0.44%
236	NR5A2	Nuclear receptor	164	0.44%
237	TEAD1	TEA	160	0.43%
238	MZF1	C2H2 ZF	158	0.43%
239	NEUROD1	bHLH	155	0.42%
240	ZBTB21	C2H2 ZF	155	0.42%
241	MXD3	bHLH	153	0.41%
242	ZKSCAN8	C2H2 ZF	151	0.41%
243	ZNF746	C2H2 ZF	147	0.40%
244	NCOA3	bHLH	145	0.39%
245	TBP	TBP	144	0.39%
246	ZNF865	C2H2 ZF	144	0.39%
247	ZNF664	C2H2 ZF	144	0.39%
248	NFE2L1	bZIP	144	0.39%
249	MGA	T-box	143	0.39%
250	ZBTB2	C2H2 ZF	142	0.38%
251	GMEB1	SAND	139	0.37%
252	CUX1	CUT; Homeodomain	138	0.37%
253	ZNF596	C2H2 ZF	137	0.37%

254	PRDM1	C2H2 ZF	136	0.37%
255	KLF4	C2H2 ZF	135	0.36%
256	E2F8	E2F	132	0.36%
257	ELK4	Ets	131	0.35%
258	ONECUT2	CUT; Homeodomain	130	0.35%
259	SIX5	Homeodomain	126	0.34%
260	NFATC1	Rel	123	0.33%
261	HNF1B	Homeodomain	122	0.33%
262	ELK1	Ets	121	0.33%
263	PBX1	Homeodomain	120	0.32%
264	HIVEP1	C2H2 ZF	119	0.32%
265	POU5F1	Homeodomain; POU	117	0.32%
266	POU5F1	Homeodomain; POU	117	0.32%
267	POU5F1	Homeodomain; POU	117	0.32%
268	POU5F1	Homeodomain; POU	117	0.32%
269	POU5F1	Homeodomain; POU	117	0.32%
270	POU5F1	Homeodomain; POU	117	0.32%
271	POU5F1	Homeodomain; POU	117	0.32%
272	ZNF76	C2H2 ZF	115	0.31%
273	ZNF574	C2H2 ZF	115	0.31%
274	MYNN	C2H2 ZF	113	0.30%
275	PBX2	Homeodomain	113	0.30%
276	PBX2	Homeodomain	113	0.30%
277	PBX2	Homeodomain	113	0.30%
278	PBX2	Homeodomain	113	0.30%
279	PBX2	Homeodomain	113	0.30%
280	PBX2	Homeodomain	113	0.30%
281	PBX2	Homeodomain	113	0.30%
282	ARNTL	bHLH	113	0.30%
283	ZBTB6	C2H2 ZF	112	0.30%
284	ZNF121	C2H2 ZF	112	0.30%
285	GFI1	C2H2 ZF	110	0.30%

286	FOXF2	Forkhead	109	0.29%
287	ZNF710	C2H2 ZF	107	0.29%
288	GFI1B	C2H2 ZF	105	0.28%
289	MITF	bHLH	104	0.28%
290	STAT5A	STAT	104	0.28%
291	ZKSCAN1	C2H2 ZF	102	0.28%
292	ZBTB14	C2H2 ZF	98	0.26%
293	SP140L	SAND	98	0.26%
294	ZNF3	C2H2 ZF	97	0.26%
295	SPDEF	Ets	96	0.26%
296	ZNF513	C2H2 ZF	96	0.26%
297	ZFP1	C2H2 ZF	94	0.25%
298	CREB3L1	bZIP	94	0.25%
299	ZNF691	C2H2 ZF	93	0.25%
300	HMBOX1	Homeodomain	92	0.25%
301	ZSCAN5C	C2H2 ZF	91	0.25%
302	E4F1	C2H2 ZF	89	0.24%
303	ZNF512	C2H2 ZF; BED ZF	87	0.23%
304	ZNF629	C2H2 ZF	87	0.23%
305	ATF6	bZIP	86	0.23%
306	ZNF7	C2H2 ZF	85	0.23%
307	THAP9	THAP finger	82	0.22%
308	ZNF660	C2H2 ZF	81	0.22%
309	FOXJ3	Forkhead	78	0.21%
310	HINFP	C2H2 ZF	74	0.20%
311	ZNF124	C2H2 ZF	74	0.20%
312	ZNF580	C2H2 ZF	72	0.19%
313	NR4A1	Nuclear receptor	71	0.19%
314	ZNF316	C2H2 ZF	70	0.19%
315	PRDM15	C2H2 ZF	68	0.18%
316	ZNF524	C2H2 ZF; AT hook	66	0.18%
317	ZBTB42	C2H2 ZF	64	0.17%

318	TGIF2	Homeodomain	64	0.17%
319	ARNT	bHLH	60	0.16%
320	GZF1	C2H2 ZF	60	0.16%
321	KLF13	C2H2 ZF	59	0.16%
322	SMAD1	SMAD	56	0.15%
323	ZBTB40	C2H2 ZF	55	0.15%
324	ZBTB33	C2H2 ZF	55	0.15%
325	SETDB1	MBD	55	0.15%
326	TEF	bZIP	53	0.14%
327	MEIS1	Homeodomain	53	0.14%
328	ZNF558	C2H2 ZF	53	0.14%
329	DDIT3	bZIP	52	0.14%
330	YY2	C2H2 ZF	52	0.14%
331	ZSCAN16	C2H2 ZF	52	0.14%
332	ZNF784	C2H2 ZF	48	0.13%
333	MXD1	bHLH	47	0.13%
334	ZNF646	C2H2 ZF	47	0.13%
335	TFCP2	Grainyhead	46	0.12%
336	ZNF610	C2H2 ZF	45	0.12%
337	E2F1	E2F	45	0.12%
338	ZSCAN29	C2H2 ZF	43	0.12%
339	HMGA2	AT hook	42	0.11%
340	HOXA5	Homeodomain	41	0.11%
341	HOXD13	Homeodomain	41	0.11%
342	IRF9	IRF	40	0.11%
343	ZSCAN22	C2H2 ZF	39	0.11%
344	ZNF175	C2H2 ZF	38	0.10%
345	RUNX1	Runt	38	0.10%
346	ZNF577	C2H2 ZF	38	0.10%
347	ZNF142	C2H2 ZF	37	0.10%
348	ZKSCAN5	C2H2 ZF	37	0.10%
349	OVOL1	C2H2 ZF	37	0.10%

350	GTF2I	GTF2I-like	36	0.10%
351	ZNF777	C2H2 ZF	35	0.09%
352	ZBTB49	C2H2 ZF	34	0.09%
353	GMEB2	SAND	33	0.09%
354	NFIX	SMAD	32	0.09%
355	BCL11A	C2H2 ZF	32	0.09%
356	DMBX1	Homeodomain	31	0.08%
357	ADNP	Homeodomain	31	0.08%
358	ZNF764	C2H2 ZF	31	0.08%
359	ZBTB7B	C2H2 ZF	30	0.08%
360	ZNF503	C2H2 ZF	30	0.08%
361	ZNF680	C2H2 ZF	30	0.08%
362	ZFP3	C2H2 ZF	29	0.08%
363	ZNF549	C2H2 ZF	28	0.08%
364	ZNF34	C2H2 ZF	28	0.08%
365	ZNF329	C2H2 ZF	27	0.07%
366	ZBTB11	C2H2 ZF	26	0.07%
367	HSF2	HSF	26	0.07%
368	ZNF639	C2H2 ZF	25	0.07%
369	ZNF584	C2H2 ZF	24	0.06%
370	ZNF239	C2H2 ZF	24	0.06%
371	ZNF607	C2H2 ZF	24	0.06%
372	ZNF83	C2H2 ZF	23	0.06%
373	TBX3	T-box	23	0.06%
374	ZNF483	C2H2 ZF	23	0.06%
375	ZNF677	C2H2 ZF	22	0.06%
376	RBAK	C2H2 ZF	22	0.06%
377	FOXQ1	Forkhead	22	0.06%
378	ZNF576	C2H2 ZF	21	0.06%
379	DACH1	Unknown	21	0.06%
380	CDC5L	Myb/SANT	21	0.06%
381	ZNF430	C2H2 ZF	20	0.05%

382	ZSCAN12	C2H2 ZF	19	0.05%
383	ZNF761	C2H2 ZF	19	0.05%
384	TEAD2	TEA	18	0.05%
385	ZNF460	C2H2 ZF	18	0.05%
386	ZBTB3	C2H2 ZF	17	0.05%
387	THAP1	THAP finger	16	0.04%
388	ZNF146	C2H2 ZF	16	0.04%
389	ZBTB9	C2H2 ZF	16	0.04%
390	ZBTB9	C2H2 ZF	16	0.04%
391	HOXD1	Homeodomain	16	0.04%
392	ZNF772	C2H2 ZF	16	0.04%
393	SHOX2	Homeodomain	15	0.04%
394	ATF5	bZIP	15	0.04%
395	ZNF215	C2H2 ZF	14	0.04%
396	NKX3-1	Homeodomain	14	0.04%
397	OTX2	Homeodomain	14	0.04%
398	ZBTB24	C2H2 ZF; AT hook	14	0.04%
399	ZNF585B	C2H2 ZF	14	0.04%
400	ZNF589	C2H2 ZF	13	0.04%
401	HOXA10	Homeodomain	13	0.04%
402	ZNF775	C2H2 ZF	13	0.04%
403	ZNF12	C2H2 ZF	12	0.03%
404	ZNF257	C2H2 ZF	12	0.03%
405	TBX18	T-box	11	0.03%
406	ZNF138	C2H2 ZF	11	0.03%
407	ZNF431	C2H2 ZF	11	0.03%
408	ZBTB43	C2H2 ZF	11	0.03%
409	CREB3	bZIP	11	0.03%
410	ZNF583	C2H2 ZF	10	0.03%
411	ZNF436	C2H2 ZF	10	0.03%
412	ZNF133	C2H2 ZF	10	0.03%
413	ZNF79	C2H2 ZF	10	0.03%

414	ATOH8	bHLH	10	0.03%
415	ZNF33B	C2H2 ZF	10	0.03%
416	ZNF816	C2H2 ZF	9	0.02%
417	ZNF184	C2H2 ZF	9	0.02%
418	ZNF41	C2H2 ZF	9	0.02%
419	ZNF668	C2H2 ZF	9	0.02%
420	FOXS1	Forkhead	9	0.02%
421	ZNF319	C2H2 ZF	8	0.02%
422	ZNF790	C2H2 ZF	8	0.02%
423	TSHZ1	C2H2 ZF	8	0.02%
424	ZNF134	C2H2 ZF	7	0.02%
425	CREB5	bZIP	7	0.02%
426	ZNF165	C2H2 ZF	7	0.02%
427	TCFL5	bHLH	7	0.02%
428	ZNF250	C2H2 ZF	7	0.02%
429	ZNF397	C2H2 ZF	7	0.02%
430	E2F3	E2F	7	0.02%
431	ZNF140	C2H2 ZF	7	0.02%
432	SNAPC4	Myb/SANT	7	0.02%
433	TP53	p53	7	0.02%
434	ZNF23	C2H2 ZF	6	0.02%
435	ZBTB34	C2H2 ZF	6	0.02%
436	SMAD3	SMAD	6	0.02%
437	HOXB13	Homeodomain	6	0.02%
438	SATB2	CUT; Homeodomain	6	0.02%
439	ZNF160	C2H2 ZF	5	0.01%
440	ZNF337	C2H2 ZF	5	0.01%
441	HOXB5	Homeodomain	5	0.01%
442	ZNF75A	C2H2 ZF	5	0.01%
443	EEA1	C2H2 ZF	5	0.01%
444	ZNF445	C2H2 ZF	4	0.01%
445	ZFP14	C2H2 ZF	4	0.01%

446	KLF12	C2H2 ZF	4	0.01%
447	BHLHA15	bHLH	4	0.01%
448	ZNF25	C2H2 ZF	3	0.01%
449	ZNF26	C2H2 ZF	3	0.01%
450	ZNF707	C2H2 ZF	3	0.01%
451	EMX1	Homeodomain	3	0.01%
452	ZHX1	Homeodomain	3	0.01%
453	ZKSCAN3	C2H2 ZF	2	0.01%
454	ZNF274	C2H2 ZF	2	0.01%
455	YBX1	CSD	2	0.01%
456	ZBTB5	C2H2 ZF	2	0.01%
457	ZNF490	C2H2 ZF	2	0.01%
458	ZSCAN20	C2H2 ZF	2	0.01%
459	ZNF318	C2H2 ZF	2	0.01%
460	THAP12	THAP finger	2	0.01%
461	DMTF1	Myb/SANT	2	0.01%
462	ZNF562	C2H2 ZF	2	0.01%
463	ZNF302	C2H2 ZF	2	0.01%
464	ZNF20	C2H2 ZF	2	0.01%
465	ZNF624	C2H2 ZF	2	0.01%
466	ELF2	Ets	1	0.00%
467	ZNF324	C2H2 ZF	1	0.00%
468	ZNF232	C2H2 ZF	1	0.00%
