# Supplementary material for: Positional distribution of transcription factor binding sites in the human genome
Source: PLoS One. 2025 Jul 30;20(7):e0329226. doi: 10.1371/journal.pone.0329226 (PMC12310040; doi:10.1371/journal.pone.0329226)
Supplement: Attachment — Submitted filename: Responses-to-reviews-final.docx [file pone.0329226.s004.docx]

**Responses to reviews**

We are grateful to both reviewers for their helpful comments, which have been helpful for improving our manuscript. Please find our point-by-point responses below. Our revisions in the manuscript are highlighted in red.

**Reviewers’ comments:**

Reviewer #1:

1. In Abstract, the definition of ENCODE and CTCF should be provided when these two words first appear.

Reply: Thanks for your suggestion. We have revised the Abstract to include the definitions of the two abbreviations

- For ENCODE, we now state: "Using the chromatin immunoprecipitation sequencing data recently released by ENCODE (Encyclopedia of DNA Elements), we obtained …”
- For CTCF, we revised it to: “Many TFBSs lie close to CTCF (CCCTC-binding factor) binding sites.”

2. In Introduction, the definition of CTCF should be provided when it first appears.

Reply: The definition of CTCF has been added upon its first appearance in the Introduction: “…distribution in functional regions including enhancer regions, CTCF (CCCTC-binding factor) binding sites, and promoter regions.”

3. In Inferring canonical and co-occurring motif sections, a “ is missing (“candidate canonical PWM).

Reply: Thank you for catching this oversight. The missing quotation mark has been added: “…if the top PWM is not similar to any known PWM in all other TF families, then we consider it a “candidate canonical PWM.”

4. How do the authors define TF families?

Reply: The TF families are defined according to the classification of Lambert et al.[9]. We have added this qualification in our revised manuscript.

5. The definition of a co-occurring motif (in Inferring canonical and co-occurring motif sections) is strange. The definition that the authors used seems to have nothing to do with co-occurrence. Moreover, are co-occurring motifs (defined by the authors) biologically interesting?

Reply: Our definition of “co-occurring” is “a co-occurring motif refers to a situation in which a motif found in the ChIP-seq data of a TF (TF1) is similar to the canonical motif of a second TF (TF2) that belongs to another TF family.” It is a situation sometimes observed in ChIP-seq data. Why a TF2 motif should be observed in the ChIP-seq data specifically obtained for TF1 is a phenomenon that should be explained; note that the ChIP-seq fragment length is only ~200 bp, so that the two motifs are very close to each other. Indeed, this phenomenon has been discussed in the literature. In our study, we went into some details to classify different situations of co-occurring and their possible mechanisms, trying to provide a better understanding of motif co-occurring. Our study reveals that *in vivo* TF-DNA binding is far more complex than *in vitro* TF-DNA binding.

6. The usage of co-occurring motifs and co-binding motifs is confusing. What are the differences between these two terms?

Reply: Indeed, the two terms are confusing. Their definitions are as follows:

**Co-occurring motifs** refer to the situation where two different motifs are present on the same ChIP-seq fragment. This is unexpected because only one type of TF antibody was used to pull down the DNA fragment.

**Co-binding motifs** refer to the types of TF-DNA binding mechanism. There are two types of TF-DNA binding, which are defined as follows:

- **Co-binding:** Two different TFs, TF1 and TF2, bind to the same ChIP-seq fragment.
- **Tethered binding:** This refers to the situation where TF1 does not bind to DNA directly but instead binds to TF2, which binds to DNA. In this specific case, the canonical motif of TF1 is not found on the ChIP-seq fragment.

In a set of ChIP-seq data, one may have the following two types of observations. First, two types (TF1 and TF2) of TF binding motifs are observed on a ChIP-seq fragment. In this case, it is interpreted as that TF1 and TF2 both bound to the same ChIP-seq fragment. Second, only the TF2 motif but no TF1 motif is present on a ChIP-seq fragment. This situation is interpreted to be due to tethered binding. That is. TF1 did not directly bind to DNA but bound to TF2, which bound to DNA.

To say tethered binding is a co-binding is a bit of misleading because only one TF binds to DNA. However, as the two terms have been commonly used in the literature, it is not simple to change.

In our study, we tried to quantify the relative frequencies of co-binding and tethered binding.

7. The authors classified 550 TFs into five groups. Please provide a browsable table in dbTFBS v2 so that users can easily access this information.

Reply: Thank you for this excellent suggestion. We have made this data available for download within our database, allowing users to easily access the classification of the 550 TFs. We also added the folllowing sentence in the "Data availability" section: “The classification of TFs based on co-binding frequency and biological features, and the locations of identified TFBSs, are also available for download in dbTFBS v2.”

8. The authors clustered the TFs into six groups (Fig 5B). Please provide a browsable table in dbTFBS v2 so that users can easily access this information.

Reply: We appreciate your suggestion. Similar to the previous point, the data corresponding to the six TF clusters from Figure 5B has been stored in our database and is available for users to download.

9. It would be great if the authors could provide a genome browser in dbTFBSv2 to show the genome locations and the target gene names of a TFBS of interest.

Reply: Thanks. We agree that a genome browser would be a valuable addition. However, a direct integration into our website presents technical challenges at this time. Therefore, we have compiled all our TFBS data into the bigBed format. This allows users to easily upload our data as a custom track to the UCSC Genome Browser, where they can visualize genome locations and associated information.

Reviewer #2: In this study, the authors used ENCODE ChIP-Seq data to infer position weight matrices (PWMs) for 500 transcription factors (TFs) across 44 TF families. Based on these data, they analyzed TF interactions, TF cofactors, the relative frequencies of co-binding and tethered binding, and the positional distribution of transcription factor binding sites (TFBSs) in the human genome. Their findings reveal that TF interactions are more frequent and complex than previously thought.
The manuscript is well-organized and clearly written. The findings provide novel insights into transcription regulation in human. However, a few issues should be addressed before publication:
1. For readers unfamiliar with transcription factors, the authors should include an annotation for CTCF(CCCTC-binding factor) when CTCF first appears.

Reply: Thanks for this valuable suggestion. We agree that defining CTCF is important for clarity. As in our reply to Reviewer #1, we've added the annotation for CTCF (CCCTC-binding factor) to its first appearance in both the Abstract and the Introduction.

- In the **Abstract**, we revised the sentence to: "Many TFBSs lie close to CTCF (CCCTC-binding factor) binding sites."
- In the **Introduction**, the revision now reads: "...distribution in functional regions including enhancer regions, CTCF (CCCTC-binding factor) binding sites, and promoter regions."

2.In the PCA plot (Fig. 5B), the x- and y-axes should be relabeled as PC1 and PC2. The authors selected five cut-points in PC1 to divide TFs into six groups. The reason should be explained for clarity. Why not just divided into two groups in zero point?

Reply: We appreciate you pointing this out. We've updated the PCA plot (Fig. 5B) to clearly label the axes as PC1 and PC2.

Regarding the decision to divide TFs into six groups, our aim was to uncover finer structures and progressive patterns within TF families. While a simple division at the zero point of PC1 would yield two groups, using multiple cut-points allows us to observe more nuanced variations in how TFBSs are distributed across the four biological regions. We've clarified this motivation in the manuscript: "As the TFBSs of a TF show an uneven distribution in the four biological regions, we cluster the TFs into six groups based on the FC values using PCA (principal component analysis) for finer progressive patterns (Fig 5B)."
